# Mapping eQTLs with RNA-seq reveals novel susceptibility genes, non-coding RNAs and alternative-splicing events in systemic lupus erythematosus

**DOI:** 10.1093/hmg/ddw417

**Published:** 2017-01-05

**Authors:** Christopher A. Odhams, Andrea Cortini, Lingyan Chen, Amy L. Roberts, Ana Viñuela, Alfonso Buil, Kerrin S. Small, Emmanouil T. Dermitzakis, David L. Morris, Timothy J. Vyse, Deborah S. Cunninghame Graham

**Affiliations:** 1 Department of Medical & Molecular Genetics, King’s College London, London, UK; 2 Department of Twin Research, King’s College London, London, UK; 3 Department of Genetic Medicine and Development, University of Geneva Medical School, Geneva, Switzerland; 4 Institute of Genetics and Genomics in Geneva, University of Geneva, Geneva, Switzerland; 5 Swiss Institute of Bioinformatics, Geneva, Switzerland; 6 Division of Immunology, Infection and Inflammatory Disease, King’s College London, London, UK

## Abstract

Studies attempting to functionally interpret complex-disease susceptibility loci by GWAS and eQTL integration have predominantly employed microarrays to quantify gene-expression. RNA-Seq has the potential to discover a more comprehensive set of eQTLs and illuminate the underlying molecular consequence. We examine the functional outcome of 39 variants associated with Systemic Lupus Erythematosus (SLE) through the integration of GWAS and eQTL data from the TwinsUK microarray and RNA-Seq cohort in lymphoblastoid cell lines. We use conditional analysis and a Bayesian colocalisation method to provide evidence of a shared causal-variant, then compare the ability of each quantification type to detect disease relevant eQTLs and eGenes. We discovered the greatest frequency of candidate-causal eQTLs using exon-level RNA-Seq, and identified novel SLE susceptibility genes (e.g. *NADSYN1* and *TCF7*) that were concealed using microarrays, including four non-coding RNAs. Many of these eQTLs were found to influence the expression of several genes, supporting the notion that risk haplotypes may harbour multiple functional effects. Novel SLE associated splicing events were identified in the T-reg restricted transcription factor, *IKZF2*, and other candidate genes (e.g. *WDFY4*) through asQTL mapping using the Geuvadis cohort. We have significantly increased our understanding of the genetic control of gene-expression in SLE by maximising the leverage of RNA-Seq and performing integrative GWAS-eQTL analysis against gene, exon, and splice-junction quantifications. We conclude that to better understand the true functional consequence of regulatory variants, quantification by RNA-Seq should be performed at the exon-level as a minimum, and run in parallel with gene and splice-junction level quantification.

## Introduction

Genome-Wide Association Studies (GWAS) have successfully identified a large number of genetic loci that contribute to complex-disease susceptibility in humans (1). Evidence suggests these variants are enriched within regulatory elements of the genome and their effects play a central role in modulation of intermediate quantitative phenotypes such gene expression (1–6). Many expression quantitative trait loci (eQTL) mapping studies have since been conducted across a wide-range of ethnicities (7,8), cell-types (9–16), disease states (17–22) and in response to various environmental stimuli (23,24)—with each contributing to our understanding of the architecture of human regulatory variation in complex-disease.

In spite of diverse study designs, a significant constraint on the majority of such investigations is the use of 3´-targeted microarrays to profile gene expression. The effects of splicing are less likely to be detected through quantification of pre-defined probes that target common exons of a gene (25) and may explain why only a limited number of susceptibility loci localize to causal eQTL signals (26,27). Technical limitations of microarrays and noise from the small probe design of exon-arrays, further hinder the accuracy of expression measurements (25,28–30). RNA-Seq based eQTL mapping studies are beginning to emerge (31,32) and, although large-scale analysis pipelines are still being streamlined, such types of investigations will greatly increase the likelihood of capturing disease associated eQTLs as quantification of overall gene and independent exon expression, as well as relative transcript abundance (including novel isoforms and non-coding RNAs) is possible (33–39).

Integrative studies using RNA-Seq to functionally annotate complex-disease susceptibility loci however have been limited (35,40–44). Direct comparison of eQTLs between studies is also complicated by the diverging strategies used to map sequenced reads to their genomic origins, how multi-mapping and exon-exon spanning reads are dealt with, the choice of reference genome assemblies and genomic annotations, and finally the methods used to infer gene abundance (45–47). Moreover, numerous investigations have aimed to explain the functional relevance of susceptibility loci by interrogation of GWAS SNPs themselves in eQTL datasets and simply testing for association with gene expression (48–50). Such inferential observations should be treated with caution as they may possibly be the result of coincidental overlap between disease association and eQTL signal due to local LD and general ubiquity of regulatory variants (51). This has become particularly important as statistical power in eQTL cohorts grow and availability of summary-level data accession through eQTL data-browsers increases (52–54).

In this investigation, we integrate eQTL data derived from both microarray and RNA-Seq experiments with our GWAS results in Systemic Lupus Erythematosus (SLE [MIM: 152700]); a heritable autoimmune disease with undefined aetiology and over 50 genetically associated loci (55–57). We use summary-level *cis*-eQTL results in lymphoblastoid cell lines (LCLs) taken from the TwinsUK cohort to directly compare the microarray (9) and RNA-Seq (39) results in detecting SLE associated eQTLs along with their accompanying eGenes. We apply a rigorous two-step approach – a combination of conditional (58) and Bayesian colocalisation (59) analysis – to test for a shared causal variant at each locus. We demonstrate the benefits of using RNA-Seq over microarrays in the eQTL analysis by identifying not only novel SLE candidate-causal eGenes but also putative molecular mechanisms by which SLE-associated SNPs may act; including differential exon usage, and expression modulation of non-coding RNA. Our investigation was extended to include RNA-Seq expression data in whole blood in order to validate the eQTL signals detected in LCLs and uncover the differences in genetic control of expression between cell-types. Finally, we interrogate the Geuvadis RNA-Seq cohort (35) to identify SLE associated alternative-splicing quantitative trait loci (asQTLs) and highlight the advantages of profiling with a multitude resolutions to detect eQTLs that would otherwise remain concealed. Through functional annotation of SLE associated loci using microarray and RNA-Seq derived expression data, we have supplied comprehensive evidence of the need to use RNA-Seq, principally at exon-level resolution, to detect disease contributing eQTLs and, in doing so, have suggested novel functional mechanisms that serve as a basis for future targeted follow-up studies.

## Results

### Discovery and classification of SLE candidate-causal eQTLs and eGenes

**Figure 1.**
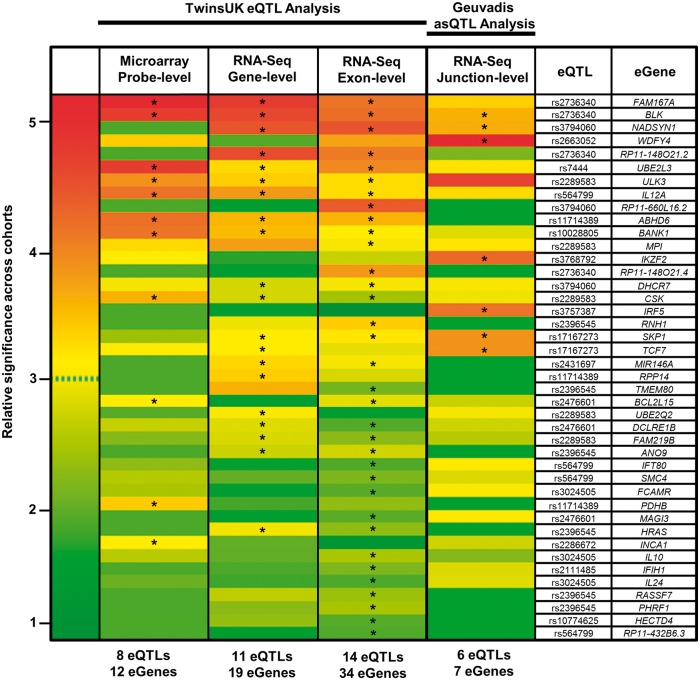
Heatmap of candidate-causal eQTLs and eGenes detected across the four expression-quantification types. Relative association *P*-values are shown. If a candidate-causal association (marked *) is identified in at least one quantification type, then the *P*-value is shown for all quantifications (no * means the association is not candidate-causal). Rows are ordered by decreasing cumulative significance. To normalize across quantification types, relative significance of each association per column was calculated as the –log_2_ (*P*/*P*_max_); where *P*_max_ is the most significant association per quantification type. Data used for heatmap are found in Supplementary Materials, Tables S2, S3, S4, S8 for microarray, gene-level, exon-level, and splice-junction level eQTL analysis respectively.

We integrated the 39 SLE associated SNPs taken from our recent GWAS in Europeans (Supplementary Material, Table S1) with eQTLs from the TwinsUK gene-expression cohort profiled using microarray and RNA-Seq (at both gene-level and exon-level resolutions—Table 1). To accomplish this, we subjected the genomic intervals within +/-1Mb of the 39 GWAS SNPs to eQTL association analysis against expression quantifications in LCLs then tested statistically for evidence of a shared causal variant between the disease-association and eQTL signal (see Methods). Exons (‘meta-exons’, created by merging all overlapping exonic portions of a gene into non-redundant units) were quantified using read-counts against the GENCODE v10 annotation; with gene-level quantification defined as the sum of all exon quantifications belonging to the same gene. Full results of the conditional and colocalisation analysis for each significant association are presented in Supplementary Materials, Tables S2–S4 for microarray, RNA-Seq (gene-level), and RNA-Seq (exon-level), respectively. Statistically significant SLE-associated eQTLs showing evidence of a shared causal variant or in strong LD between the disease and eQTL signal following conditional and colocalisation analyses were classified as SLE candidate-causal eQTLs. Candidate-causal eGenes were defined as genes whose expression is modulated by the eQTL. These results are summarised as a heatmap under the TwinsUK eQTL analysis header in Fig. 1 with candidate-causal associations highlighted.

**Table 1.** Details of genotype-expression (eQTL) cohorts used in study

Cohort Name	**TwinsUK**				**Geuvadis**
Total subjects	856				373
Ethnicity	EUR (UK)				EUR (CEU, GBR, FIN, TSI)
Sex	F				M/F
Age	37–85				*NA*
Investigation	Comparison of candidate-causal eQTL and eGene detection between microarray and RNA-Seq			Validation and comparison of LCL RNA-Seq discoveries in whole blood	Identification of asQTLs using RNA-Seq

Citation	*Grundberg et. al* (9)	*Buil et. al* (39)	*Buil et. al* (39)	*Buil et. al* (39)	*Lappalainen et. al* (35)
Expression profile type	Microarray	RNA-Seq	RNA-Seq	RNA-Seq	RNA-Seq
Unit of expression	Probe	Gene	Meta-exon	Meta-exon	Splice-junction
Cell-type	LCL	LCL	LCL	Whole Blood	LCL

Subjects used in analysis	777	683	765	384	373
Data format	Genevar (summary results)	Read-count	Summary eQTL results	Summary eQTL results	Raw sequence alignments
RNA Platform	Illumina HT-12 V3	Illumina HiSeq2000		Illumina HiSeq2000	Illumina HiSeq2000
RNA-Seq mapper	*NA*	BWA v0.5.9 (GRCh37/hg19)		BWA v0.5.9 (GRCh37/hg19)	GEM v1.349 (GRCh37/hg19)
Reference transcriptome	*NA*	GENCODE V10		GENCODE V10	GENCODE V10
RNA-Seq read length	*NA*	49-bp PE		49-bp PE	75-bp PE

Breakdown of genotype-expression (eQTL) cohorts used in analysis. TwinsUK cohort in lymphoblastoid cell lines (LCLs) used for microarray and RNA-Seq comparison (profiled at gene and meta-exon resolution); meta-exons are described as non-redundant overlapping portions of exons generated flattening of the transcriptome annotation. All TwinsUK (MuTHER) samples used in analysis are derived from the original 856 individuals. Validation of LCL data in whole blood carried out at meta-exon level using 384 of the 856 individuals. Geuvadis cohort used for asQTL identification; splice-junction quantifications were generated by Altrans (57) from the raw sequence alignments. Summary eQTL results include only the eQTL association results per test (where full genotype and expression data were not obtainable).

### Exon-level quantification yields the highest frequency of candidate-causal eQTLs and can be used to infer disease-associated isoforms

Figure 1 illustrates the clear improvement of RNA-Seq relative to microarray in the discovery of candidate-causal eQTLs and their corresponding eGenes when annotating complex-disease susceptibility loci. In total, 8 eQTLs regulating expression of 27 eGenes were detected using RNA-Seq but missed using microarray (Supplementary Material, Fig. S1). Only one eQTL (rs2286672) and two eGenes (*PDHB*, *INCA1*) were found by microarray only. These associations were either not significant post multiple testing using either RNA-Seq method, or were not deemed candidate-causal (Supplementary Materials, Tables S3–S4). Exon-level RNA-Seq analysis led to the greatest frequency of candidate-causal eQTLs and eGenes. A total of 14 eQTLs modulating expression of 34 eGenes were detected using exon-level RNA-Seq contrasted to 11 eQTLs and 19 eGenes at gene-level RNA-Seq and only 8 eQTLs with 12 eGenes identified using microarray. Interestingly, exon-level analysis led to the greatest frequency of non-candidate-causal associations. Only 14 of the 34 significant associations (q < 0.05) showed evidence of a shared causal variant post conditional and colocalisation testing (Supplementary Material, Fig. S1).

**Figure 2.**
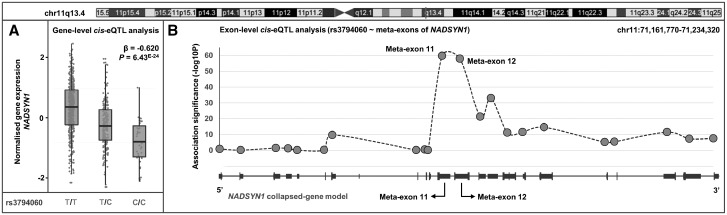
Gene-level and exon-level analysis implicate *NADSYN1* as a candidate-causal eGene. (**A**) eQTL analysis of rs3794060 reveals the risk variant [C] leads to down-regulation at the gene-level of *NADSYN1*. (**B**) Exon-level quantification leads to inference of gene-level effect being driven by expression disruption of two meta-exons of *NADSYN1* (meta-exon 11 and meta-exon 12). Association *P*-values of rs3794060 against exon quantifications are plotted with reference to the specific exon in the collapsed-gene model of *NADSYN1* (all annotated transcripts combined).

We were able to leverage the resolution of exon-level eQTL analysis to map associations back to specific gene isoforms and investigate potential splicing mechanisms (Supplementary Material, Table S5). An example of this is illustrated in Fig. 2. The risk variant rs3794060 [C] was classified as being a candidate-causal eQTL for eGene *NADSYN1* (NAD Synthetase 1) at both gene- and exon-level. Alignment of exon-level associations against the 22 annotated transcripts of *NADSYN1* suggests a potential splicing mechanism largely affecting meta-exons 11 and 12 (*P *=* *1.79×10^−^^60^, 1.06×10^−^^58^ respectively) which are unique to the single isoform ENST00000528509. With the same methodology, we were also able to identify potential whole-gene effects where every single exon of a gene and thus every transcript is modulated. This was found for example in rs2476601 where all six transcripts of *BCL2L15* comprised a differentially expressed meta-exon (Supplementary Material, Table S5).

### Exon-level RNA-seq uncovers regulatory variants associated with multiple functional effects

Exon-level eQTL analysis generated the greatest ratio of candidate-causal eGenes to eQTLs (2.42) suggesting that disease-associated haplotypes may be more functionally potent than previously thought. In comparison, an eGene to eQTL ratio of 1.73 was observed using gene-level quantification and just 1.5 with microarray. A maximum of six eGenes associated with a single eQTL was identified at exon-level for the GWAS SNP rs12802200 (*HRAS***,***TMEM80*, *RNH1*, *ANO9*, *PHRF1* and *RASSF7*); which supports our recent observations of rs12802200 being a *cis*-eQTL for multiple genes across various immune cell-types at this locus (60)**.** This locus has also been shown to correlate with increased autoantibody production and interferon-α activity in sufferers of SLE (61), thus dysregulation of multiple target genes represents an intriguing mechanism for functional investigation. Other candidate-causal eQTLs with multiple functional effects that were detected using RNA-Seq but not microarray are as follows: rs2476601 (*DCLRE1B*, *BCL2L15*, *MAGI3*), rs3024505 (*IL10*, *IL24*, *FCAMR*), rs564799 (*IL12A*, *SMC4*, *IFT80*, *RP11-432B6.3*), rs7726414 (*TCF7*, *SKP1*), and rs3794060 (*DHCR7*, *NADSYN1*, *RP11-66L16.2*). Following integration of known Topologically Associated Domains (TADs) from both IMR90 and h1-eESC cells of the ENCODE Project, we found that multiple eGenes under the control of a solitary eQTL were always found in the same TAD and did not cross TAD boundaries; suggesting proximal looping interactions within a TAD may be perturbed by a single eQTL.

### RNA-seq underlines the role of non-coding RNA in SLE

Quantification of polyadenylated non-coding RNAs from the TwinsUK RNA-Seq cohort revealed three candidate-causal eQTLs influencing the expression of four non-coding eGenes (Supplementary Material, Table S4); none of which were captured using microarray.

**Figure 3.**
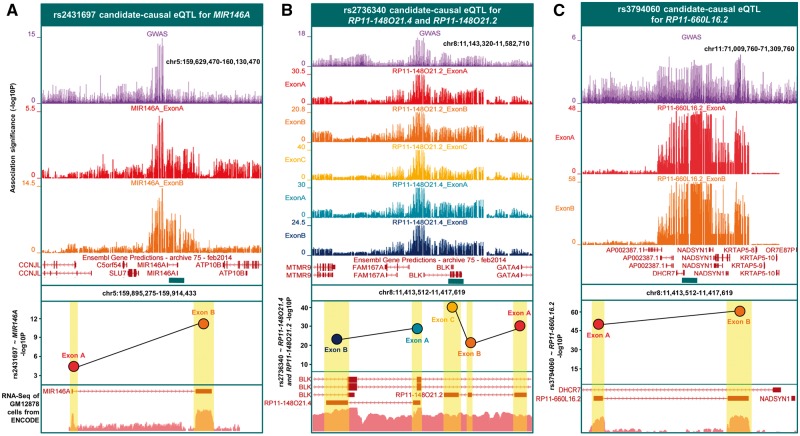
Non-coding candidate-causal eGenes detected by exon-level RNA-Seq. Three panels denote the eQTLs and corresponding non-coding eGenes identified from eQTL analysis against exon-level quantifications. The top panels display the signal from the GWAS association plotted as –log_10_ (*P*), with the exon-level eQTL *P*-values for the effects showing colocalisation with the GWAS signal. The bottom panel shows RNA-Seq expression from ENCODE (GM12878). (**A**) rs2431697 is a candidate-causal eQTL for *MIR146A*. (**B**) rs2736340 is a candidate-causal eQTL for *RP11-148O21.4* and *RP11-148O21.2*. (**C**) rs3794060 is a candidate-causal eQTL for *RP11-6OL16.2*.

We validated the function of known association rs2431697 (Fig. 3A); where the protective minor allele [C] leads to upregulation of the miRNA *MIR146A*, a negative regulator of the type I Interferon pathway (62). rs2431697 was the best eQTL for *MIR146A* at gene-level (*P *=* *1.5×10^−^^06^) and at exon level for both of its exons (*P *=* *3.4×10^−^^12^ and 1.2×10^−^^04^). The decrease in expression of *MIR146A* reported in peripheral blood leukocytes of SLE patients disrupts binding of transcription factor Ets-1 which in turn uncouples of the type-1 IFN response (62).

We discovered a novel function of rs2736340, located in the bi-directional promoter region of eGenes *BLK* and *FAM167A* (Fig. 3B). We replicated the known effect of rs2736340 which leads to increased expression of *FAM167A* and decreased expression of *BLK*, causing altered B-cell development (63). Interestingly however, exon-level RNA-Seq revealed rs2736340 also modulates the expression of two non-coding RNAs antisense to the 3′ region of *BLK*. These are: *RP11-148O21.2* and *RP11-148O21.4*. rs2736340, significantly modulated the expression of all three exons of *RP11-148O21.2* and the two exons of *RP11-148O21.4*. Expression disruption of these antisense RNAs caused by SLE risk variants present an additional functional mechanism at this locus.

Likewise, rs3794060 leads to allele-dependent expression variation of both exons of antisense non-coding RNA, *RP11-660L16.2I* (Fig. 3C). The best eQTL for the two exons is highly correlated with rs3794060 (rs2282621, *r*^2^:0.99). *RP11-660L16.2I* is located in the bi-directional promoter between *DHCR7* and *NADSYN1*. Both were also defined as being candidate-causal eGenes detected using RNA-Seq. These findings using exon-level RNA-Seq support our proposition of risk haplotypes causing multiple functional effects—in the latter two cases simultaneous modulation of protein-coding genes as well as non-coding RNAs.

### Validation of exon-level candidate-causal eQTLs using whole-blood RNA-seq

To validate our eQTL discoveries from lymphoblastoid cell lines in a primary tissue-type, we extended our analysis to include the TwinsUK exon-level RNA-Seq dataset of 384 individuals profiled in whole-Blood (Table 1). Full results are provided in (Supplementary Material, Table S6). We observed good correlation of effect-sizes (β) between LCLs and whole-blood for all matched exon-level associations (*R*^2^^* *^=^* *^0.74, Supplementary Material, Fig. S2). Seven of the 39 GWAS SNPs were classified as candidate-causal eQTLs in whole-blood, modifying 19 candidate-causal eGenes. All seven of the whole-blood eQTLs and 15 of the 19 eGenes were deemed candidate-causal in LCLs, suggesting strong conservation across whole-blood cell types (Supplementary Material, Fig. S3). The remaining four eGenes specific to whole-blood were: *PXK* (rs9311676); *IRF7* and *TALDO1* (rs12802200); and *SCAMP2* (rs2289583). Interestingly, the eQTLs regulating these four eGenes in whole-blood also regulated multiple eGenes in LCLs, implying they may tag highly regulatory haplotypes that cause cell-type specific expression disruption across multiple genes. Three of the four candidate-causal non-coding eGenes from LCLs were found in whole-blood (*RP11-148O21.2*, *RP11-148O21.4*, and *RP11-660L16.2*). *MIR146A* was not significant, which is likely to be a result of its lymphocyte-specific gene expression profile (62). We further validated these whole-blood associations in an entirely independent dataset by eQTL interrogation of the GTEx cohort (64) in whole-blood (n = 393). Five of the seven whole-blood associations in the TwinsUK cohort were significant eQTLs for at least one eGene in GTEx whole blood (Supplementary Material, Table S7).

Inspection of specific exons modulated by candidate-causal eQTLs between each cell-type revealed instances of variability in the genetic control of exon usage. A known splicing event in B-cells caused by branch-point SNP rs17266594 results in the loss of exon 2 in *BANK1* and subsequently leads to B-cell hyper-responsiveness (65) (Supplementary Material, Fig. S4). In whole-blood the GWAS variant, rs10028805, is associated with altered expression of exon 2 (*P *=* *8.4×10^−^^05^), with the best eQTL for this effect being in near-perfect LD (rs4411998; *r*^2^:0.98). Both rs10028805 and rs4411998 are in strong LD with the branch-point SNP rs17266594 (*r*^2 ^>^ ^0.9). In LCLs however, the best eQTL for exon 2, rs4572885 (*P *=* *9.74 ×10^−^^23^), has a large effect but is less correlated with the GWAS SNP (*r*^2^:0.65) and conditional analysis judges this association to be independent of the best eQTL for exon 2. Interestingly, there is low correlation between the branch-point SNP rs17266594 and the best eQTL for exon 2 in LCLs (*r*^2^:0.42); suggesting the regulatory mechanism of exon 2 splicing in *BANK1* may be under two separate genetic influences between the two cell-groups.

We saw a near identical pattern of differential exon usage of *NADSYN1* between LCLs and whole-blood driven by rs37940460 (Supplementary Material, Fig. S5). rs37940460 leads to extensive expression disruption of two meta-exons (11 and 12) of *NADSYN1* located near the centre of the gene (meta-exon 11: LCL *P *=* *1.79×10^−^^60^; whole-blood *P *=* *1.28×10^−^^27^; meta-exon 12: LCL *P *=* *1.06×10^−^^58^; whole-blood *P *=* *6.30×10^−^^26^). This novel example of specific exon expression disruption, validated in a primary cell-type, will help to resolve the functional consequence of the *NADSYN1* locus.

### asQTL mapping reveals additional candidate-causal eGenes and alternative-splicing events

We extended our investigation to determine whether interrogation of alternative-splicing quantitative trait loci (asQTLs), would reveal any additional candidate-genes or potential functional mechanisms. We undertook *cis*-asQTL analysis within a +/-1Mb window around each SNP against 33,039 splice-junction quantifications, corresponding to 817 genes, using the Geuvadis cohort (Table 1). After testing for a shared causal variant between the GWAS and asQTL signal, six SLE candidate-causal asQTLs for 26 splice-junctions corresponding to seven eGenes remained (Supplementary Material, Table S8). Four eGenes (*TCF7*, *SKP1*, *BLK*, and *NADSYN1*) had previously been detected through eQTL mapping using the TwinsUK cohort, the remaining three candidate-causal eGenes (*IKZF2*, *WDFY4*, and *IRF5*) were detected by asQTL mapping solely.

**Figure 4.**
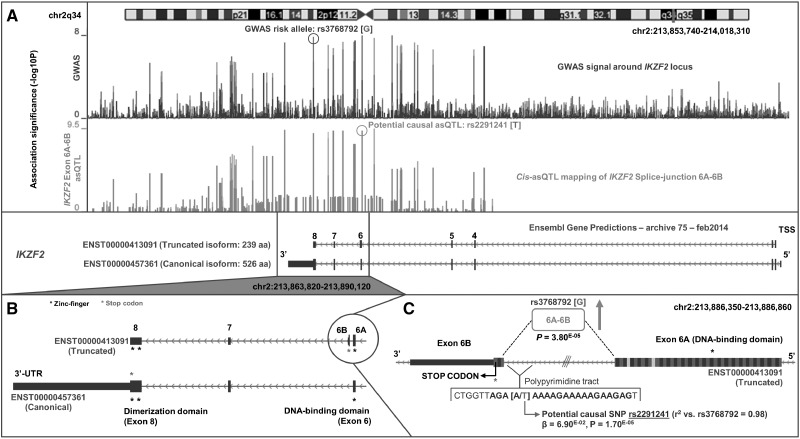
Novel eGene *IKZF2* and potential causal mechanism using splice-junction quantification. asQTL analysis of rs3768792 against splice-junction quantifications identifies *IKZF2* as a candidate-causal eGene with risk variant [G] causing upregulation of the exon 6A–exon 6B junction that is unique to truncated isoform ENST00000413091. A) GWAS association signal across the *IKZF2* locus (chr2q34), tagged by rs3768792 localised in the 3′-UTR of *IKZF2*. asQTL association signal of rs3768792 against splice-junction quantification of exon 6A–exon 6B shows significance and colocalisation with the GWAS signal. B) The exon 6A–exon 6B junction is unique to truncated isoform ENST00000413091. Exon 6B harbours a premature stop-codon and therefore is not translated into the full-length protein that contains the dimerization domains in exon 8. C) Close-up of the exon 6A–exon 6B junction and association (*P *=* *3.80 × 10^−05^) with GWAS SNP rs3768792. A potential causal asQTL in near-perfect LD was identified that is located within the polypyrimidine tract of the junction and may induce splicing (rs2291241, *P *=* *1.70 × 10^−05^).

*IKZF2* is novel candidate-causal eGene detected only by asQTL analysis. The GWAS association signal around the 3′ end of *IKZF2* tagged by risk variant rs3768792[G] drove an increase in the fraction of splicing between exon 6A and exon 6B (*P *=* *3.8×10^−^^05^); a bridge that is unique to the truncated isoform (ENST00000413091, 239 amino-acids) of *IKZF2* (Fig. 4). Interestingly, this isoform possesses a premature termination codon found on exon 6B that is not found on the canonical isoform (ENST00000457361, 526 amino-acids) as in this isoform, exon 6A is spliced to exon 7. This effect results in the premature truncation of the full-length protein and the subsequent loss of the two zinc-finger dimerization domains found on exon 8. We were able to replicate the effect of this asQTL *in vitro* by qPCR of the exon 6A-6B splice-site in LCLs between 6 individuals of the rs3768792[AA] genotype and 6 individuals of the rs3768792[GG] genotype (Supplementary Material, Fig. S6). We detected a log_2_ fold-change of 3.36 (*P *<* *0.0001) in splicing of the exon 6A-6B bridge in risk homozygotes relative to non-risk homozygotes. qPCR of the exon 1-2 bridge (common to all transcripts of *IKZF2*) showed no difference (log_2_ fold-change: 0.07, *P *=* *0.56). Interestingly, we identified an additional asQTL variant (rs2291241) in near-perfect LD with the rs3768792 GWAS variant (*r*^2^:0.99), located 9 bp upstream of exon 6B in truncated isoform ENST00000413091. This second asQTL, located within the polypyrimidine tract in the exon 6A/exon 6B intron, is a highly plausible driving variant and may act through promotion of the described splicing event.

**Figure 5.**
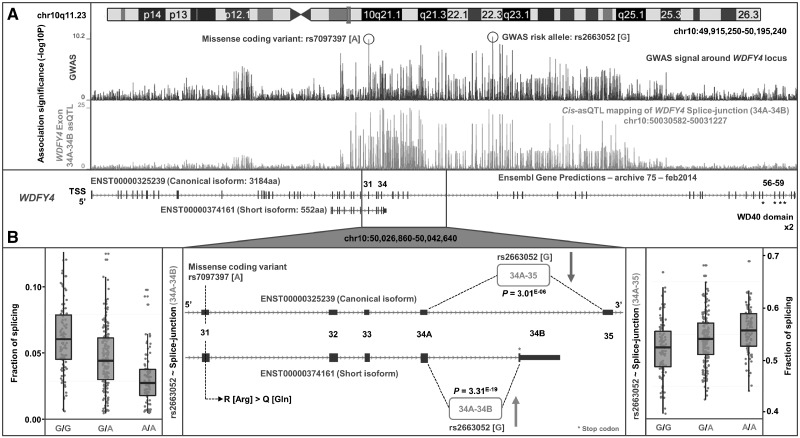
Identification of splicing mechanism in *WDFY4*. (**A**) Our SLE GWAS indicates *WDFY4* as the candidate gene at the chr10q11.23 locus tagged by intronic variant rs2663052, as well as the missense coding variant rs7097397 in exon 31 that is in strong LD. *Cis*-eQTL analysis showed rs2663052 is correlated with upregulation of the exon 34A–34B junction of *WDFY4* (signal is colocalised with GWAS) that is unique to the short isoform (ENST00000374161). This isoform lacks the two enzymatic WD40 domains of the full length isoform (ENST00000325239). (**B**) Two potential functional mechanisms may occur when harbouring the risk haplotype that carries both risk alleles. Firstly, an Arg to Gln amino-acid substitution by rs7097397 in exon 31 that is shared by both the canonical and short isoforms of *WDFY4*, and secondly an upregulation of the short isoform (*P *=* *3.31 × 10^−19^) that lacks functional domains, caused by rs2663052 or correlated variants, with corresponding down-regulation of the full-length isoform (*P *=* *3.01 × 10^−06^).

We also discovered a novel putative SLE-associated splicing mechanism involving *WDFY4* (Fig. 5). Risk variant rs2263052[G] greatly increased the fraction of link-counts between exon 34A and exon 34B (*P *=* *3.3×10^−^^19^) which are unique to the truncated isoform ENST00000374161. This isoform (552 amino-acids) lacks the two WD40 domains found in the full length isoform (ENST00000325239, 3184 amino-acids) that are essential to enzymatic activity (66). There is a consequential decrease in the fraction of link-counts between exon 34A and exon 35 (*P *=* *3.0×10^−^^06^) that are unique to the canonical isoform of *WDFY4*. Interestingly, a known missense variant found in exon 31 of *WDFY4*, rs7097397 (Arg1816Gln), in strong LD (*r*^2^:0.7) with rs2263052, has also been implicated in SLE through GWAS (67); suggesting the risk haplotype may harbour two functional mechanisms influencing *WDFY4* (amino-acid change and upregulation of a shorter isoform) that are both involved in pathogenesis.

### Classification of novel SLE candidate-causal eGenes discovered by RNA-seq

We replicated the known SLE associated eQTLs and eGenes discovered using microarrays, including rs564799 for *IL12A*, rs2736340 for *BLK*, rs9311676 *ABHD6*, and rs2289583 for *ULK*, *CSK*, and *MPI.* Several of these associations have been extensively studied in terms of their role in SLE pathogenesis, for example, rs10028805 for *BANK1* (65) and rs7444 for *UBE2L3* (68). We compared the eGenes detected solely by RNA-Seq against our previous eGene discoveries found in microarray-based eQTL cohorts of primary immune-cell types across an array of conditions (60). We identified a total of 16 eGenes that were not captured within any of the microarray expression cohorts, and have strengthened the evidence that they are likely causal to SLE through their reported function, tissue specific expression, and associations with other complex traits in Table 2. The majority of these novel eGenes showed strongest expression in highly relevant tissues types such as in the spleen and thymus, and also in whole blood and lymphoblastoid cell lines. Similarly, many of these eGenes had previously been reported as candidate genes in other autoimmune traits such as Multiple Sclerosis, Type 1 Diabetes, and Rheumatoid Arthritis.

**Table 2.** Summary of novel candidate-causal eQTLs and eGenes

eQTL	eGene	Gene Function Summary	GTEx Tissue Expression	GWAS Catalog Traits
rs17167273	*TCF7*	Transcriptional activator involved in T-cell lymphocyte differentiation. Necessary for the survival of CD4(+) CD8(+) immature thymocytes.	LCLs, Spleen, Whole Blood	Multiple sclerosis
rs3768792	*IKZF2*	This gene encodes a member of the Ikaros family of zinc-finger proteins. Three members of this protein family (Ikaros, Aiolos and Helios) are hematopoietic-specific transcription factors involved in the regulation of lymphocyte development.	LCLs, Whole Blood	Eosinophil counts
rs10774625	*HECTD4*	E3 ubiquitin-protein ligase which accepts ubiquitin from an E2 ubiquitin-conjugating enzyme in the form of a thioester and then directly transfers the ubiquitin to targeted substrates.	Cerebellum, Cerebellar Hemisphere, Thyroid	Metabolite levels, HDL cholesterol, Esophageal cancer
rs3794060	*RP11-66OL16.2*	Known antisense RNA.	–	–
	NADSYN1*	Nicotinamide adenine dinucleotide (NAD) is a coenzyme in metabolic redox reactions, a precursor for several cell signaling molecules, and a substrate for protein posttranslational modifications.	Spleen, Colon, Terminal Ileum	Vitamin D insufficiency
rs2431697	*MIR146A*	microRNA 146a.	LCLs	–
rs3024505	*FCAMR*	Functions as a receptor for the Fc fragment of IgA and IgM. Binds IgA and IgM with high affinity and mediates their endocytosis. May function in the immune response to microbes mediated by IgA and IgM.	Kidney, Liver, Terminal Ileum	–
	*IL10*	Inhibits the synthesis of a number of cytokines, including IFN-gamma, IL-2, IL-3, TNF and GM-CSF produced by activated macrophages and by helper T-cells.	LCLs, Spleen, Whole Blood	Inflammatory bowel disease, Ulcerative Colitis, Crohn's disease
	*IL24*	This gene encodes a member of the IL10 family of cytokines. Overexpression of this gene leads to elevated expression of several GADD family genes, which correlates with the induction of apoptosis.	Spleen, LCLs, Whole Blood	Inflammatory bowel disease, Alzheimer's disease
rs2476601	*DCLRE1B*	5-3 exonuclease that plays a central role in telomere maintenance and protection during S-phase. Participates in the protection of telomeres against non-homologous end-joining (NHEJ)-mediated repair.	LCLs, Fibroblasts, Cerebellar Hemisphere	Rheumatoid arthritis, Type 1 diabetes autoantibodies
	*MAGI3*	Cooperates with PTEN to modulate the kinase activity of AKT1. Its interaction with PTPRB and tyrosine phosphorylated proteins suggests that it may link receptor tyrosine phosphatase with its substrates at the plasma membrane.	Thyroid, Cerebellar Hemisphere, Lung	Rheumatoid arthritis, Type 1 diabetes autoantibodies
rs2663052	WDFY4*	WDFY family member 4.	LCLs, Spleen, Whole Blood	Rheumatoid arthritis, Stroke
rs2736340	*RP11-148O21.2*	Known antisense RNA.	Spleen, LCLs, Terminal Ileum	–
	*RP11-148O21.4*	Known antisense RNA.	Spleen, LCLs, Terminal Ileum	–
rs2396545	*ANO9*	Has calcium-dependent phospholipid scramblase activity; scrambles phosphatidylserine, phosphatidylcholine and galactosylceramide.	Terminal Ileum, Colon, Skin	–
rs2289583	*UBE2Q2*	Accepts ubiquitin from the E1 complex and catalyzes its covalent attachment to other proteins. In vitro catalyzes Lys-48-linked polyubiquitination.	Colon, Esophagus, Bladder	Chronic kidney disease, Urate levels

Candidate-causal eGenes detected by RNA-Seq that have not been documented in previous microarray analyses in LCLs and other primary immune-cell types. Gene Function Summary is taken from a combination of Entrez gene and UNIProt annotation. GTEx tissue expression reports the top three tissue types where the gene is most expressed. The top three traits from GWASs where the gene is reported as the candidate gene is also given.

* Found with microarray as well, but RNA-Seq allows for detection of novel alternative-splicing mechanism.

## Discussion

Detailed characterization of the functional effects of human regulatory genetic variation associated with complex-disease is paramount to our understanding of molecular aetiology and poised to make significant contributions to translational medicine (69). Use of eQTL mapping studies to interpret GWAS findings have proved fundamental in our progression towards this goal—through prioritization of candidate genes, refinement of causal variants, and illumination of mechanistic relationships between disease-associated genetic variants and gene expression (69,70). However, there is often a disparity between disease-associated genetic variation and phenotypic alteration, which historically may be due to the use of microarray-based technologies to profile genome-wide gene expression. With the advent of RNA-Seq, we can achieve more accurate quantification of the mRNA output of genes, individual exons, and isoform abundance, as well as unannotated and non-coding transcripts. Detection of splicing variants at susceptibility loci using RNA-Seq has the potential to uncover the role of specific isoforms implicated in disease risk, which are likely to have remained concealed by microarray, as a largely independent subset of variants control alternative splicing of isoforms compared to overall gene abundance (35).

Our motivation for this study was to directly compare the ability of microarray and RNA-Seq profiled at various resolutions to detect candidate-causal eQTLs and their associated eGenes from GWAS, and assess their effectiveness in explaining potential regulatory mechanisms. We performed c*is*-eQTL association analysis combined with conditional and colocalisation testing for 39 SLE susceptibility loci against expression quantifications from both microarray and RNA-Seq experiments from the TwinsUK (gene- and exon-level) and Geuvadis (splice-junction-level) cohort (summarized in Fig. 1). Our investigation explicitly shows that RNA-Seq is more powerful than microarrays for the identification of candidate-causal eQTLs and their accompanying eGenes. Exon-level RNA-Seq yielded the greatest frequency of candidate-causal associations, and for this reason, we believe exon-level quantification should be used as the primary quantification type when performing integrative GWAS-eQTL analysis. To maximise the likelihood of capturing the true function of regulatory variants however, exon-level quantification should be run in parallel with gene-level and splice-junction level quantifications. Excluding one or more levels of analysis will result in false-negative candidate-causal eQTLs. For example, the novel eGene, *TCF7* was detected at gene-level only. *TCF7* has been implicated in Type 1 Diabetes risk (71), but there is only weak LD (*r*^2 ^<^ ^0.4) between the reported missense variant and SLE risk SNP rs7726414 (19 kb upstream of *TCF7*) or any protein-coding variants of *TCF7*, suggesting in SLE the causal mechanism may be dysregulation of expression of *TCF7* rather than a missense change. The literature suggests *TCF7* plays a role in B cell and T cell development and knockdown results in the impaired gene expression regulation of CD34+ cells (72). At numerous loci, there was an exon-level effect not observed at the gene-level, suggesting subtle exon-level effects influencing perhaps just a single exon will be masked by whole gene-level quantification. Recent work has suggested ‘union-exon’ based approaches to quantify overall gene expression (as used in TwinsUK) allow reads to be assigned with greater confidence, but significantly underestimates actual expression output and is prone to variability when the number of isoforms of the gene increases or when shorter isoforms are more highly expressed (47). Transcript-based approaches which rely on expectation maximization algorithms to distribute reads among gene isoforms should be considered when attempting to quantify whole gene expression as these effects are diminished, although high genomic overlap of isoform structures complicates these models (47). We have shown that greater biological insight is gained from using exon-level quantification (created by merging all overlapping exonic portions of a gene into non-redundant units) than from whole gene expression estimates (union-exon approach). Using the unique exons of the transcript annotation, one can isolate the specific isoforms(s) regulated by the eQTL (Fig. 2); though this is not always possible due to considerable overlap. We also stress there must be stringent colocalisation procedures when performing GWAS-eQTL integration strategies at exon-level as this quantification type led to the greatest proportion of non-candidate-causal associations.

Exon-level quantification largely increased the average ratio of candidate-causal eGenes to eQTLs compared with gene-level and microarray. The ability of RNA-Seq exon-level analysis to identify multiple target eGenes for an individual eQTL is supported by observations from capture Hi-C experiments (73,74). It has been shown that chromatin interactions can control transcription in *cis* in a largely sequence-specific manner, thus it is likely that some GWAS variants may functionally act through the disruption of chromatin dynamics resulting in perturbation of expression of multiple genes (73,75,76). Specific instances of this type of effect have been observed in colorectal cancer risk loci where for example the risk SNP rs6983267 within 8q24 disrupts a chromatin regulatory network involving interactions between three genes *CCAT2*, *CCAT1* and *MYC* (73). Our results support this notion of multiple perturbed genes at a single susceptibility locus. rs3024505 for example was found to be associated with three plausible candidate-causal eGenes: *IL10*, *IL24*, and *FCAMR* (located 1 kb, 130 kb, and 191 kb away from rs3024505 respectively). These chromatin capture data also support the argument of using exon-level quantification and extending the traditional *cis*-eQTL distance (typically +/-0.25–1Mb) to encompass the length of the TAD which holds the disease-association signal (77). *Trans*-eQTL analyses should also be attempted at exon-level resolution. Interestingly with exon-level RNA-Seq, we were able to find examples of a single eQTL able to cause dysregulation of multiple molecular genetic mechanisms. For example, rs3794060 led to a gene-level effect of *DHCR7*, a potential splicing effect of *NADSYN1* specific to a single isoform, and dysregulation of a non-coding RNA, *RP11-660L16.2*. Similarly, rs2263052 increased the fraction of splicing of a truncated isoform of *WDFY4* and is also in strong LD with a missense variant rs7097397 (Arg1816Gln). Our data support and further the concept of susceptibility haplotypes carrying multiple functional effects.

Exon-level and splice-junction level analyses also enabled not only the discovery of novel candidate SLE eGenes (Table 2), but also potential splicing-mechanisms which would have been missed by microarrays and even gene-level quantification. We replicated by qPCR a splicing mechanism discovered by asQTL analysis within *IKZF2* caused by tagging variant rs3768792. *IKZF2* is a transcription factor thought to play a key role in T-reg stabilisation in the presence of inflammatory responses (78). Other members of this gene family, *IKZF1* and *IKZF3*, are also associated with SLE (60). Since the Ikaros transcription factor family primarily regulate gene expression through homo-/hetero-dimerization and DNA binding/protein-protein interactions, the rs3768792[G] dependent asQTL effect on exon 6A to 6B resulting in less functional *IKZF2* could be highly deleterious (Fig. 5). *IKZF2* is known to regulate T-reg-associated genes, including *IL-2* and *FoxP3* (79,80), therefore we hypothesize that upregulation of the shorter isoform of *IKZF2* caused by rs3768792[G], which lacks the dimerization domain, reduces translocation of the protein into the nucleus and regulation of T-reg specific target genes. A similar mechanism was found at candidate-causal eGene *NADSYN1*. Using exon-level quantification, we were able to pinpoint the specific transcript of *NADSYN1* (ENST00000528509) that drives the gene-level association (Fig. 2). Interestingly, this transcript is translated into a 294 amino acid long protein (canonical transcript 706 amino acids). The shorter protein lacks the NAD (+) Synthetase domain (located in positions 339-602aa, Pfam: PF02540) implicating loss of this domain as a potential causal mechanism. Although no autoimmune phenotype has been described at this locus, rs3794060[C] is correlated with altered circulating 25-hydroxy vitamin D concentrations (81). The ability to resolve a potential functional mechanism down to a single transcript greatly facilities the design and implementation of targeted follow-up studies which aim to assess the phenotypic consequence of disease-associated variant(s). Such experiments could include site directed mutagenesis to introduce splice-sites and over-express target isoforms. Similarly, the CRISPR/Cas9 system for targeted genome editing presents an exciting opportunity for eQTL targeted follow-up studies *in vivo* and the investigation into the transcriptomic consequence of specific regulatory variants. Integration with epigenetic data (promoter methylation, histone modification and expression of non-coding RNA) will also allow insight into potential regulatory mechanisms and fine-mapping of regulatory variants.

We stress that to better understand disease aetiology, large RNA-Seq based eQTL cohorts should be generated across a multitude of disease-relevant cell-types and conditions. Though LCLs are a good surrogate model for primary B-cells, the effect of EBV transformation is likely to disrupt their underlying epigenetic and transcriptomic background. The percentage of asQTLs in LCLs will exhibit significantly less replication in primary cell types due to cell-type variability in the genetic control of isoform usage (33). We believe to better understand the implications of the genetic control of gene expression in genomic medicine, a gold standard of eQTL mapping strategies using an explicit set of quantification types (gene-, exon-, splice-junction, isoform), gene-annotations, and analytical pipelines, should be adopted.

In summary, we have demonstrated the effectiveness of eQTL analysis using RNA-Seq, primarily and exon-level, by increasing the number of candidate genes derived from an SLE GWAS. We have shown that the power of RNA-Seq for eQTL annotation lies not only the assessment of the variants regulating the expression of candidate genes but also in the discovery of specific molecular aberrations.

## Materials and Methods

### Selection of SLE-associated SNPs

SLE associated SNPs were taken from our recent publication (60). The study comprised a primary GWAS, with validation through meta-analysis and replication study in an external cohort (7,219 cases, 15,991 controls in total). Independently-associated susceptibility loci taken forward for this investigation were those that passed either genome-wide significance (*P*< 5×10^−^^08^) in the primary GWAS or meta-analysis and/or those that reached significance in the replication study (q < 0.01). We defined the ‘GWAS SNP’ at each locus as either being the SNP with the lowest *P*-value post meta-analysis or the SNP with the greatest evidence of a missense effect as defined by a Bayes Factor. We omitted non-autosomal associations and those within the Major Histocompatibility Complex (MHC), and SNPs with a MAF <0.05. In total, 39 GWAS SNPs were taken forward (Supplementary Material, Table S1).

### TwinsUK eQTL analysis

Expression profiling by microarray (9) and RNA-Seq (39) of individuals from the UK Adult Twin Registry (TwinsUK) was carried out in two separate studies on the MuTHER (Multiple Tissue Human Expression Resource) cohort (Table 1). The MuTHER cohort is composed of 856 healthy female individuals of European descent aged between 37-85 years. We considered expression quantification data from both resting LCLs and whole blood. Profiling by microarray was performed using the Illumina Human HT-12 V3 BeadChips. For RNA-Seq, samples were sequenced using the Illumina HiSeq2000 and the 49-Bp paired-end reads mapped with BWA v0.5.9 to the GRCh37 reference genome. Exons (‘meta-exons’ created by merging all overlapping exonic portions of a gene into non-redundant units) were quantified using read-counts against the GENCODE v10 annotation; with gene quantification defined as the sum of all exon quantifications belonging to the same gene (union-exon). Full quality control and normalization procedures are described in the respective articles. Data from each of the TwinsUK eQTL studies were provided in different formats. In each instance it was necessary to generate summary eQTL statistics per GWAS SNP (SNP, expression-unit, β, standard error of β, and *P*-value of association) for integration analysis. Per quantification type (microarray, RNA-Seq gene-level, and exon-level), each GWAS SNP was subject to *cis*-eQTL analysis against all expression-units within +/-1Mb using no *P*-value threshold. If the GWAS SNP was not found in an eQTL dataset, the most highly correlated, closest tag SNP with *r*^2 ^≥^ ^0.7, common to all datasets, was used as proxy. Adjustment for multiple testing of eQTL results per quantification type were undertaken using FDR with q <0.05 deemed significant.

#### Microarray cis-eQTL mapping

We used the Genevar (GENe Expression VARiation) portal to generate summary-level eQTL results (53). We ran the association between normalized expression data of the 777 available individuals and each GWAS SNP implementing the external algorithm option (two-step mixed model–based score test). In total 768 probes (559) genes, were tested for association.

#### RNA-seq (gene-level) cis-eQTL mapping

RNA-Seq gene-level quantification was provided as residualized read-counts (effect of family structure and other covariates regressed out). We had full genetic data for 683 individuals and performed the analysis of each GWAS SNP against the transformed residuals using the linear-model function within the MatrixeQTL R package (82). 520 genes were tested against in *cis*.

#### RNA-seq (exon-level) cis-eQTL mapping

*P*-values from the association of all SNPs against exon-level quantifications for 765 individuals using linear-regression were provided. We generated the t-statistic using the lower-tail quantile function t-distribution function in R with 763 degrees of freedom. The standard error and β were derived from the t-statistic. We then extracted the summary *cis*-eQTL results for each GWAS SNP. 4,786 exons, corresponding to 716 genes were taken forward for association analysis.

### Candidate-causal *cis*-eQTL classification

#### Conditional analysis

We used the COJO (conditional and joint genome-wide association analysis) function of the GCTA (Genome-wide Complex Trait Analysis) application to determine whether the GWAS SNP had an independent effect on expression from that of the best *cis*-eQTL (58). For each significant association (q < 0.05), we re-performed the analysis using all SNPs within +/-1Mb of the expression-unit in hand. We used the available genotype information of the 683 TwinsUK individuals to extract allele coding along with the MAF, and integrated this with the eQTL summary data. We discarded SNPs with: MAF < 0.05, imputation call-rates < 0.8, and HWE *P *<* *1×10^−^^04^. We used these individuals as the reference panel to calculate local pairwise linkage disequilibrium (LD) between variants. Per significant association, all *cis*-eQTLs were conditioned on by the best *cis*-eQTL. We then extracted the conditional *P*-value of the GWAS SNP and considered associations to be independent to the best *cis*-eQTL if P_cond _<_ _0.05.

#### Colocalisation analysis

We employed the ′coloc′ Bayesian statistical method using summary data implemented in R to test for colocalisation between eQTL and disease causal variants derived from the GWAS (59). The method makes the assumption of there being a single causal variant for each trait (disease association and gene-expression from two separate studies) per locus and calculates the posterior probabilities under five different causal variant hypotheses: association with neither trait (H0), association with one trait but not the other (H1, H2), association with both traits but from independent signals, and association with both traits with a shared causal signal (H4). We extracted the necessary SNP statistics for the disease-associated regions from our own GWAS and applied the same SNP filters used in the conditional analysis. We tested for colocalisation between the GWAS summary data and eQTL data for each significant association within a +/-1Mb window of the GWAS SNP. We assigned the prior probabilities, p1 and p2 (SNP is associated with GWAS and gene expression respectively), as 1 x 10^−^^04^ i.e. 1 in 10,000 SNPs are causal to either trait, with p12 (SNP is associated with both traits) as 1×10^−^^06^ or 1 in 100 SNPs associated with one trait are also associated with the other. For each eQTL association colocalisation test, if the posterior probability PP3 (two distinct causal variants, one for each trait) is greater than PP4 (single causal variant common to both traits), then greater posterior support is given to the hypothesis that independent causal variants exist in both traits and thus the eQTL is unlikely to be attributed to SLE genetic association.

### Definition of candidate-causal eQTL and eGene

We defined a GWAS SNP as an SLE candidate-causal eQTL if it met the following criteria: significant post-multiple testing adjustment (q < 0.05), not independent to the best eQTL from conditional analysis (*P*_cond _> 0.05), and supporting evidence of a shared causal variant between gene expression and the primary GWAS signal based on colocalisation (PP3 < PP4). The gene whose expression is modulated by the candidate-causal eQTL is defined as an SLE candidate-causal eGene. As the individuals used for eQTL analysis per quantification type were selected from the same pool of 856, but sample sizes differed – we performed power calculations to estimate the differences in power between groups. We show this is a very high powered study for both RNA-Seq and Microarray data when the effect size (R2) is 0.05 or above. The difference in power for weak effects is not great between quantification types (Supplementary Material, Table S9).

### Validation of LCL candidate-causal eQTLs in whole blood

*Cis*-eQTL summary data from whole blood at RNA-Seq exon-level were made available for 384 individuals of the 856 TwinsUK cohort individuals (Table 1). Expression profiling and genotyping were identical to that as described for LCLs. We applied the same methodology to this dataset to generate full eQTL summary statistics, perform conditional and colocalisation analysis, and classify SLE candidate-causal eQTLs and associated eGenes. In total, 3,793 exons were tested against, corresponding to 654 genes.

### Geuvadis *cis*-asQTL analysis

We investigated SLE disease-associated alternative splicing QTLs (asQTLs) using European samples from the raw alignment files of the Geuvadis (35) 1000 Genomes RNA-Seq project profiled in LCLs (Table 1). Genotype data and read-alignments were downloaded from ArrayExpress for the 373 Europeans (comprising 91 CEU, 95 FIN, 94 GBR, and 93 TSI). We performed PCA on chromosome 20 using the R/Bioconductor package SNPRelate (83) and decided to include the first three principal components as covariates in the eQTL model as well as the binary imputation status (mixture of Phase 1 and Phase 2 imputed individuals). We removed SNPs with MAF < 0.05, imputation call-rates < 0.8, and HWE *P *<* *1×10^−^^04^. We removed non-uniquely mapped, non-properly paired reads, and reads with more than eight mismatches for read and mate using SAMTools (84). We used the Altrans (85) method against GENCODE v10 to generate relative quantifications (link-counts) of splicing events; which in brief, utilizes split and paired-end reads to count links between exon-boundaries, which themselves are created by flattening the annotation into unique non-redundant exon-groups. Following PCA of the link-counts, we decided to normalize all link-counts with the first 10 principle components then removed exon-boundaries with zero links in more than 10% of individuals. Link-counts were converted to link-fractions (coverage of the link over the sum of the coverage of all the links that the first exon makes) and merged in both 5′-3′ and 3′-5′ directions. Per GWAS SNP we performed *cis*-eQTL analysis against the normalized link-fractions in MatrixeQTL with a linear-model (82). 33,039 link-fractions were tested against corresponding to 817 genes in total. After FDR multiple-testing adjustment we considered associations with q < 0.05 as significant. As full genetic and expression data were available, we decided to use the Regulatory Trait Concordance (RTC) method to assess the likelihood of a shared functional variant between the GWAS SNP and the asQTL signal (51). For each significant asQTL association we extracted the residuals of the linear-regression of the best *cis*-eQTL against normalized link-fractions and re-performed the analysis using all SNPs within the defined hotspot interval against this pseudo-phenotype. The RTC score was defined as (N_SNPs_-Rank_GWAS SNP_)/N_SNPs_ where N_SNPs_ is the number of SNPs in the interval, and Rank_GWAS SNP_ is the rank of the GWAS SNP association *P*-value against all other SNPs in the interval. We classified an SLE candidate-causal asQTL as a GWAS SNP with a significant association (q < 0.05) with link-fraction quantification and an RTC score > 0.9.

### qPCR validation of asQTL

Twelve human lymphoblastoid cell lines (6 x rs3768792 [AA], 6 x rs3768792 [GG] were obtained from Coriell Biorepository and cultured at 5% CO_2_ and 37^°^C in RPMI 1640 medium supplemented with 2 mM L-glutamine, 15% fetal bovine serum, 100 unit/ml penicillin and 100 µg/ml streptomycin. Total RNA was extracted using the RNeasy Micro Kit (Qiagen) and cDNA synthesized with the cDNA Synthesis Kit (Thermo Scientific). Primers were purchased from Sigma and reactions performed using the Applied Biosystems 7500. *IKZF2* exon 6A-exon 6B splice-site, UPL #3, forward primer: TGGAATC AGCTCT AAC TATTGGTG, reverse primer: ACGCTGCCACAACTATCTCC. Relative mRNA and fold change was calculated in relation to GAPDH expression using the ΔΔCt method.

## Supplementary Material

Supplementary Material is available at HMG online.

## Supplementary Material

Supplementary Data
