# Supplementary material for: Mapping eQTLs with RNA-seq reveals novel susceptibility genes, non-coding RNAs and alternative-splicing events in systemic lupus erythematosus
Source: Hum Mol Genet. 2017 Jan 5;26(5):1003–17. doi: 10.1093/hmg/ddw417 (PMC5409091; doi:10.1093/hmg/ddw417)
Supplement: Supplementary Data [file ddw417_Supp.docx]

**Mapping eQTLs with RNA-Seq Reveals Novel Susceptibility Genes, Non-Coding RNAs, and Alternative-Splicing Events in Systemic Lupus Erythematosus**

Christopher A. Odhams^1^, Andrea Cortini^1^, Lingyan Chen^1^, Amy L. Roberts^1^, Ana Viñuela^2^, Alfonso Buil^3-5^, Kerrin S. Small^2^, Emmanouil T. Dermitzakis^3-5^, David L. Morris^1^, Timothy J. Vyse^1, 6^, Deborah S. Cunninghame Graham^1, *^

^1^Division of Genetics and Molecular Medicine, King’s College London, London, UK. ^2^Department of Twin Research, King’s College London, London, UK. ^3^Department of Genetic Medicine and Development, University of Geneva Medical School, Geneva, Switzerland. ^4^Institute of Genetics and Genomics in Geneva, University of Geneva, Geneva, Switzerland. ^5^Swiss Institute of Bioinformatics, Geneva, Switzerland. ^6^Division of Immunology, Infection and Inflammatory Disease, King’s College London, London, UK.

^*^To whom correspondence should be addressed

Address: Immunogenetics group, Department of Medical and Molecular Genetics, Division of Genetics and Molecular Medicine, King's College London School of Medicine, 7th Floor, Tower Wing, Guy's Hospital, Great Maze Pond, London, SE1 9RT

Telephone: +44 (0)207 848 8504

Fax: +44 (0)207 188 2585Email: [deborah.cunninghame-graham@kcl.ac.uk](mailto:deborah.cunninghame-graham@kcl.ac.uk)

| **Supplementary Material, Table S1**  Independent allelic associations at SLE susceptibility loci following meta-analysis with replication study | | | | | | |
| --- | --- | --- | --- | --- | --- | --- |
| **GWAS SNP**  **(Tag SNP)** | **GWAS SNP**  **Position (hg19)** | **Variant**  **Alleles** | **Odds Ratio (CI)** | ***P*-value** | **GENCODE v10**  **Annotation** |  |
| rs2476601 | 1:114377568 | G/**A** | 1.43 (1.34-1.53) | 1.10E-28 | *PTPN22* Missense |  |
| rs1801274 | 1:161479745 | **G**/A | 1.16 (1.11-1.21) | 1.04E-12 | *FCGR2A* Missense |  |
| rs10912578 (rs844663) | 1:173251856 | G/**A** | 1.27 (1.22-1.33) | 4.16E-19 | *LOC100506023* Intronic |  |
| rs10753074 (rs1935325) | 1:173346343 | **T**/C | 1.21 (1.15-1.26) | 5.82E-12 | *LOC100506023* Intronic |  |
| rs17849501 | 1:183542323 | C/**T** | 2.10 (1.95-2.26) | 3.45E-88 | *NCF2* Synonymous |  |
| rs3024505 | 1:206939904 | G/**A** | 1.17 (1.11-1.24) | 4.64E-09 | 1kb 3′ of *IL10* |  |
| rs9782955 | 1:236039877 | **C**/T | 1.16 (1.11-1.22) | 1.25E-09 | *LYST* Intronic |  |
| rs268134 | 2:65608363 | **G**/A | 1.21 (1.15-1.27) | 1.14E-10 | *SPRED* Intronic |  |
| rs2111485 | 2:163110536 | **G**/A | 1.15 (1.11-1.2) | 1.27E-11 | 8.9kb 5′ of *FAP* |  |
| rs11889341 | 2:191943742 | C/**T** | 1.70 (1.64-1.75) | 2.48E-75 | *STAT4* Intronic |  |
| rs6736175 (rs16833249) | 2:191946322 | T/**C** | 1.24 (1.19-1.29) | 9.17E-17 | *STAT4* Intronic |  |
| rs3768792 | 2:213871709 | A/**G** | 1.24 (1.17-1.31) | 1.21E-13 | *IKZF2* 3′-UTR |  |
| rs9311676 (rs11714389) | 3:58470351 | **C**/T | 1.17 (1.13-1.22) | 3.06E-14 | *LOC101929223* NC |  |
| rs564799 | 3:159728987 | **C**/T | 1.14 (1.09-1.18) | 1.54E-09 | *IL12A-AS1* Intronic |  |
| rs10028805 | 4:102737250 | **G**/A | 1.20 (1.15-1.25) | 4.31E-17 | *BANK1* Intronic |  |
| rs7726414 (rs17167273) | 5:133431834 | C**/T** | 1.45 (1.32-1.58) | 4.44E-16 | 19kb 5′ of *TCF7* |  |
| rs10036748 | 5:150458146 | C/**T** | 1.38 (1.32-1.45) | 1.27E-45 | *TNIP1* Intronic |  |
| rs2431697 | 5:159879978 | **T**/C | 1.26 (1.21-1.31) | 8.01E-28 | 15kb 5′ of *hsa-mir-146a* |  |
| rs6568431 | 6:106588806 | C/**A** | 1.21 (1.15-1.27) | 5.04E-14 | 31kb 3′ of *PRDM1* |  |
| rs6932056 | 6:138242437 | T/**C** | 1.83 (1.65-2.02) | 1.97E-31 | 22kb 3′ of *RP11-10J5.1* |  |
| rs849142 | 7:28185891 | **T**/C | 1.14 (1.1-1.19) | 8.61E-11 | *JAZF1* Intronic |  |
| rs4917014 | 7:50305863 | **T**/G | 1.18 (1.13-1.24) | 6.39E-14 | 8.5kb 3′ of *AC020743.4* |  |
| rs3757387 (rs4728142) | 7:128576086 | T/**C** | 1.45 (1.4-1.5) | 1.14E-48 | 1.6kb 5′ of *IRF5* |  |
| rs35000415 (rs10488631) | 7:128585616 | C/**T** | 1.83 (1.76-1.9) | 1.20E-60 | *IRF5* Intronic |  |
| rs2736340 | 8:11343973 | C/**T** | 1.29 (1.22-1.37) | 6.28E-20 | 7.5kb 5′ of *BLK* |  |
| rs2663052 | 10:50069395 | **G**/A | 1.16 (1.1-1.22) | 5.25E-09 | *WDFY4* Intronic |  |
| rs4948496 | 10:63805617 | T/**C** | 1.14 (1.1-1.19) | 1.04E-10 | *ARID5B* Intronic |  |
| rs12802200 (rs2396545) | 11:566936 | **C**/A | 1.23 (1.15-1.31) | 8.81E-10 | *MIR210HG* NC |  |
| rs2732549 | 11:35088399 | **A**/G | 1.24 (1.19-1.29) | 1.20E-23 | 46kb 3′ of *PDHX* |  |
| rs3794060 | 11:71187679 | T/**C** | 1.23 (1.18-1.29) | 1.32E-20 | *NADSYN1* Intronic |  |
| rs7941765 (rs6590343) | 11:128499000 | **C/**T | 1.14 (1.1-1.19) | 1.35E-10 | 547bp 3′ of *RP11-744N12.3* |  |
| rs10774625 | 12:111910219 | **A**/G | 1.13 (1.08 - 1.18) | 4.09E-09 | *ATXN2* Intronic |  |
| rs1059312 | 12:129278864 | A/**G** | 1.17 (1.12-1.21) | 1.48E-13 | *SLC15A4* Synonymous |  |
| rs4902562 | 14:68731458 | G/**A** | 1.14 (1.09-1.19) | 6.15E-10 | *RAD51B* Intronic |  |
| rs2289583 | 15:75311036 | C/**A** | 1.19 (1.14-1.24) | 6.22E-15 | *SCAMP5* Intronic |  |
| rs9652601 | 16:11174365 | **G**/A | 1.21 (1.15-1.26) | 7.42E-17 | *CLEC16A* Intronic |  |
| rs34572943 (rs9936831) | 16:31272353 | G/**A** | 1.71 (1.61-1.81) | 3.39E-76 | *ITGAM* Intronic |  |
| rs11644034 | 16:85972612 | **G**/A | 1.25 (1.19-1.32) | 9.58E-18 | 16kb 3′ of *IRF8* |  |
| rs2286672 | 17:4712617 | C/**T** | 1.25 (1.16-1.35) | 2.93E-09 | *PLD2* Missense |  |
| rs2941509 | 17:37921194 | C/**T** | 1.35 (1.22-1.49) | 7.98E-09 | *IKZF3* 3′-UTR |  |
| rs2304256 | 19:10475652 | **C**/A | 1.24 (1.17-1.31) | 3.50E-13 | *TYK2* Missense |  |
| rs7444 | 22:21976934 | T/**C** | 1.27 (1.21-1.33) | 1.84E-22 | *UBE2L3* 3′-UTR |  |
| SLE GWAS SNPs taken forward for *cis*-eQTL association analysis. Results from post-replication meta-analysis as described (57). SNP with the lowest P-value post meta-analysis or the SNP with the greatest evidence of a missense effect as defined by a Bayes Factor reported. Autosomal, non-MHC SNPs with MAF > 0.05 included in analysis only (39 total). Risk alleles are highlighted in bold type – minor allele on right. Functional annotation from HaploReg v4.0 using GENCODE genes v10. | | | | | | |

| **Supplementary Material, Table S2** All significant eQTLs (q < 0.05) and associated eGenes detected at microarray (probe-level) with conditional and colocalisation results | | | | | | | | | | | | | | | | | | | | |
| --- | --- | --- | --- | --- | --- | --- | --- | --- | --- | --- | --- | --- | --- | --- | --- | --- | --- | --- | --- | --- |
| **Risk**  **Locus** | **GWAS SNP** | **Coordinate (hg19)** | **eGene** | **Probe ID** | **β** | **Std Err** | **P-Value** | **FDR (q)** | **best cis-eQTL** | **β** | **Std Err** | **P-Value** | **β _cond_** | **Std Err _cond_** | **P-Value _cond_** | **Independent  to best** | **PP3** | **PP4** | **Shared Causal Variant** | **Candidate Causal eQTL/eGene** |
| 1p13.2 | rs2476601 | 1:114377568 | *BCL2L15* | ILMN_1655722 | -0.075 | 0.022 | 8.26E-04 | 2.73E-02 | rs3761936 | -0.126 | 0.018 | 4.48E-12 | 0.015 | 0.018 | 3.95E-01 | NO | 0.014 | 0.018 | YES | YES |
| 1q42.3 | rs9782955 | 1:236039877 | *LYST* | ILMN_1663131 | -0.033 | 0.010 | 1.60E-03 | 4.62E-02 | rs4660123 | 0.066 | 0.010 | 3.36E-11 | -0.011 | 0.010 | 2.54E-01 | NO | 0.021 | 0.017 | NO | NO |
| 2q34 | rs3768792 | 2:213871709 | *IKZF2* | ILMN_1807425 | -0.104 | 0.031 | 6.40E-04 | 2.33E-02 | rs12993121 | 0.195 | 0.022 | 7.93E-20 | -0.040 | 0.030 | 1.77E-01 | NO | 0.051 | 0.045 | NO | NO |
| 2p14 | rs268134 | 2:65608363 | *SPRED2* | ILMN_1791232 | -0.127 | 0.019 | 3.41E-11 | 2.36E-09 | rs10193337 | 0.396 | 0.036 | 1.28E-28 | -0.055 | 0.018 | 2.64E-03 | YES | 0.050 | 0.037 | NO | NO |
| 3q25.33 | rs564799 | 3:159728987 | *IL12A* | ILMN_1671353 | 0.113 | 0.016 | 4.91E-13 | 4.86E-11 | rs4679867 | -0.116 | 0.016 | 3.07E-13 | 0.009 | 0.007 | 1.74E-01 | NO | 0.034 | 0.039 | YES | YES |
| 3p14.3 | rs11714389 | 3:58447299 | *ABHD6* | ILMN_1706344 | -0.130 | 0.018 | 1.41E-13 | 1.63E-11 | rs4681827 | -0.177 | 0.018 | 2.50E-23 | -0.019 | 0.014 | 1.63E-01 | NO | 0.025 | 0.049 | YES | YES |
|  |  |  | *PDHB* | ILMN_1739274 | -0.055 | 0.013 | 2.55E-05 | 1.26E-03 | rs7633553 | 0.074 | 0.014 | 7.03E-08 | -0.008 | 0.017 | 6.36E-01 | NO | 0.030 | 0.035 | YES | YES |
| 4q24 | rs10028805 | 4:10273725 | *BANK1* | ILMN_1661646 | 0.195 | 0.027 | 7.98E-13 | 6.91E-11 | rs7656409 | 0.223 | 0.027 | 2.67E-17 | 0.012 | 0.016 | 4.46E-01 | NO | 0.037 | 0.059 | YES | YES |
| 5q31.1 | rs17167273 | 5:133459347 | *TCF7* | ILMN_1676470 | -0.100 | 0.031 | 1.00E-03 | 3.15E-02 | rs729800 | 0.065 | 0.016 | 2.93E-05 | -0.059 | 0.029 | 4.44E-02 | YES | 0.032 | 0.036 | YES | NO |
| 5q33.3 | rs2431697 | 5:159879978 | *SLU7* | ILMN_1685369 | -0.028 | 0.009 | 1.10E-03 | 3.31E-02 | rs2910191 | -0.067 | 0.013 | 1.25E-07 | -0.024 | 0.009 | 5.90E-03 | YES | 0.047 | 0.033 | NO | NO |
| 7q32.1 | rs4728142 | 7:128573967 | *IRF5* | ILMN_1670576 | 0.645 | 0.052 | 1.03E-35 | 3.57E-33 | rs12155080 | 1.115 | 0.052 | 2.29E-102 | 0.243 | 0.051 | 1.57E-06 | YES | 0.949 | 0.003 | NO | NO |
|  |  |  | *IRF5* | ILMN_2312606 | 0.696 | 0.057 | 1.39E-34 | 3.21E-32 | rs12155080 | 1.214 | 0.057 | 8.36E-101 | 0.250 | 0.055 | 5.72E-06 | YES | 0.978 | 0.001 | NO | NO |
|  |  |  | *TNPO3* | ILMN_1683811 | 0.114 | 0.022 | 2.32E-07 | 1.34E-05 | rs11768572 | -0.224 | 0.023 | 2.27E-22 | 0.021 | 0.020 | 2.96E-01 | NO | 0.058 | 0.039 | NO | NO |
| 7q32.1 | rs10488631 | 7:128594183 | *IRF5* | ILMN_1670576 | 0.294 | 0.077 | 1.27E-04 | 5.18E-03 | rs12155080 | 1.115 | 0.052 | 2.29E-102 | 0.044 | 0.075 | 5.56E-01 | NO | 0.949 | 0.003 | NO | NO |
| 8p23.1 | rs2736340 | 8:11343973 | *BLK* | ILMN_1668277 | -0.407 | 0.040 | 7.69E-25 | 1.07E-22 | rs4840568 | 0.438 | 0.041 | 3.55E-27 | -0.034 | 0.020 | 8.33E-02 | NO | 0.043 | 0.204 | YES | YES |
|  |  |  | *FAM167A* | ILMN_1687213 | 0.412 | 0.030 | 1.48E-43 | 1.03E-40 | rs13277113 | -0.417 | 0.030 | 6.15E-44 | 0.000 | 0.000 | 0.00E+00 | NO | 0.045 | 0.277 | YES | YES |
| 10q11.23 | rs2663052 | 10:50069395 | *WDFY4* | ILMN_1664488 | 0.048 | 0.012 | 3.07E-05 | 1.42E-03 | rs2457214 | 0.097 | 0.013 | 4.58E-15 | -0.004 | 0.009 | 7.07E-01 | NO | 0.048 | 0.033 | NO | NO |
| 15q24.2 | rs2289583 | 15:75311036 | *CSK* | ILMN_1754121 | -0.059 | 0.012 | 9.56E-07 | 5.10E-05 | rs1378940 | 0.085 | 0.012 | 1.27E-13 | -0.012 | 0.010 | 2.40E-01 | NO | 0.011 | 0.022 | YES | YES |
|  |  |  | *MPI* | ILMN_1761262 | 0.058 | 0.015 | 1.45E-04 | 5.58E-03 | rs4886636 | -0.081 | 0.014 | 3.00E-09 | 0.012 | 0.013 | 3.82E-01 | NO | 0.011 | 0.020 | YES | YES |
|  |  |  | *SCAMP5* | ILMN_1753345 | 0.079 | 0.019 | 3.35E-05 | 1.45E-03 | rs12593542 | -0.170 | 0.037 | 3.55E-06 | 0.072 | 0.019 | 1.68E-04 | YES | 0.012 | 0.018 | YES | NO |
|  |  |  | *ULK3* | ILMN_1679495 | -0.071 | 0.012 | 1.78E-09 | 1.12E-07 | rs936229 | -0.134 | 0.013 | 7.88E-26 | -0.013 | 0.010 | 2.25E-01 | NO | 0.012 | 0.025 | YES | YES |
| 17p13.2 | rs2286672 | 17:4712617 | *INCA1* | ILMN_1704380 | 0.035 | 0.010 | 7.69E-04 | 2.66E-02 | rs4609912 | 0.043 | 0.010 | 1.48E-05 | 0.014 | 0.009 | 1.35E-01 | NO | 0.004 | 0.005 | YES | YES |
|  |  |  | *RNF167* | ILMN_1794726 | 0.187 | 0.028 | 1.12E-11 | 8.62E-10 | rs238245 | 0.306 | 0.018 | 1.61E-62 | 0.069 | 0.027 | 1.01E-02 | YES | 0.005 | 0.009 | YES | NO |
| 22q11.21 | rs7444 | 22:21976934 | *UBE2L3* | ILMN_1677877 | -0.183 | 0.018 | 4.97E-25 | 8.61E-23 | rs5998672 | -0.182 | 0.018 | 4.62E-25 | 0.000 | 0.000 | 0.00E+00 | NO | 0.028 | 0.077 | YES | YES |

| **Supplementary Material, Table S3** All significant eQTLs (q < 0.05) and associated eGenes detected at RNA-Seq (gene-level) with conditional and colocalisation results | | | | | | | | | | | | | | | | | | | |
| --- | --- | --- | --- | --- | --- | --- | --- | --- | --- | --- | --- | --- | --- | --- | --- | --- | --- | --- | --- |
| **Risk**  **Locus** | **GWAS SNP** | **Coordinate (hg19)** | **eGene** | **β** | **Std Err** | **P-Value** | **FDR q** | **best cis eQTL** | **β** | **Std Err** | **P-Value** | **β _cond_** | **Std Err _cond_** | **P-Value _cond_** | **Independent  to best** | **PP3** | **PP4** | **Shared Causal Variant** | **Candidate Causal eQTL/eGene** |
| 1p13.2 | rs2476601 | 1.114377568 | *DCLRE1B* | -0.330 | 0.098 | 8.41E-04 | 1.91E-02 | rs2358994 | -0.383 | 0.076 | 6.00E-07 | -0.019 | 0.077 | 8.05E-01 | NO | 0.015 | 0.025 | YES | YES |
| 1q42.3 | rs9782955 | 1.236039877 | *LYST* | 0.188 | 0.060 | 1.98E-03 | 3.79E-02 | rs12049060 | -0.289 | 0.070 | 3.80E-05 | 0.172 | 0.061 | 4.50E-03 | YES | 0.022 | 0.018 | NO | NO |
| 2p14 | rs268134 | 2.65608363 | *SPRED2* | 0.276 | 0.063 | 1.58E-05 | 5.06E-04 | rs268134 | 0.276 | 0.063 | 1.58E-05 | 0.000 | 0.000 | 0.00E+00 | NO | 0.051 | 0.043 | NO | NO |
| 3q25.33 | rs564799 | 3.159728987 | *IL12A* | 0.372 | 0.054 | 1.07E-11 | 9.70E-10 | rs485499 | 0.387 | 0.052 | 3.47E-13 | 0.000 | 0.000 | 0.00E+00 | NO | 0.035 | 0.076 | YES | YES |
| 3p14.3 | rs11714389 | 3.58447299 | *PXK* | 0.382 | 0.054 | 5.62E-12 | 5.18E-10 | rs7633553 | 0.655 | 0.052 | 1.49E-32 | 0.719 | 0.048 | 7.90E-51 | YES | 0.060 | 0.098 | YES | NO |
|  |  |  | *ABHD6* | 0.320 | 0.055 | 7.00E-09 | 2.28E-07 | rs4681827 | 0.521 | 0.053 | 1.07E-21 | -0.021 | 0.042 | 6.14E-01 | NO | 0.039 | 0.075 | YES | YES |
|  |  |  | *RPP14* | -0.285 | 0.056 | 4.15E-07 | 1.64E-05 | rs12634722 | 0.373 | 0.055 | 2.47E-11 | -0.387 | 0.043 | 3.63E-01 | NO | 0.037 | 0.059 | YES | YES |
| 4q24 | rs10028805 | 4.10273725 | *BANK1* | -0.316 | 0.056 | 2.37E-08 | 1.29E-06 | rs7656409 | -0.448 | 0.055 | 9.86E-16 | -0.041 | 0.033 | 2.15E-01 | NO | 0.074 | 0.106 | YES | YES |
| 5q31.1 | rs17167273 | 5.133459347 | *SKP1* | -0.564 | 0.130 | 1.57E-05 | 5.06E-04 | rs254057 | -0.468 | 0.115 | 5.47E-05 | 0.000 | 0.000 | 0.00E+00 | NO | 0.035 | 0.038 | YES | YES |
|  |  |  | *TCF7* | -0.534 | 0.127 | 2.89E-05 | 8.27E-04 | rs151822 | 0.278 | 0.062 | 8.45E-06 | 0.000 | 0.000 | 0.00E+00 | NO | 0.034 | 0.046 | YES | YES |
| 5q33.3 | rs2431697 | 5.159879978 | *MIR146A* | 0.256 | 0.053 | 1.52E-06 | 6.89E-05 | rs2431697 | 0.256 | 0.053 | 1.52E-06 | 0.000 | 0.000 | 0.00E+00 | NO | 0.048 | 0.054 | YES | YES |
| 7q32.1 | rs4728142 | 7.128573967 | *IRF5* | 0.331 | 0.052 | 4.51E-10 | 3.07E-08 | rs729302 | 0.376 | 0.055 | 1.34E-11 | 0.545 | 0.043 | 6.17E-37 | YES | 0.061 | 0.068 | YES | NO |
|  |  |  | *TNPO3* | -0.233 | 0.054 | 1.71E-05 | 5.17E-04 | rs3807306 | -0.273 | 0.057 | 2.07E-06 | -0.023 | 0.031 | 4.61E-01 | NO | 0.062 | 0.040 | NO | NO |
| 7q32.1 | rs10488631 | 7.128594183 | *IRF5* | 0.337 | 0.078 | 1.55E-05 | 5.06E-04 | rs729302 | 0.376 | 0.055 | 1.34E-11 | 0.493 | 0.075 | 4.38E-11 | YES | 0.061 | 0.068 | YES | NO |
| 8p23.1 | rs2736340 | 8.11343973 | *AF131215.2* | 0.417 | 0.056 | 4.46E-13 | 4.85E-11 | rs4618656 | 0.727 | 0.049 | 8.72E-44 | 0.761 | 0.052 | 2.13E-48 | YES | 0.126 | 0.109 | NO | NO |
|  |  |  | *FAM167A* | 0.668 | 0.052 | 1.40E-33 | 7.62E-31 | rs2736340 | 0.668 | 0.052 | 1.40E-33 | 0.000 | 0.000 | 0.00E+00 | NO | 0.036 | 0.536 | YES | YES |
|  |  |  | *BLK* | -0.596 | 0.052 | 3.13E-28 | 8.51E-26 | rs2618473 | -0.628 | 0.053 | 1.08E-29 | 0.000 | 0.000 | 0.00E+00 | NO | 0.043 | 0.393 | YES | YES |
|  |  |  | *RP11-148O21.2* | -0.591 | 0.053 | 1.11E-26 | 2.01E-24 | rs1478901 | -0.624 | 0.055 | 1.53E-27 | 0.000 | 0.000 | 0.00E+00 | NO | 0.044 | 0.351 | YES | YES |
| 11p15.5 | rs2396545 | 11.601785 | *TMEM80* | 0.375 | 0.063 | 3.43E-09 | 2.07E-07 | rs7118663 | -0.437 | 0.050 | 1.38E-17 | 0.162 | 0.059 | 5.76E-03 | YES | 0.022 | 0.099 | YES | NO |
|  |  |  | *RNH1* | -0.243 | 0.066 | 2.47E-04 | 6.11E-03 | rs10540 | -0.559 | 0.078 | 2.03E-12 | -0.426 | 0.061 | 2.98E-12 | YES | 0.020 | 0.050 | YES | NO |
|  |  |  | *HRAS* | -0.248 | 0.065 | 1.56E-04 | 4.04E-03 | rs2613996 | -0.260 | 0.056 | 3.44E-06 | -0.098 | 0.057 | 8.76E-02 | NO | 0.019 | 0.051 | YES | YES |
| 11q13.4 | rs3794060 | 11.71187679 | *NADSYN1* | -0.620 | 0.059 | 6.43E-24 | 8.74E-22 | rs7928249 | -0.631 | 0.060 | 2.69E-24 | 0.000 | 0.000 | 0.00E+00 | NO | 0.014 | 0.110 | YES | YES |
|  |  |  | *DHCR7* | -0.200 | 0.064 | 1.70E-03 | 3.43E-02 | rs2852853 | -0.273 | 0.065 | 2.92E-05 | 0.037 | 0.029 | 1.96E-01 | NO | 0.010 | 0.015 | YES | YES |
| 12q24.32 | rs1059312 | 12.12927886 | *SLC15A4* | 0.188 | 0.054 | 5.15E-04 | 1.22E-02 | rs10773580 | 0.255 | 0.064 | 6.85E-05 | 0.042 | 0.040 | 2.93E-01 | NO | 0.060 | 0.040 | NO | NO |
| 15q24.2 | rs2289583 | 15.75311036 | *MPI* | -0.391 | 0.058 | 4.46E-11 | 3.47E-09 | rs4886636 | 0.538 | 0.049 | 1.27E-25 | -0.702 | 0.052 | 5.63E-42 | YES | 0.019 | 0.062 | YES | NO |
|  |  |  | *ULK3* | 0.303 | 0.059 | 3.62E-07 | 1.79E-05 | rs9210 | 0.587 | 0.058 | 3.01E-22 | -0.033 | 0.048 | 4.98E-01 | NO | 0.015 | 0.067 | YES | YES |
|  |  |  | *UBE2Q2* | 0.249 | 0.061 | 5.66E-05 | 1.54E-03 | rs11072518 | 0.267 | 0.057 | 3.85E-06 | 0.069 | 0.048 | 1.50E-01 | NO | 0.012 | 0.025 | YES | YES |
|  |  |  | *FAM219B* | -0.198 | 0.061 | 1.18E-03 | 2.57E-02 | rs6495126 | -0.217 | 0.060 | 3.15E-04 | -0.050 | 0.045 | 2.72E-01 | NO | 0.012 | 0.022 | YES | YES |
|  |  |  | *CSK* | 0.194 | 0.061 | 1.64E-03 | 3.43E-02 | rs1378941 | 0.298 | 0.059 | 4.62E-07 | 0.027 | 0.052 | 6.07E-01 | NO | 0.012 | 0.030 | YES | YES |
| 22q11.21 | rs7444 | 22.21976934 | *UBE2L3* | 0.345 | 0.072 | 2.28E-06 | 9.54E-05 | rs4820091 | 0.380 | 0.071 | 1.46E-07 | 0.000 | 0.000 | 0.00E+00 | NO | 0.027 | 0.077 | YES | YES |

| **Supplementary Material, Table S4** All significant eQTLs (q < 0.05) and associated eGenes detected at RNA-Seq (exon-level) with conditional and colocalisation results | | | | | | | | | | | | | | | | | | | | |
| --- | --- | --- | --- | --- | --- | --- | --- | --- | --- | --- | --- | --- | --- | --- | --- | --- | --- | --- | --- | --- |
| **Risk**  **Locus** | **GWAS SNP** | **Coordinate (hg19)** | **eGene** | **Exon ID (chr. start. end)** | **β** | **Std Err** | **P-Value** | **FDR q** | **best cis eQTL** | **β** | **Std Err** | **P-Value** | **β _cond_** | **Std Err _cond_** | **P-Value _cond_** | **Independent  to best** | **PP3** | **PP4** | **Shared Causal Variant** | **Candidate Causal eQTL/eGene** |
| 1p13.2 | **rs2476601** | 1:114377568 | ***BCL2L15*** | 1.114420790.114424619 | 0.226 | 0.035 | 2.71E-10 | 1.80E-08 | rs3761936 | 0.326 | 0.034 | 2.22E-20 | 0.051 | 0.030 | 8.96E-02 | NO | 0.020 | 0.037 | YES | YES |
|  |  |  |  | 1.114429871.114430169 | 0.199 | 0.035 | 2.72E-08 | 1.28E-06 | rs2358994 | 0.320 | 0.034 | 1.23E-19 | 0.041 | 0.031 | 1.87E-01 | NO | 0.020 | 0.035 | YES | YES |
|  |  |  | ***DCLRE1B*** | 1.114449618.114449783 | 0.110 | 0.036 | 2.23E-03 | 3.85E-02 | rs12046289 | 0.198 | 0.035 | 3.44E-08 | 0.042 | 0.034 | 2.11E-01 | NO | 0.019 | 0.022 | YES | YES |
|  |  |  | ***MAGI3*** | 1.114225519.114228545 | 0.115 | 0.036 | 1.41E-03 | 2.62E-02 | rs2488458 | 0.194 | 0.036 | 6.51E-08 | 0.044 | 0.034 | 1.87E-01 | NO | 0.017 | 0.020 | YES | YES |
|  |  |  |  | 1.114254368.114254457 | 0.109 | 0.036 | 2.59E-03 | 4.37E-02 | rs7514649 | 0.167 | 0.036 | 3.47E-06 | 0.122 | 0.036 | 6.59E-04 | YES | 0.018 | 0.018 | NO | NO |
|  |  |  |  | 1.114267957.114268583 | 0.113 | 0.036 | 1.78E-03 | 3.20E-02 | rs1935836 | 0.267 | 0.035 | 6.59E-14 | 0.143 | 0.036 | 6.42E-05 | YES | 0.019 | 0.021 | YES | NO |
| 1q23.3 | **rs1801274** | 1:161479745 | ***RP11-122G18.5*** | 1.161337812.161338955 | 0.151 | 0.036 | 2.67E-05 | 7.87E-04 | rs9330294 | 0.273 | 0.035 | 1.49E-14 | 0.125 | 0.036 | 5.54E-04 | YES | 0.098 | 0.049 | NO | NO |
| 1q25.1 | **rs844663** | 1:173243581 | ***GAS5*** | 1.173834956.173835934 | 0.108 | 0.036 | 2.81E-03 | 4.63E-02 | rs844654 | 0.156 | 0.036 | 1.41E-05 | 0.014 | 0.029 | 6.28E-01 | NO | 0.042 | 0.039 | NO | NO |
| 1q25.1 | **rs1935325** | 1:173336003 | ***TNFSF4*** | 1.173152873.173156004 | 0.132 | 0.036 | 2.45E-04 | 5.76E-03 | rs17346536 | 0.136 | 0.036 | 1.60E-04 | 0.080 | 0.034 | 1.66E-02 | YES | 0.056 | 0.035 | NO | NO |
| 1q32.1 | **rs3024505** | 1:206939904 | ***FCAMR*** | 1.207133024.207134568 | 0.114 | 0.036 | 1.62E-03 | 2.94E-02 | rs7539999 | 0.231 | 0.035 | 1.02E-10 | 0.034 | 0.034 | 3.18E-01 | NO | 0.002 | 0.003 | YES | YES |
|  |  |  | ***IL10*** | 1.206940947.206942073 | 0.107 | 0.036 | 3.06E-03 | 4.97E-02 | rs7521798 | 0.141 | 0.036 | 8.96E-05 | 0.071 | 0.035 | 4.22E-02 | YES | 0.002 | 0.003 | YES | NO |
|  |  |  |  | 1.206944252.206944544 | 0.161 | 0.036 | 7.73E-06 | 2.53E-04 | rs1554286 | 0.164 | 0.036 | 5.26E-06 | 0.195 | 0.035 | 2.81E-08 | YES | 0.002 | 0.003 | YES | NO |
|  |  |  |  | 1.206945616.206945839 | 0.174 | 0.036 | 1.26E-06 | 4.88E-05 | rs3024493 | 0.175 | 0.036 | 1.14E-06 | 0.000 | 0.000 | 0.00E+00 | NO | 0.002 | 0.003 | YES | YES |
|  |  |  | ***IL24*** | 1.207076321.207077484 | 0.108 | 0.036 | 2.81E-03 | 4.63E-02 | rs94872 | 0.228 | 0.035 | 1.71E-10 | 0.060 | 0.035 | 9.12E-02 | NO | 0.002 | 0.003 | YES | YES |
|  |  |  | ***MAPKAPK2*** | 1.206905151.206907628 | 0.126 | 0.036 | 4.93E-04 | 1.06E-02 | rs12140898 | 0.207 | 0.035 | 7.73E-09 | 0.083 | 0.035 | 1.83E-02 | YES | 0.002 | 0.003 | YES | NO |
| 1q42.3 | **rs9782955** | 1:236039877 | ***LYST*** | 1.235824341.235826378 | 0.137 | 0.036 | 1.39E-04 | 3.45E-03 | rs10927069 | 0.239 | 0.035 | 2.29E-11 | 0.157 | 0.036 | 1.41E-05 | YES | 0.024 | 0.018 | NO | NO |
|  |  |  | ***NID1*** | 1.236205210.236205592 | 0.108 | 0.036 | 2.82E-03 | 4.63E-02 | rs2734808 | 0.167 | 0.036 | 3.54E-06 | 0.118 | 0.036 | 1.05E-03 | YES | 0.023 | 0.019 | NO | NO |
| 2q24.2 | **rs2111485** | 2:163110536 | ***IFIH1*** | 2.163123589.163123889 | 0.119 | 0.036 | 1.00E-03 | 1.98E-02 | rs2111485 | 0.119 | 0.036 | 1.00E-03 | 0.000 | 0.000 | 0.00E+00 | NO | 0.020 | 0.024 | YES | YES |
|  |  |  |  | 2.163123989.163124079 | 0.115 | 0.036 | 1.50E-03 | 2.79E-02 | rs608665 | 0.120 | 0.036 | 8.40E-04 | 0.111 | 0.036 | 2.30E-03 | YES | 0.020 | 0.024 | YES | NO |
|  |  |  |  | 2.163128736.163128897 | 0.108 | 0.036 | 2.69E-03 | 4.49E-02 | rs1549020 | 0.109 | 0.036 | 2.48E-03 | 0.049 | 0.030 | 1.09E-01 | NO | 0.020 | 0.024 | YES | YES |
|  |  |  |  | 2.163130305.163130454 | 0.116 | 0.036 | 1.25E-03 | 2.37E-02 | rs2111485 | 0.116 | 0.036 | 1.25E-03 | 0.000 | 0.000 | 0.00E+00 | NO | 0.020 | 0.024 | YES | YES |
|  |  |  |  | 2.163136506.163136622 | 0.138 | 0.036 | 1.31E-04 | 3.29E-03 | rs2111485 | 0.138 | 0.036 | 1.31E-04 | 0.000 | 0.000 | 0.00E+00 | NO | 0.020 | 0.025 | YES | YES |
|  |  |  |  | 2.163137838.163138055 | 0.110 | 0.036 | 2.34E-03 | 4.01E-02 | rs2111485 | 0.110 | 0.036 | 2.34E-03 | 0.000 | 0.000 | 0.00E+00 | NO | 0.020 | 0.024 | YES | YES |
|  |  |  |  | 2.163163219.163163680 | 0.127 | 0.036 | 4.45E-04 | 9.72E-03 | rs2111485 | 0.127 | 0.036 | 4.45E-04 | 0.000 | 0.000 | 0.00E+00 | NO | 0.020 | 0.025 | YES | YES |
| 2q32.3 | **rs11889341** | 2:191943742 | ***NAB1*** | 2.191520703.191520885 | 0.117 | 0.036 | 1.16E-03 | 2.21E-02 | rs2293765 | 0.486 | 0.032 | 1.58E-46 | 0.023 | 0.035 | 5.08E-01 | NO | 0.084 | 0.039 | NO | NO |
| 2q32.3 | **rs16833249** | 2:191948272 | ***STAT4*** | 2.191897588.191897875 | 0.114 | 0.036 | 1.56E-03 | 2.88E-02 | rs16833437 | 0.508 | 0.031 | 1.68E-51 | 0.025 | 0.036 | 4.84E-01 | NO | 0.093 | 0.034 | NO | NO |
| 2q34 | **rs3768792** | 2:213871709 | ***IKZF2*** | 2.213878515.213880002 | 0.201 | 0.035 | 2.08E-08 | 1.01E-06 | rs10178238 | 0.295 | 0.035 | 8.38E-17 | 0.122 | 0.034 | 3.89E-04 | YES | 0.059 | 0.050 | NO | NO |
| 2p14 | **rs268134** | 2:65608363 | ***SPRED2*** | 2.65537985.65541303 | 0.218 | 0.035 | 1.05E-09 | 6.18E-08 | rs268134 | 0.218 | 0.035 | 1.05E-09 | 0.000 | 0.000 | 0.00E+00 | NO | 0.056 | 0.045 | NO | NO |
|  |  |  |  | 2.65543868.65544017 | 0.135 | 0.036 | 1.87E-04 | 4.49E-03 | rs874952 | 0.147 | 0.036 | 4.69E-05 | 0.042 | 0.028 | 1.37E-01 | NO | 0.056 | 0.038 | NO | NO |
| 3q25.33 | **rs564799** | 3:159728987 | ***IFT80*** | 3.159996981.159997152 | 0.109 | 0.036 | 2.65E-03 | 4.44E-02 | rs574808 | 0.125 | 0.036 | 5.05E-04 | 0.000 | 0.000 | 0.00E+00 | NO | 0.028 | 0.040 | YES | YES |
|  |  |  | ***IL12A*** | 3.159706537.159706961 | 0.202 | 0.035 | 1.66E-08 | 8.24E-07 | rs485499 | 0.205 | 0.035 | 1.12E-08 | 0.000 | 0.000 | 0.00E+00 | NO | 0.037 | 0.048 | YES | YES |
|  |  |  |  | 3.159707954.159708119 | 0.142 | 0.036 | 8.00E-05 | 2.09E-03 | rs583911 | 0.161 | 0.036 | 7.87E-06 | 0.223 | 0.031 | 1.19E-12 | YES | 0.037 | 0.041 | YES | NO |
|  |  |  |  | 3.159710799.159711631 | 0.286 | 0.035 | 7.56E-16 | 8.33E-14 | rs574808 | 0.291 | 0.035 | 2.17E-16 | 0.000 | 0.000 | 0.00E+00 | NO | 0.038 | 0.073 | YES | YES |
|  |  |  |  | 3.159713191.159713806 | 0.281 | 0.035 | 2.23E-15 | 2.31E-13 | rs2366643 | 0.282 | 0.035 | 1.67E-15 | 0.000 | 0.000 | 0.00E+00 | NO | 0.038 | 0.069 | YES | YES |
|  |  |  | ***KPNA4*** | 3.160283002.160283376 | 0.111 | 0.036 | 2.20E-03 | 3.84E-02 | rs4679891 | 0.284 | 0.035 | 1.26E-15 | 0.161 | 0.036 | 6.60E-06 | YES | 0.029 | 0.038 | YES | NO |
|  |  |  | ***RP11-432B6.3*** | 3.159996981.159997152 | 0.109 | 0.036 | 2.65E-03 | 4.44E-02 | rs574808 | 0.125 | 0.036 | 5.05E-04 | 0.000 | 0.000 | 0.00E+00 | NO | 0.027 | 0.039 | YES | YES |
|  |  |  | ***SCHIP1*** | 3.159583952.159584070 | 0.164 | 0.036 | 4.93E-06 | 1.68E-04 | rs13323195 | 0.183 | 0.036 | 3.40E-07 | 0.167 | 0.036 | 4.23E-06 | YES | 0.034 | 0.045 | YES | NO |
|  |  |  |  | 3.159603996.159606732 | 0.144 | 0.036 | 6.51E-05 | 1.77E-03 | rs9878736 | 0.215 | 0.035 | 1.98E-09 | 0.180 | 0.036 | 4.79E-07 | YES | 0.034 | 0.042 | YES | NO |
|  |  |  |  | 3.159609886.159612512 | 0.157 | 0.036 | 1.31E-05 | 4.10E-04 | rs9878736 | 0.170 | 0.036 | 2.30E-06 | 0.186 | 0.036 | 2.20E-07 | YES | 0.034 | 0.043 | YES | NO |
|  |  |  |  | 3.159614512.159615149 | 0.125 | 0.036 | 5.36E-04 | 1.14E-02 | rs9878736 | 0.182 | 0.036 | 3.91E-07 | 0.156 | 0.036 | 1.34E-05 | YES | 0.034 | 0.040 | YES | NO |
|  |  |  | ***SMC4*** | 3.160129546.160129872 | 0.120 | 0.036 | 8.69E-04 | 1.74E-02 | rs10923 | 0.341 | 0.034 | 2.90E-22 | 0.060 | 0.036 | 9.39E-02 | NO | 0.034 | 0.041 | YES | YES |
|  |  |  |  | 3.160137146.160137331 | 0.112 | 0.036 | 1.97E-03 | 3.48E-02 | rs10936201 | 0.308 | 0.034 | 2.85E-18 | 0.055 | 0.036 | 1.22E-01 | NO | 0.033 | 0.039 | YES | YES |
|  |  |  |  | 3.160138370.160138689 | 0.157 | 0.036 | 1.23E-05 | 3.87E-04 | rs17236494 | 0.239 | 0.035 | 2.12E-11 | 0.114 | 0.036 | 1.35E-03 | YES | 0.031 | 0.043 | YES | NO |
|  |  |  |  | 3.160141213.160141438 | 0.112 | 0.036 | 1.98E-03 | 3.48E-02 | rs11720689 | 0.300 | 0.035 | 2.31E-17 | 0.058 | 0.036 | 1.05E-01 | NO | 0.032 | 0.040 | YES | YES |
|  |  |  |  | 3.160141549.160141628 | 0.110 | 0.036 | 2.36E-03 | 4.03E-02 | rs11720689 | 0.268 | 0.035 | 4.58E-14 | 0.062 | 0.036 | 8.41E-02 | NO | 0.031 | 0.038 | YES | YES |
|  |  |  |  | 3.160142655.160142807 | 0.139 | 0.036 | 1.17E-04 | 2.96E-03 | rs10923 | 0.339 | 0.034 | 4.97E-22 | 0.079 | 0.036 | 2.73E-02 | YES | 0.034 | 0.041 | YES | NO |
|  |  |  |  | 3.160143862.160143988 | 0.167 | 0.036 | 3.28E-06 | 1.16E-04 | rs11709232 | 0.295 | 0.035 | 8.66E-17 | 0.114 | 0.036 | 1.43E-03 | YES | 0.033 | 0.044 | YES | NO |
|  |  |  |  | 3.160146541.160146730 | 0.129 | 0.036 | 3.62E-04 | 8.08E-03 | rs11708118 | 0.308 | 0.034 | 2.81E-18 | 0.073 | 0.036 | 4.10E-02 | YES | 0.032 | 0.042 | YES | NO |
|  |  |  |  | 3.160148387.160148993 | 0.137 | 0.036 | 1.37E-04 | 3.42E-03 | rs11720689 | 0.334 | 0.034 | 2.34E-21 | 0.077 | 0.036 | 3.01E-02 | YES | 0.033 | 0.043 | YES | NO |
|  |  |  |  | 3.160149431.160149613 | 0.126 | 0.036 | 4.69E-04 | 1.02E-02 | rs11720689 | 0.345 | 0.034 | 7.53E-23 | 0.064 | 0.036 | 7.25E-02 | NO | 0.034 | 0.041 | YES | YES |
|  |  |  |  | 3.160150071.160150303 | 0.122 | 0.036 | 7.51E-04 | 1.53E-02 | rs11708118 | 0.341 | 0.034 | 3.07E-22 | 0.060 | 0.036 | 9.25E-02 | NO | 0.034 | 0.041 | YES | YES |
|  |  |  |  | 3.160150814.160150997 | 0.137 | 0.036 | 1.41E-04 | 3.47E-03 | rs10923 | 0.383 | 0.033 | 4.16E-28 | 0.069 | 0.036 | 5.20E-02 | NO | 0.038 | 0.043 | YES | YES |
|  |  | 3:159728987 | ***TRIM59*** | 3.160150233.160156974 | 0.118 | 0.036 | 1.09E-03 | 2.11E-02 | rs11720689 | 0.214 | 0.035 | 2.08E-09 | 0.079 | 0.036 | 2.61E-02 | YES | 0.028 | 0.043 | YES | NO |
| 3p14.3 | **rs11714389** | 3:58447299 | ***ABHD6*** | 3.58223233.58223643 | 0.132 | 0.036 | 2.62E-04 | 6.10E-03 | rs10866014 | 0.190 | 0.036 | 1.24E-07 | 0.004 | 0.027 | 8.79E-01 | NO | 0.026 | 0.043 | YES | YES |
|  |  |  |  | 3.58242289.58242432 | 0.173 | 0.036 | 1.57E-06 | 5.89E-05 | rs6802482 | 0.267 | 0.035 | 5.43E-14 | 0.001 | 0.028 | 9.74E-01 | NO | 0.027 | 0.050 | YES | YES |
|  |  |  |  | 3.58252916.58253072 | 0.174 | 0.036 | 1.34E-06 | 5.11E-05 | rs4342080 | 0.297 | 0.035 | 4.46E-17 | -0.017 | 0.028 | 5.49E-01 | NO | 0.027 | 0.050 | YES | YES |
|  |  |  |  | 3.58255048.58255161 | 0.207 | 0.035 | 7.18E-09 | 3.76E-07 | rs4681828 | 0.270 | 0.035 | 2.75E-14 | 0.032 | 0.028 | 2.51E-01 | NO | 0.027 | 0.058 | YES | YES |
|  |  |  |  | 3.58256659.58256791 | 0.190 | 0.036 | 1.21E-07 | 5.27E-06 | rs4681827 | 0.261 | 0.035 | 2.40E-13 | 0.019 | 0.027 | 4.81E-01 | NO | 0.027 | 0.053 | YES | YES |
|  |  |  |  | 3.58260385.58260542 | 0.200 | 0.035 | 2.47E-08 | 1.18E-06 | rs6445960 | 0.329 | 0.034 | 9.42E-21 | -0.014 | 0.028 | 6.18E-01 | NO | 0.028 | 0.056 | YES | YES |
|  |  |  |  | 3.58270924.58271180 | 0.221 | 0.035 | 6.02E-10 | 3.75E-08 | rs4681827 | 0.306 | 0.034 | 4.82E-18 | 0.021 | 0.027 | 4.41E-01 | NO | 0.028 | 0.062 | YES | YES |
|  |  |  |  | 3.58279316.58280461 | 0.345 | 0.034 | 7.89E-23 | 1.13E-20 | rs6445960 | 0.458 | 0.032 | 6.57E-41 | 0.048 | 0.028 | 8.58E-02 | NO | 0.034 | 0.141 | YES | YES |
|  |  |  | ***PDHB*** | 3.58413357.58414341 | 0.153 | 0.036 | 2.14E-05 | 6.53E-04 | rs9811074 | 0.306 | 0.034 | 5.21E-18 | 0.199 | 0.036 | 2.76E-08 | YES | 0.035 | 0.046 | YES | NO |
|  |  |  |  | 3.58416384.58419584 | 0.137 | 0.036 | 1.46E-04 | 3.57E-03 | rs13071688 | 0.313 | 0.034 | 7.56E-19 | 0.184 | 0.036 | 2.98E-07 | YES | 0.035 | 0.043 | YES | NO |
|  |  |  | ***PXK*** | 3.58368241.58368427 | 0.146 | 0.036 | 5.35E-05 | 1.48E-03 | rs9862378 | 0.290 | 0.035 | 2.91E-16 | 0.285 | 0.032 | 2.11E-19 | YES | 0.031 | 0.045 | YES | NO |
|  |  |  |  | 3.58380725.58380829 | 0.182 | 0.036 | 3.98E-07 | 1.69E-05 | rs7633553 | 0.298 | 0.035 | 4.24E-17 | 0.335 | 0.031 | 6.19E-27 | YES | 0.031 | 0.052 | YES | NO |
|  |  |  |  | 3.58381385.58381486 | 0.149 | 0.036 | 3.63E-05 | 1.03E-03 | rs7633553 | 0.315 | 0.034 | 4.32E-19 | 0.310 | 0.031 | 1.63E-23 | YES | 0.031 | 0.046 | YES | NO |
|  |  |  |  | 3.58382766.58382927 | 0.219 | 0.035 | 8.53E-10 | 5.20E-08 | rs9862378 | 0.345 | 0.034 | 8.27E-23 | 0.384 | 0.032 | 1.50E-33 | YES | 0.032 | 0.061 | YES | NO |
|  |  |  |  | 3.58383332.58383449 | 0.219 | 0.035 | 9.96E-10 | 5.96E-08 | rs7633553 | 0.303 | 0.035 | 1.06E-17 | 0.374 | 0.031 | 5.03E-33 | YES | 0.030 | 0.061 | YES | NO |
|  |  |  |  | 3.58385026.58385104 | 0.180 | 0.036 | 5.68E-07 | 2.32E-05 | rs7633553 | 0.260 | 0.035 | 2.97E-13 | 0.313 | 0.031 | 1.03E-23 | YES | 0.029 | 0.051 | YES | NO |
|  |  |  |  | 3.58394651.58394728 | 0.132 | 0.036 | 2.53E-04 | 5.92E-03 | rs6925 | 0.307 | 0.034 | 3.39E-18 | 0.271 | 0.032 | 3.53E-17 | YES | 0.031 | 0.044 | YES | NO |
|  |  |  |  | 3.58395259.58395345 | 0.211 | 0.035 | 4.04E-09 | 2.20E-07 | rs7633553 | 0.244 | 0.035 | 7.09E-12 | 0.336 | 0.031 | 5.81E-27 | YES | 0.029 | 0.059 | YES | NO |
|  |  |  |  | 3.58395817.58395886 | 0.169 | 0.036 | 2.46E-06 | 8.86E-05 | rs7633553 | 0.248 | 0.035 | 3.30E-12 | 0.297 | 0.031 | 1.63E-21 | YES | 0.029 | 0.049 | YES | NO |
|  |  |  |  | 3.58398628.58399259 | 0.214 | 0.035 | 2.18E-09 | 1.23E-07 | rs7633553 | 0.312 | 0.034 | 9.07E-19 | 0.375 | 0.031 | 3.94E-33 | YES | 0.031 | 0.060 | YES | NO |
|  |  |  |  | 3.58410479.58411748 | 0.309 | 0.034 | 1.99E-18 | 2.51E-16 | rs9862378 | 0.480 | 0.032 | 2.18E-45 | 0.527 | 0.032 | 9.72E-60 | YES | 0.043 | 0.109 | YES | NO |
|  |  |  | ***RPP14*** | 3.58303167.58305816 | 0.213 | 0.035 | 2.53E-09 | 1.41E-07 | rs6767498 | 0.280 | 0.035 | 3.09E-15 | 0.397 | 0.027 | 1.15E-47 | YES | 0.029 | 0.059 | YES | NO |
|  |  |  |  | 3.58303167.58305920 | 0.223 | 0.035 | 4.78E-10 | 3.02E-08 | rs6767498 | 0.282 | 0.035 | 1.99E-15 | 0.407 | 0.027 | 3.87E-50 | YES | 0.029 | 0.062 | YES | NO |
| 4q24 | **rs10028805** | 4:10273725 | ***BANK1*** | 4.102750965.102751363 | 0.323 | 0.034 | 4.99E-20 | 6.62E-18 | rs4572885 | 0.344 | 0.034 | 9.74E-23 | 0.088 | 0.027 | 1.12E-03 | YES | 0.065 | 0.107 | YES | NO |
|  |  |  |  | 4.102776192.102776346 | 0.262 | 0.035 | 1.78E-13 | 1.56E-11 | rs4572885 | 0.277 | 0.035 | 6.16E-15 | 0.073 | 0.027 | 6.37E-03 | YES | 0.063 | 0.071 | YES | NO |
|  |  |  |  | 4.102783683.102783821 | 0.252 | 0.035 | 1.49E-12 | 1.22E-10 | rs4572885 | 0.274 | 0.035 | 1.19E-14 | 0.065 | 0.027 | 1.50E-02 | YES | 0.063 | 0.069 | YES | NO |
|  |  |  |  | 4.102791662.102791801 | 0.215 | 0.035 | 1.91E-09 | 1.08E-07 | rs12331595 | 0.256 | 0.035 | 6.52E-13 | 0.375 | 0.028 | 4.29E-40 | YES | 0.063 | 0.059 | NO | NO |
|  |  |  |  | 4.102839150.102839346 | 0.245 | 0.035 | 6.86E-12 | 4.94E-10 | rs4572885 | 0.263 | 0.035 | 1.30E-13 | 0.065 | 0.027 | 1.51E-02 | YES | 0.063 | 0.067 | YES | NO |
|  |  |  |  | 4.102946358.102946666 | 0.264 | 0.035 | 1.07E-13 | 9.91E-12 | rs6833764 | 0.288 | 0.035 | 4.06E-16 | 0.031 | 0.021 | 1.41E-01 | NO | 0.064 | 0.077 | YES | YES |
|  |  |  |  | 4.102981368.102981546 | 0.253 | 0.035 | 1.19E-12 | 9.92E-11 | rs7656409 | 0.287 | 0.035 | 6.21E-16 | 0.024 | 0.022 | 2.62E-01 | NO | 0.064 | 0.071 | YES | YES |
|  |  |  |  | 4.102982573.102984295 | 0.250 | 0.035 | 2.40E-12 | 1.91E-10 | rs12331595 | 0.271 | 0.035 | 2.25E-14 | 0.420 | 0.028 | 1.41E-49 | YES | 0.063 | 0.069 | YES | NO |
|  |  |  |  | 4.102995162.102995969 | 0.294 | 0.035 | 9.56E-17 | 1.08E-14 | rs4572885 | 0.315 | 0.034 | 4.71E-19 | 0.080 | 0.027 | 3.09E-03 | YES | 0.065 | 0.088 | YES | NO |
| 5q31.1 | **rs17167273** | 5:133459347 | ***CDKN2AIPNL*** | 5.133737778.133738617 | 0.112 | 0.036 | 1.92E-03 | 3.41E-02 | rs17517610 | 0.134 | 0.036 | 2.09E-04 | 0.111 | 0.036 | 2.13E-03 | YES | 0.037 | 0.039 | YES | NO |
|  |  |  | ***SKP1*** | 5.133492082.133494319 | 0.276 | 0.035 | 7.20E-15 | 7.17E-13 | rs17167326 | 0.279 | 0.035 | 3.93E-15 | 0.000 | 0.000 | 0.00E+00 | NO | 0.043 | 0.051 | YES | YES |
|  |  |  |  | 5.133496678.133497588 | 0.175 | 0.036 | 1.08E-06 | 4.28E-05 | rs17167326 | 0.177 | 0.036 | 8.26E-07 | 0.000 | 0.000 | 0.00E+00 | NO | 0.041 | 0.040 | NO | NO |
|  |  |  |  | 5.133502861.133502934 | 0.120 | 0.036 | 8.84E-04 | 1.77E-02 | rs17167275 | 0.124 | 0.036 | 6.12E-04 | 0.083 | 0.034 | 1.56E-02 | YES | 0.040 | 0.039 | NO | NO |
|  |  |  |  | 5.133509078.133509713 | 0.128 | 0.036 | 3.88E-04 | 8.59E-03 | rs2436447 | 0.142 | 0.036 | 8.36E-05 | 0.130 | 0.036 | 3.16E-04 | YES | 0.041 | 0.038 | NO | NO |
|  |  |  |  | 5.133512305.133512729 | 0.110 | 0.036 | 2.22E-03 | 3.85E-02 | rs13354409 | 0.124 | 0.036 | 5.69E-04 | 0.000 | 0.016 | 9.85E-01 | NO | 0.041 | 0.037 | NO | NO |
|  |  |  | ***TCF7*** | 5.133451254.133451777 | 0.108 | 0.036 | 2.86E-03 | 4.68E-02 | rs30499 | 0.204 | 0.035 | 1.18E-08 | 0.080 | 0.036 | 2.59E-02 | YES | 0.043 | 0.039 | NO | NO |
|  |  |  |  | 5.133473556.133473928 | 0.158 | 0.036 | 1.07E-05 | 3.42E-04 | rs151822 | 0.242 | 0.035 | 1.23E-11 | 0.112 | 0.035 | 1.47E-03 | YES | 0.044 | 0.040 | NO | NO |
|  |  |  |  | 5.133474292.133475549 | 0.115 | 0.036 | 1.41E-03 | 2.62E-02 | rs30494 | 0.262 | 0.035 | 1.96E-13 | 0.050 | 0.035 | 1.52E-01 | NO | 0.044 | 0.037 | NO | NO |
|  |  |  |  | 5.133477155.133477995 | 0.146 | 0.036 | 4.85E-05 | 1.36E-03 | rs151822 | 0.285 | 0.035 | 9.78E-16 | 0.091 | 0.035 | 9.50E-03 | YES | 0.044 | 0.038 | NO | NO |
|  |  |  |  | 5.133478412.133479168 | 0.175 | 0.036 | 1.14E-06 | 4.45E-05 | rs244948 | 0.299 | 0.035 | 2.84E-17 | 0.100 | 0.035 | 3.78E-03 | YES | 0.045 | 0.040 | NO | NO |
|  |  |  |  | 5.133479283.133483927 | 0.229 | 0.035 | 1.49E-10 | 1.01E-08 | rs151822 | 0.341 | 0.034 | 2.49E-22 | 0.165 | 0.035 | 2.26E-06 | YES | 0.045 | 0.044 | NO | NO |
| 5q33.1 | **rs10036748** | 5:150458146 | ***CCDC69*** | 5.150566947.150567020 | 0.121 | 0.036 | 7.82E-04 | 1.58E-02 | rs919727 | 0.211 | 0.035 | 3.62E-09 | 0.125 | 0.036 | 5.68E-04 | YES | 0.049 | 0.042 | NO | NO |
|  |  |  | ***TNIP1*** | 5.150425422.150425922 | 0.133 | 0.036 | 2.15E-04 | 5.12E-03 | rs4958880 | 0.163 | 0.036 | 5.71E-06 | 0.007 | 0.023 | 7.50E-01 | NO | 0.052 | 0.043 | NO | NO |
| 5q33.3 | **rs2431697** | 5:159879978 | ***MIR146A*** | 5.159895275.159895447 | 0.151 | 0.036 | 2.74E-05 | 7.97E-04 | rs2431697 | 0.151 | 0.036 | 2.74E-05 | 0.000 | 0.000 | 0.00E+00 | NO | 0.044 | 0.057 | YES | YES |
|  |  |  |  | 5.159912306.159914433 | 0.248 | 0.035 | 3.36E-12 | 2.52E-10 | rs2431697 | 0.248 | 0.035 | 3.36E-12 | 0.000 | 0.000 | 0.00E+00 | NO | 0.053 | 0.070 | YES | YES |
|  |  |  | ***PTTG1*** | 5.159854722.159854880 | 0.139 | 0.036 | 1.20E-04 | 3.03E-03 | rs2910199 | 0.202 | 0.035 | 1.73E-08 | 0.068 | 0.034 | 4.70E-02 | YES | 0.054 | 0.046 | NO | NO |
|  |  |  |  | 5.159855609.159855748 | 0.136 | 0.036 | 1.67E-04 | 4.03E-03 | rs2910202 | 0.205 | 0.035 | 1.00E-08 | 0.059 | 0.034 | 8.20E-02 | NO | 0.054 | 0.045 | NO | NO |
| 7q32.1 | **rs4728142** | 7:128573967 | ***AHCYL2*** | 7.129019479.129019590 | 0.119 | 0.036 | 1.01E-03 | 1.98E-02 | rs4728142 | 0.119 | 0.036 | 1.01E-03 | 0.000 | 0.000 | 0.00E+00 | NO | 0.056 | 0.037 | NO | NO |
|  |  |  | ***ATP6V1F*** | 7.128502880.128503116 | 0.142 | 0.036 | 8.35E-05 | 2.17E-03 | rs11761199 | 0.180 | 0.036 | 5.43E-07 | 0.004 | 0.023 | 8.54E-01 | NO | 0.061 | 0.041 | NO | NO |
|  |  |  | ***IRF5*** | 7.128577666.128577888 | 0.420 | 0.033 | 5.76E-34 | 1.07E-31 | rs3807306 | 0.421 | 0.033 | 3.48E-34 | 0.054 | 0.019 | 4.24E-03 | YES | 0.068 | 0.068 | NO | NO |
|  |  |  |  | 7.128577991.128578103 | 0.419 | 0.033 | 6.00E-34 | 1.07E-31 | rs4728142 | 0.419 | 0.033 | 6.00E-34 | 0.000 | 0.000 | 0.00E+00 | NO | 0.072 | 0.055 | NO | NO |
|  |  |  |  | 7.128580260.128580517 | 0.263 | 0.035 | 1.42E-13 | 1.27E-11 | rs4728142 | 0.263 | 0.035 | 1.42E-13 | 0.000 | 0.000 | 0.00E+00 | NO | 0.065 | 0.039 | NO | NO |
|  |  |  |  | 7.128580726.128580929 | 0.221 | 0.035 | 6.32E-10 | 3.90E-08 | rs3807306 | 0.226 | 0.035 | 2.61E-10 | 0.024 | 0.018 | 1.76E-01 | NO | 0.064 | 0.040 | NO | NO |
|  |  |  |  | 7.128581333.128581478 | 0.185 | 0.036 | 2.62E-07 | 1.13E-05 | rs4728142 | 0.185 | 0.036 | 2.62E-07 | 0.000 | 0.000 | 0.00E+00 | NO | 0.064 | 0.037 | NO | NO |
|  |  |  |  | 7.128582125.128582330 | 0.442 | 0.032 | 7.22E-38 | 1.50E-35 | rs13242262 | 0.501 | 0.031 | 8.14E-50 | 0.742 | 0.031 | 2.3E-130 | YES | 0.094 | 0.060 | NO | NO |
|  |  |  |  | 7.128585899.128586088 | 0.461 | 0.032 | 1.71E-41 | 5.22E-39 | rs3807306 | 0.491 | 0.032 | 1.08E-47 | 0.047 | 0.020 | 1.89E-02 | YES | 0.093 | 0.061 | NO | NO |
|  |  |  |  | 7.128586555.128586996 | 0.444 | 0.032 | 2.50E-38 | 5.88E-36 | rs3807306 | 0.446 | 0.032 | 1.13E-38 | 0.057 | 0.019 | 2.73E-03 | YES | 0.077 | 0.048 | NO | NO |
|  |  |  |  | 7.128587077.128587589 | 0.444 | 0.032 | 2.76E-38 | 6.22E-36 | rs3807306 | 0.451 | 0.032 | 1.24E-39 | 0.054 | 0.019 | 4.71E-03 | YES | 0.074 | 0.111 | YES | NO |
|  |  |  |  | 7.128587783.128588175 | 0.463 | 0.032 | 6.83E-42 | 2.49E-39 | rs13242262 | 0.480 | 0.032 | 2.01E-45 | 0.757 | 0.031 | 6.78E-133 | YES | 0.088 | 0.062 | NO | NO |
|  |  |  |  | 7.128588264.128588382 | 0.476 | 0.032 | 1.52E-44 | 8.76E-42 | rs3807306 | 0.497 | 0.031 | 4.84E-49 | 0.054 | 0.020 | 6.86E-03 | YES | 0.089 | 0.069 | NO | NO |
|  |  |  | ***KCP*** | 7.128516919.128517365 | 0.184 | 0.036 | 2.96E-07 | 1.27E-05 | rs11761199 | 0.189 | 0.036 | 1.48E-07 | 0.039 | 0.023 | 9.24E-02 | NO | 0.065 | 0.035 | NO | NO |
|  |  |  |  | 7.128517538.128517844 | 0.157 | 0.036 | 1.32E-05 | 4.10E-04 | rs3807306 | 0.174 | 0.036 | 1.37E-06 | 0.006 | 0.018 | 7.48E-01 | NO | 0.063 | 0.036 | NO | NO |
|  |  |  |  | 7.128518612.128519677 | 0.170 | 0.036 | 2.11E-06 | 7.69E-05 | rs3807306 | 0.199 | 0.035 | 3.04E-08 | -0.002 | 0.018 | 9.18E-01 | NO | 0.064 | 0.036 | NO | NO |
|  |  |  |  | 7.128520673.128520977 | 0.123 | 0.036 | 6.76E-04 | 1.40E-02 | rs7779637 | 0.130 | 0.036 | 3.03E-04 | 0.101 | 0.036 | 4.77E-03 | YES | 0.063 | 0.035 | NO | NO |
|  |  |  | ***NRF1*** | 7.129273286.129273621 | 0.115 | 0.036 | 1.38E-03 | 2.59E-02 | rs17555614 | 0.181 | 0.036 | 4.48E-07 | 0.118 | 0.036 | 1.13E-03 | YES | 0.048 | 0.037 | NO | NO |
|  |  |  | ***RP11-155G14.6*** | 7.128106617.128106739 | 0.107 | 0.036 | 3.05E-03 | 4.97E-02 | rs4731457 | 0.217 | 0.035 | 1.27E-09 | 0.099 | 0.036 | 6.37E-03 | YES | 0.045 | 0.036 | NO | NO |
|  |  |  | ***SMO*** | 7.128851865.128853386 | 0.110 | 0.036 | 2.23E-03 | 3.85E-02 | rs4728152 | 0.243 | 0.035 | 8.74E-12 | 0.140 | 0.036 | 1.04E-04 | YES | 0.058 | 0.040 | NO | NO |
|  |  |  | ***TNPO3*** | 7.128594948.128595439 | 0.166 | 0.036 | 3.89E-06 | 1.34E-04 | rs11761199 | 0.194 | 0.036 | 6.44E-08 | 0.018 | 0.023 | 4.43E-01 | NO | 0.061 | 0.048 | NO | NO |
|  |  |  |  | 7.128607334.128607446 | 0.197 | 0.035 | 3.90E-08 | 1.79E-06 | rs4731541 | 0.254 | 0.035 | 1.07E-12 | 0.362 | 0.028 | 4.63E-39 | YES | 0.063 | 0.037 | NO | NO |
|  |  |  |  | 7.128610202.128610369 | 0.173 | 0.036 | 1.58E-06 | 5.90E-05 | rs10239340 | 0.195 | 0.036 | 5.79E-08 | 0.296 | 0.028 | 4.75E-26 | YES | 0.062 | 0.037 | NO | NO |
|  |  |  |  | 7.128615873.128615989 | 0.118 | 0.036 | 1.04E-03 | 2.03E-02 | rs7808907 | 0.154 | 0.036 | 1.82E-05 | 0.234 | 0.024 | 1.83E-22 | YES | 0.062 | 0.036 | NO | NO |
|  |  |  |  | 7.128641113.128641288 | 0.129 | 0.036 | 3.52E-04 | 7.89E-03 | rs6965542 | 0.155 | 0.036 | 1.64E-05 | 0.227 | 0.028 | 5.22E-16 | YES | 0.062 | 0.036 | NO | NO |
|  |  |  |  | 7.128645070.128645213 | 0.123 | 0.036 | 6.66E-04 | 1.40E-02 | rs3807306 | 0.135 | 0.036 | 1.80E-04 | 0.005 | 0.018 | 7.74E-01 | NO | 0.061 | 0.036 | NO | NO |
|  |  |  |  | 7.128658011.128658211 | 0.114 | 0.036 | 1.66E-03 | 2.99E-02 | rs7807018 | 0.142 | 0.036 | 7.82E-05 | 0.204 | 0.028 | 3.25E-13 | YES | 0.062 | 0.037 | NO | NO |
|  |  |  |  | 7.128694705.128695198 | 0.146 | 0.036 | 5.19E-05 | 1.44E-03 | rs6961014 | 0.209 | 0.035 | 5.31E-09 | 0.218 | 0.034 | 1.53E-10 | YES | 0.062 | 0.037 | NO | NO |
|  |  |  | ***TSPAN33*** | 7.128807614.128808671 | 0.118 | 0.036 | 1.13E-03 | 2.18E-02 | rs4728152 | 0.271 | 0.035 | 2.65E-14 | 0.150 | 0.036 | 3.16E-05 | YES | 0.059 | 0.039 | NO | NO |
| 7q32.1 | **rs10488631** | 7:128594183 | ***ATP6V1F*** | 7.128502880.128503116 | 0.122 | 0.036 | 7.47E-04 | 1.53E-02 | rs11761199 | 0.180 | 0.036 | 5.43E-07 | 0.074 | 0.035 | 3.40E-02 | YES | 0.061 | 0.041 | NO | NO |
|  |  |  |  | 7.128505431.128505898 | 0.122 | 0.036 | 7.18E-04 | 1.48E-02 | rs13246321 | 0.123 | 0.036 | 6.71E-04 | 0.000 | 0.000 | 0.00E+00 | NO | 0.061 | 0.041 | NO | NO |
|  |  |  | ***IMPDH1*** | 7.128036986.128037076 | 0.109 | 0.036 | 2.49E-03 | 4.21E-02 | rs1874332 | 0.113 | 0.036 | 1.67E-03 | 0.133 | 0.035 | 1.59E-04 | YES | 0.043 | 0.040 | NO | NO |
|  |  |  | ***IRF5*** | 7.128577666.128577888 | 0.242 | 0.035 | 1.13E-11 | 8.01E-10 | rs3807306 | 0.421 | 0.033 | 3.48E-34 | 0.139 | 0.035 | 5.88E-05 | YES | 0.068 | 0.068 | NO | NO |
|  |  |  |  | 7.128577991.128578103 | 0.202 | 0.035 | 1.78E-08 | 8.71E-07 | rs4728142 | 0.419 | 0.033 | 6.00E-34 | 0.104 | 0.035 | 2.77E-03 | YES | 0.072 | 0.055 | NO | NO |
|  |  |  |  | 7.128580726.128580929 | 0.108 | 0.036 | 2.72E-03 | 4.53E-02 | rs3807306 | 0.226 | 0.035 | 2.61E-10 | 0.046 | 0.035 | 1.82E-01 | NO | 0.064 | 0.040 | NO | NO |
|  |  |  |  | 7.128582125.128582330 | 0.225 | 0.035 | 3.12E-10 | 2.02E-08 | rs13242262 | 0.501 | 0.031 | 8.14E-50 | 0.318 | 0.035 | 1.19E-19 | YES | 0.094 | 0.060 | NO | NO |
|  |  |  |  | 7.128585899.128586088 | 0.229 | 0.035 | 1.43E-10 | 9.90E-09 | rs3807306 | 0.491 | 0.032 | 1.08E-47 | 0.116 | 0.035 | 8.49E-04 | YES | 0.093 | 0.061 | NO | NO |
|  |  |  |  | 7.128586555.128586996 | 0.173 | 0.036 | 1.56E-06 | 5.89E-05 | rs3807306 | 0.446 | 0.032 | 1.13E-38 | 0.064 | 0.035 | 6.51E-02 | NO | 0.077 | 0.048 | NO | NO |
|  |  |  |  | 7.128587077.128587589 | 0.320 | 0.034 | 1.11E-19 | 1.43E-17 | rs3807306 | 0.451 | 0.032 | 1.24E-39 | 0.214 | 0.034 | 4.64E-10 | YES | 0.074 | 0.111 | YES | NO |
|  |  |  |  | 7.128587783.128588175 | 0.231 | 0.035 | 1.06E-10 | 7.38E-09 | rs13242262 | 0.480 | 0.032 | 2.01E-45 | 0.322 | 0.035 | 4.21E-20 | YES | 0.088 | 0.062 | NO | NO |
|  |  |  |  | 7.128588264.128588382 | 0.249 | 0.035 | 2.71E-12 | 2.13E-10 | rs3807306 | 0.497 | 0.031 | 4.84E-49 | 0.135 | 0.035 | 9.05E-05 | YES | 0.089 | 0.069 | NO | NO |
|  |  |  | ***TNPO3*** | 7.128594948.128595439 | 0.165 | 0.036 | 4.39E-06 | 1.50E-04 | rs11761199 | 0.194 | 0.036 | 6.44E-08 | 0.114 | 0.035 | 1.02E-03 | YES | 0.061 | 0.048 | NO | NO |
| 7p15.1 | **rs849142** | 7:28185891 | ***JAZF1*** | 7.27870192.27872595 | 0.177 | 0.036 | 8.41E-07 | 3.35E-05 | rs1635853 | 0.209 | 0.035 | 5.56E-09 | 0.344 | 0.022 | 7.29E-57 | YES | 0.015 | 0.011 | NO | NO |
| 7p12.2 | **rs4917014** | 7:50305863 | ***IKZF1*** | 7.50467616.50472799 | 0.130 | 0.036 | 2.97E-04 | 6.82E-03 | rs7802443 | 0.195 | 0.036 | 5.43E-08 | 0.091 | 0.035 | 1.05E-02 | YES | 0.054 | 0.030 | NO | NO |
| 8p23.1 | **rs2736340** | 8:11343973 | ***AF131215.2*** | 8.10964720.10967428 | 0.274 | 0.035 | 1.21E-14 | 1.18E-12 | rs4618656 | 0.562 | 0.030 | 5.53E-65 | 0.494 | 0.033 | 1.18E-50 | YES | 0.135 | 0.074 | NO | NO |
|  |  |  |  | 8.10983980.10984581 | 0.110 | 0.036 | 2.30E-03 | 3.95E-02 | rs4841659 | 0.140 | 0.036 | 1.07E-04 | 0.058 | 0.034 | 8.35E-02 | NO | 0.050 | 0.039 | NO | NO |
|  |  |  | ***BLK*** | 8.11351510.11352100 | 0.462 | 0.032 | 9.76E-42 | 3.16E-39 | rs2618473 | 0.471 | 0.032 | 2.11E-43 | 0.000 | 0.000 | 0.00E+00 | NO | 0.050 | 0.399 | YES | YES |
|  |  |  |  | 8.11366659.11367400 | 0.350 | 0.034 | 1.72E-23 | 2.55E-21 | rs2736340 | 0.350 | 0.034 | 1.72E-23 | 0.000 | 0.000 | 0.00E+00 | NO | 0.050 | 0.138 | YES | YES |
|  |  |  |  | 8.11400733.11400856 | 0.481 | 0.032 | 1.51E-45 | 9.80E-43 | rs4840568 | 0.485 | 0.032 | 1.94E-46 | 0.030 | 0.013 | 2.18E-02 | YES | 0.040 | 0.457 | YES | NO |
|  |  |  |  | 8.11403240.11403612 | 0.442 | 0.032 | 5.85E-38 | 1.26E-35 | rs4840568 | 0.447 | 0.032 | 6.84E-39 | 0.027 | 0.013 | 4.39E-02 | YES | 0.050 | 0.327 | YES | NO |
|  |  |  |  | 8.11405541.11405634 | 0.469 | 0.032 | 4.89E-43 | 2.01E-40 | rs4840568 | 0.472 | 0.032 | 1.21E-43 | 0.031 | 0.013 | 2.07E-02 | YES | 0.046 | 0.406 | YES | NO |
|  |  |  |  | 8.11406533.11406631 | 0.471 | 0.032 | 1.86E-43 | 9.64E-41 | rs4840568 | 0.479 | 0.032 | 4.65E-45 | 0.026 | 0.013 | 4.81E-02 | YES | 0.044 | 0.431 | YES | NO |
|  |  |  |  | 8.11407503.11407771 | 0.505 | 0.031 | 7.90E-51 | 1.02E-47 | rs4840568 | 0.515 | 0.031 | 4.25E-53 | 0.027 | 0.013 | 4.25E-02 | YES | 0.035 | 0.576 | YES | NO |
|  |  |  |  | 8.11412252.11412398 | 0.469 | 0.032 | 5.06E-43 | 2.01E-40 | rs4840568 | 0.477 | 0.032 | 1.02E-44 | 0.025 | 0.013 | 5.40E-02 | NO | 0.044 | 0.425 | YES | YES |
|  |  |  |  | 8.11412841.11412993 | 0.494 | 0.031 | 2.71E-48 | 2.81E-45 | rs4840568 | 0.502 | 0.031 | 5.08E-50 | 0.028 | 0.013 | 3.48E-02 | YES | 0.041 | 0.519 | YES | NO |
|  |  |  |  | 8.11414166.11414346 | 0.448 | 0.032 | 6.05E-39 | 1.65E-36 | rs2618473 | 0.455 | 0.032 | 2.21E-40 | 0.000 | 0.000 | 0.00E+00 | NO | 0.050 | 0.346 | YES | YES |
|  |  |  |  | 8.11415471.11415547 | 0.445 | 0.032 | 2.13E-38 | 5.26E-36 | rs4840568 | 0.453 | 0.032 | 4.67E-40 | 0.023 | 0.013 | 7.93E-02 | NO | 0.049 | 0.345 | YES | YES |
|  |  |  |  | 8.11417842.11418961 | 0.485 | 0.032 | 2.45E-46 | 2.11E-43 | rs2618473 | 0.490 | 0.032 | 1.69E-47 | 0.000 | 0.000 | 0.00E+00 | NO | 0.043 | 0.469 | YES | YES |
|  |  |  |  | 8.11420488.11420619 | 0.440 | 0.033 | 1.49E-37 | 2.96E-35 | rs2618473 | 0.448 | 0.032 | 4.47E-39 | 0.000 | 0.000 | 0.00E+00 | NO | 0.050 | 0.326 | YES | YES |
|  |  |  |  | 8.11421412.11422113 | 0.446 | 0.032 | 1.32E-38 | 3.43E-36 | rs2618473 | 0.452 | 0.032 | 9.65E-40 | 0.000 | 0.000 | 0.00E+00 | NO | 0.052 | 0.339 | YES | YES |
|  |  |  | ***FAM167A*** | 8.11278972.11282145 | 0.469 | 0.032 | 3.48E-43 | 1.64E-40 | rs1478901 | 0.472 | 0.032 | 9.76E-44 | 0.000 | 0.000 | 0.00E+00 | NO | 0.042 | 0.401 | YES | YES |
|  |  |  |  | 8.11301540.11302395 | 0.463 | 0.032 | 7.22E-42 | 2.49E-39 | rs2736340 | 0.463 | 0.032 | 7.22E-42 | 0.000 | 0.000 | 0.00E+00 | NO | 0.043 | 0.376 | YES | YES |
|  |  |  | ***FDFT1*** | 8.11683489.11683724 | 0.143 | 0.036 | 7.39E-05 | 1.97E-03 | rs904009 | 0.222 | 0.035 | 5.39E-10 | 0.178 | 0.036 | 6.33E-07 | YES | 0.042 | 0.043 | YES | NO |
|  |  |  |  | 8.11687753.11687929 | 0.118 | 0.036 | 1.09E-03 | 2.12E-02 | rs904009 | 0.179 | 0.036 | 6.27E-07 | 0.146 | 0.036 | 4.29E-05 | YES | 0.041 | 0.040 | NO | NO |
|  |  |  |  | 8.11689027.11689303 | 0.116 | 0.036 | 1.29E-03 | 2.44E-02 | rs1296025 | 0.229 | 0.035 | 1.45E-10 | 0.146 | 0.036 | 4.80E-05 | YES | 0.043 | 0.039 | NO | NO |
|  |  |  | ***RP11-148O21.2*** | 8.11415975.11416256 | 0.454 | 0.032 | 3.63E-40 | 1.04E-37 | rs2618473 | 0.456 | 0.032 | 1.39E-40 | 0.000 | 0.000 | 0.00E+00 | NO | 0.042 | 0.351 | YES | YES |
|  |  |  |  | 8.11416421.11416495 | 0.325 | 0.034 | 2.69E-20 | 3.67E-18 | rs4840568 | 0.333 | 0.034 | 3.31E-21 | 0.016 | 0.013 | 2.30E-01 | NO | 0.051 | 0.121 | YES | YES |
|  |  |  |  | 8.11417293.11417529 | 0.395 | 0.033 | 5.49E-30 | 9.17E-28 | rs4840568 | 0.401 | 0.033 | 5.90E-31 | 0.022 | 0.013 | 9.43E-02 | NO | 0.048 | 0.217 | YES | YES |
|  |  |  | ***RP11-148O21.4*** | 8.11413760.11414170 | 0.353 | 0.034 | 6.24E-24 | 9.50E-22 | rs2618473 | 0.362 | 0.034 | 3.70E-25 | 0.000 | 0.000 | 0.00E+00 | NO | 0.048 | 0.153 | YES | YES |
|  |  |  |  | 8.11415399.11415531 | 0.391 | 0.033 | 2.15E-29 | 3.48E-27 | rs1478901 | 0.394 | 0.033 | 9.67E-30 | 0.000 | 0.000 | 0.00E+00 | NO | 0.050 | 0.197 | YES | YES |
| 10q11.23 | **rs2663052** | 10:50069395 | ***WDFY4*** | 10.50025290.50025454 | 0.128 | 0.036 | 4.00E-04 | 8.80E-03 | rs3747874 | 0.138 | 0.036 | 1.29E-04 | 0.250 | 0.017 | 5.79E-50 | YES | 0.052 | 0.041 | NO | NO |
|  |  |  |  | 10.50031227.50032214 | 0.375 | 0.034 | 6.03E-27 | 9.46E-25 | rs7918931 | 0.494 | 0.031 | 2.18E-48 | 0.165 | 0.034 | 1.57E-06 | YES | 0.069 | 0.060 | NO | NO |
|  |  |  |  | 10.50034716.50034954 | 0.166 | 0.036 | 3.69E-06 | 1.28E-04 | rs12261146 | 0.315 | 0.034 | 4.01E-19 | 0.125 | 0.036 | 5.19E-04 | YES | 0.054 | 0.037 | NO | NO |
|  |  |  |  | 10.50040618.50040754 | 0.114 | 0.036 | 1.58E-03 | 2.90E-02 | rs7918931 | 0.212 | 0.035 | 2.95E-09 | 0.024 | 0.033 | 4.68E-01 | NO | 0.053 | 0.037 | NO | NO |
|  |  |  |  | 10.50098624.50098772 | 0.124 | 0.036 | 5.74E-04 | 1.21E-02 | rs12266359 | 0.200 | 0.035 | 2.50E-08 | 0.084 | 0.036 | 1.88E-02 | YES | 0.053 | 0.036 | NO | NO |
|  |  |  |  | 10.50151375.50151494 | 0.130 | 0.036 | 3.11E-04 | 7.09E-03 | rs3747874 | 0.132 | 0.036 | 2.52E-04 | 0.247 | 0.017 | 4.46E-49 | YES | 0.052 | 0.037 | NO | NO |
|  |  |  |  | 10.50154085.50154202 | 0.112 | 0.036 | 1.91E-03 | 3.41E-02 | rs10776648 | 0.123 | 0.036 | 6.82E-04 | 0.220 | 0.017 | 6.92E-38 | YES | 0.052 | 0.039 | NO | NO |
|  |  |  |  | 10.50171887.50172099 | 0.164 | 0.036 | 5.37E-06 | 1.82E-04 | rs12248218 | 0.225 | 0.035 | 3.10E-10 | 0.068 | 0.033 | 3.92E-02 | YES | 0.053 | 0.038 | NO | NO |
|  |  |  |  | 10.50184842.50185024 | 0.153 | 0.036 | 2.23E-05 | 6.75E-04 | rs7078312 | 0.160 | 0.036 | 8.50E-06 | 0.000 | 0.000 | 0.00E+00 | NO | 0.052 | 0.039 | NO | NO |
|  |  |  |  | 10.50186354.50186438 | 0.133 | 0.036 | 2.16E-04 | 5.13E-03 | rs11101475 | 0.176 | 0.036 | 9.13E-07 | 0.113 | 0.036 | 1.73E-03 | YES | 0.052 | 0.038 | NO | NO |
|  |  |  |  | 10.50189412.50189523 | 0.125 | 0.036 | 5.16E-04 | 1.10E-02 | rs7078312 | 0.140 | 0.036 | 9.76E-05 | 0.000 | 0.000 | 0.00E+00 | NO | 0.052 | 0.039 | NO | NO |
|  |  |  |  | 10.50190554.50191001 | 0.112 | 0.036 | 1.95E-03 | 3.46E-02 | rs12263411 | 0.152 | 0.036 | 2.47E-05 | 0.087 | 0.036 | 1.50E-02 | YES | 0.052 | 0.036 | NO | NO |
| 11p13 | **rs2732549** | 11:35088399 | ***ABTB2*** | 11.34176248.34176316 | 0.114 | 0.036 | 1.59E-03 | 2.91E-02 | rs6484702 | 0.137 | 0.036 | 1.38E-04 | 0.120 | 0.036 | 9.54E-04 | YES | 0.051 | 0.039 | NO | NO |
|  |  |  | ***APIP*** | 11.34912051.34912288 | 0.151 | 0.036 | 2.64E-05 | 7.86E-04 | rs7110461 | 0.535 | 0.031 | 6.29E-58 | 0.043 | 0.036 | 2.25E-01 | NO | 0.120 | 0.041 | NO | NO |
|  |  |  | ***PDHX*** | 11.34978931.34979130 | 0.108 | 0.036 | 2.88E-03 | 4.71E-02 | rs2915236 | 0.209 | 0.035 | 5.07E-09 | 0.145 | 0.036 | 4.78E-05 | YES | 0.067 | 0.040 | NO | NO |
| 11p15.5 | **rs2396545** | 11:601785 | ***ANO9*** | 11.417933.418589 | 0.180 | 0.036 | 5.66E-07 | 2.32E-05 | rs4963176 | 0.355 | 0.034 | 4.32E-24 | 0.024 | 0.032 | 4.61E-01 | NO | 0.020 | 0.051 | YES | YES |
|  |  |  |  | 11.418720.419415 | 0.207 | 0.035 | 7.70E-09 | 3.95E-07 | rs11821341 | 0.353 | 0.034 | 6.99E-24 | 0.034 | 0.031 | 2.79E-01 | NO | 0.020 | 0.057 | YES | YES |
|  |  |  |  | 11.419582.420860 | 0.196 | 0.035 | 4.54E-08 | 2.06E-06 | rs11821341 | 0.362 | 0.034 | 4.20E-25 | 0.019 | 0.031 | 5.46E-01 | NO | 0.020 | 0.053 | YES | YES |
|  |  |  |  | 11.420929.421042 | 0.163 | 0.036 | 6.11E-06 | 2.03E-04 | rs4963176 | 0.308 | 0.034 | 2.99E-18 | 0.027 | 0.032 | 4.06E-01 | NO | 0.019 | 0.046 | YES | YES |
|  |  |  |  | 11.428088.428199 | 0.119 | 0.036 | 9.93E-04 | 1.97E-02 | rs11823647 | 0.241 | 0.035 | 1.52E-11 | -0.001 | 0.031 | 9.87E-01 | NO | 0.018 | 0.040 | YES | YES |
|  |  |  |  | 11.428475.428639 | 0.181 | 0.036 | 4.99E-07 | 2.07E-05 | rs4963176 | 0.338 | 0.034 | 6.34E-22 | 0.032 | 0.032 | 3.27E-01 | NO | 0.020 | 0.050 | YES | YES |
|  |  |  |  | 11.428722.428826 | 0.164 | 0.036 | 5.44E-06 | 1.83E-04 | rs4963176 | 0.311 | 0.034 | 1.14E-18 | 0.026 | 0.032 | 4.19E-01 | NO | 0.019 | 0.048 | YES | YES |
|  |  |  |  | 11.429474.430179 | 0.203 | 0.035 | 1.52E-08 | 7.63E-07 | rs11823647 | 0.353 | 0.034 | 8.03E-24 | 0.030 | 0.031 | 3.40E-01 | NO | 0.020 | 0.058 | YES | YES |
|  |  |  |  | 11.430269.430403 | 0.198 | 0.035 | 3.28E-08 | 1.52E-06 | rs11821341 | 0.331 | 0.034 | 5.74E-21 | 0.036 | 0.031 | 2.53E-01 | NO | 0.019 | 0.051 | YES | YES |
|  |  |  |  | 11.431694.433459 | 0.144 | 0.036 | 6.20E-05 | 1.69E-03 | rs4963176 | 0.300 | 0.035 | 2.11E-17 | 0.012 | 0.032 | 7.19E-01 | NO | 0.019 | 0.044 | YES | YES |
|  |  |  |  | 11.433815.433937 | 0.156 | 0.036 | 1.45E-05 | 4.48E-04 | rs4963176 | 0.253 | 0.035 | 1.31E-12 | 0.044 | 0.032 | 1.73E-01 | NO | 0.019 | 0.045 | YES | YES |
|  |  |  |  | 11.434024.434098 | 0.127 | 0.036 | 4.24E-04 | 9.30E-03 | rs4963176 | 0.229 | 0.035 | 1.59E-10 | 0.026 | 0.032 | 4.28E-01 | NO | 0.019 | 0.042 | YES | YES |
|  |  |  | ***EPS8L2*** | 11.726081.726484 | 0.126 | 0.036 | 4.88E-04 | 1.05E-02 | rs4074376 | 0.167 | 0.036 | 3.26E-06 | 0.098 | 0.036 | 6.02E-03 | YES | 0.021 | 0.041 | YES | NO |
|  |  |  |  | 11.726901.727727 | 0.130 | 0.036 | 3.14E-04 | 7.11E-03 | rs11502190 | 0.210 | 0.035 | 4.32E-09 | 0.085 | 0.035 | 1.57E-02 | YES | 0.021 | 0.041 | YES | NO |
|  |  |  | ***HRAS*** | 11.532242.532755 | 0.160 | 0.036 | 8.65E-06 | 2.80E-04 | rs2613996 | 0.194 | 0.036 | 6.55E-08 | 0.078 | 0.033 | 1.67E-02 | YES | 0.021 | 0.044 | YES | NO |
|  |  |  |  | 11.533277.533358 | 0.136 | 0.036 | 1.64E-04 | 3.97E-03 | rs2246614 | 0.145 | 0.036 | 5.44E-05 | 0.051 | 0.029 | 8.19E-02 | NO | 0.020 | 0.042 | YES | YES |
|  |  |  |  | 11.533453.533612 | 0.142 | 0.036 | 8.44E-05 | 2.18E-03 | rs7944548 | 0.156 | 0.036 | 1.43E-05 | 0.067 | 0.032 | 3.47E-02 | YES | 0.020 | 0.043 | YES | NO |
|  |  |  |  | 11.533766.533976 | 0.151 | 0.036 | 2.72E-05 | 7.97E-04 | rs3740651 | 0.175 | 0.036 | 1.04E-06 | 0.041 | 0.028 | 1.43E-01 | NO | 0.021 | 0.043 | YES | YES |
|  |  |  |  | 11.534212.534375 | 0.138 | 0.036 | 1.29E-04 | 3.24E-03 | rs7117022 | 0.146 | 0.036 | 4.79E-05 | 0.011 | 0.018 | 5.43E-01 | NO | 0.020 | 0.040 | YES | YES |
|  |  |  | ***PHRF1*** | 11.587259.588497 | 0.126 | 0.036 | 4.86E-04 | 1.05E-02 | rs702966 | 0.132 | 0.036 | 2.59E-04 | 0.004 | 0.014 | 7.66E-01 | NO | 0.021 | 0.042 | YES | YES |
|  |  |  |  | 11.597395.597570 | 0.118 | 0.036 | 1.03E-03 | 2.01E-02 | rs702966 | 0.134 | 0.036 | 2.11E-04 | -0.005 | 0.014 | 7.15E-01 | NO | 0.021 | 0.042 | YES | YES |
|  |  |  |  | 11.606442.606842 | 0.155 | 0.036 | 1.73E-05 | 5.31E-04 | rs2740373 | 0.171 | 0.036 | 1.97E-06 | 0.123 | 0.036 | 5.37E-04 | YES | 0.021 | 0.046 | YES | NO |
|  |  |  |  | 11.607066.609720 | 0.170 | 0.036 | 2.37E-06 | 8.56E-05 | rs7936397 | 0.193 | 0.036 | 8.00E-08 | 0.006 | 0.019 | 7.42E-01 | NO | 0.021 | 0.048 | YES | YES |
|  |  |  |  | 11.611634.612222 | 0.142 | 0.036 | 8.00E-05 | 2.09E-03 | rs7936397 | 0.154 | 0.036 | 1.89E-05 | 0.011 | 0.019 | 5.48E-01 | NO | 0.021 | 0.043 | YES | YES |
|  |  |  | ***RASSF7*** | 11.561547.561892 | 0.160 | 0.036 | 9.26E-06 | 2.98E-04 | rs7117022 | 0.166 | 0.036 | 3.84E-06 | 0.015 | 0.018 | 3.93E-01 | NO | 0.021 | 0.043 | YES | YES |
|  |  |  |  | 11.562079.562776 | 0.143 | 0.036 | 7.18E-05 | 1.94E-03 | rs12293143 | 0.160 | 0.036 | 9.22E-06 | 0.152 | 0.036 | 2.38E-05 | YES | 0.021 | 0.042 | YES | NO |
|  |  |  | ***RNH1*** | 11.498457.498627 | 0.152 | 0.036 | 2.58E-05 | 7.72E-04 | rs10540 | 0.264 | 0.035 | 1.10E-13 | 0.065 | 0.034 | 5.78E-02 | NO | 0.020 | 0.043 | YES | YES |
|  |  |  |  | 11.500847.503570 | 0.301 | 0.035 | 1.88E-17 | 2.26E-15 | rs7944548 | 0.508 | 0.031 | 1.88E-51 | 0.064 | 0.032 | 4.32E-02 | YES | 0.031 | 0.079 | YES | NO |
|  |  |  |  | 11.504824.505881 | 0.309 | 0.034 | 2.28E-18 | 2.82E-16 | rs7944548 | 0.560 | 0.030 | 2.37E-64 | 0.050 | 0.032 | 1.16E-01 | NO | 0.048 | 0.104 | YES | YES |
|  |  |  |  | 11.507113.507300 | 0.126 | 0.036 | 4.55E-04 | 9.90E-03 | rs10540 | 0.280 | 0.035 | 2.77E-15 | 0.035 | 0.034 | 3.13E-01 | NO | 0.020 | 0.039 | YES | YES |
|  |  |  | ***RP11-326C3.7*** | 11.310139.310423 | 0.114 | 0.036 | 1.57E-03 | 2.89E-02 | rs10398 | 0.304 | 0.034 | 7.13E-18 | 0.149 | 0.036 | 3.57E-05 | YES | 0.015 | 0.040 | YES | NO |
|  |  |  | ***TMEM80*** | 11.698869.700235 | 0.157 | 0.036 | 1.22E-05 | 3.85E-04 | rs11246240 | 0.279 | 0.035 | 4.16E-15 | 0.075 | 0.035 | 2.94E-02 | YES | 0.022 | 0.046 | YES | NO |
|  |  |  |  | 11.700615.701127 | 0.131 | 0.036 | 2.75E-04 | 6.37E-03 | rs7116631 | 0.259 | 0.035 | 3.50E-13 | 0.058 | 0.035 | 9.26E-02 | NO | 0.021 | 0.046 | YES | YES |
|  |  |  |  | 11.702945.704129 | 0.218 | 0.035 | 1.19E-09 | 6.89E-08 | rs7118663 | 0.380 | 0.033 | 1.03E-27 | 0.332 | 0.034 | 3.14E-22 | YES | 0.023 | 0.068 | YES | NO |
| 11q13.4 | **rs3794060** | 11:71187679 | ***DHCR7*** | 11.71145460.71147019 | 0.246 | 0.035 | 5.41E-12 | 4.00E-10 | rs1792282 | 0.283 | 0.035 | 1.45E-15 | 0.022 | 0.022 | 3.22E-01 | NO | 0.016 | 0.023 | YES | YES |
|  |  |  |  | 11.71148858.71148989 | 0.172 | 0.036 | 1.66E-06 | 6.15E-05 | rs1792282 | 0.215 | 0.035 | 2.01E-09 | 0.003 | 0.022 | 9.05E-01 | NO | 0.016 | 0.018 | YES | YES |
|  |  |  |  | 11.71149795.71150129 | 0.179 | 0.036 | 6.25E-07 | 2.51E-05 | rs1792282 | 0.199 | 0.035 | 2.96E-08 | 0.022 | 0.022 | 3.24E-01 | NO | 0.016 | 0.018 | YES | YES |
|  |  |  |  | 11.71152273.71152486 | 0.163 | 0.036 | 5.77E-06 | 1.93E-04 | rs1792282 | 0.214 | 0.035 | 2.12E-09 | -0.006 | 0.022 | 7.82E-01 | NO | 0.016 | 0.017 | YES | YES |
|  |  |  |  | 11.71153309.71153399 | 0.207 | 0.035 | 7.53E-09 | 3.90E-07 | rs1630498 | 0.235 | 0.035 | 5.05E-11 | 0.013 | 0.021 | 5.19E-01 | NO | 0.016 | 0.020 | YES | YES |
|  |  |  |  | 11.71155003.71155299 | 0.175 | 0.036 | 1.14E-06 | 4.45E-05 | rs1792282 | 0.222 | 0.035 | 5.46E-10 | 0.000 | 0.022 | 9.84E-01 | NO | 0.016 | 0.018 | YES | YES |
|  |  |  |  | 11.71155901.71156004 | 0.150 | 0.036 | 3.12E-05 | 9.03E-04 | rs1792282 | 0.172 | 0.036 | 1.71E-06 | 0.014 | 0.022 | 5.26E-01 | NO | 0.015 | 0.017 | YES | YES |
|  |  |  |  | 11.71158656.71159124 | 0.161 | 0.036 | 7.76E-06 | 2.53E-04 | rs7122671 | 0.201 | 0.035 | 2.20E-08 | 0.129 | 0.036 | 3.10E-04 | YES | 0.016 | 0.017 | YES | NO |
|  |  |  | ***NADSYN1*** | 11.71175099.71175554 | 0.227 | 0.035 | 2.04E-10 | 1.37E-08 | rs7928249 | 0.230 | 0.035 | 1.21E-10 | 0.000 | 0.000 | 0.00E+00 | NO | 0.016 | 0.021 | YES | YES |
|  |  |  |  | 11.71185441.71186668 | 0.545 | 0.030 | 1.79E-60 | 9.26E-57 | rs2282621 | 0.546 | 0.030 | 1.22E-60 | 0.000 | 0.000 | 0.00E+00 | NO | 0.028 | 0.280 | YES | YES |
|  |  |  |  | 11.71187079.71188484 | 0.538 | 0.031 | 1.06E-58 | 2.75E-55 | rs2282621 | 0.539 | 0.030 | 7.82E-59 | 0.000 | 0.000 | 0.00E+00 | NO | 0.027 | 0.259 | YES | YES |
|  |  |  |  | 11.71189441.71190128 | 0.339 | 0.034 | 5.32E-22 | 7.45E-20 | rs2282621 | 0.341 | 0.034 | 3.20E-22 | 0.000 | 0.000 | 0.00E+00 | NO | 0.017 | 0.037 | YES | YES |
|  |  |  |  | 11.71190340.71191320 | 0.419 | 0.033 | 7.54E-34 | 1.30E-31 | rs3750997 | 0.425 | 0.033 | 6.36E-35 | 0.000 | 0.000 | 0.00E+00 | NO | 0.019 | 0.070 | YES | YES |
|  |  |  |  | 11.71191801.71193071 | 0.245 | 0.035 | 6.27E-12 | 4.57E-10 | rs7928249 | 0.247 | 0.035 | 4.38E-12 | 0.000 | 0.000 | 0.00E+00 | NO | 0.016 | 0.023 | YES | YES |
|  |  |  |  | 11.71193769.71194063 | 0.249 | 0.035 | 3.06E-12 | 2.37E-10 | rs4944998 | 0.255 | 0.035 | 8.76E-13 | 0.156 | 0.034 | 4.30E-06 | YES | 0.016 | 0.024 | YES | NO |
|  |  |  |  | 11.71195358.71196694 | 0.280 | 0.035 | 2.94E-15 | 2.98E-13 | rs7928249 | 0.281 | 0.035 | 2.29E-15 | 0.000 | 0.000 | 0.00E+00 | NO | 0.016 | 0.027 | YES | YES |
|  |  |  |  | 11.71201891.71202022 | 0.162 | 0.036 | 6.73E-06 | 2.22E-04 | rs7129099 | 0.168 | 0.036 | 2.94E-06 | 0.134 | 0.036 | 1.72E-04 | YES | 0.016 | 0.017 | YES | NO |
|  |  |  |  | 11.71202880.71202949 | 0.166 | 0.036 | 3.68E-06 | 1.28E-04 | rs4945008 | 0.176 | 0.036 | 9.93E-07 | 0.000 | 0.000 | 0.00E+00 | NO | 0.016 | 0.018 | YES | YES |
|  |  |  |  | 11.71207481.71208657 | 0.248 | 0.035 | 3.30E-12 | 2.51E-10 | rs7928249 | 0.251 | 0.035 | 1.93E-12 | 0.000 | 0.000 | 0.00E+00 | NO | 0.016 | 0.024 | YES | YES |
|  |  |  |  | 11.71209398.71211081 | 0.196 | 0.036 | 4.82E-08 | 2.15E-06 | rs3750997 | 0.199 | 0.035 | 2.91E-08 | 0.000 | 0.000 | 0.00E+00 | NO | 0.016 | 0.020 | YES | YES |
|  |  |  |  | 11.71212348.71212862 | 0.199 | 0.035 | 2.70E-08 | 1.28E-06 | rs4944998 | 0.221 | 0.035 | 6.35E-10 | 0.119 | 0.034 | 4.38E-04 | YES | 0.016 | 0.019 | YES | NO |
|  |  |  |  | 11.71214910.71216920 | 0.297 | 0.035 | 5.05E-17 | 5.81E-15 | rs2282621 | 0.297 | 0.035 | 4.24E-17 | 0.000 | 0.000 | 0.00E+00 | NO | 0.017 | 0.028 | YES | YES |
|  |  |  | ***RP11-660L16.2*** | 11.71159720.71159931 | 0.482 | 0.032 | 7.76E-46 | 5.74E-43 | rs2282621 | 0.484 | 0.032 | 4.45E-46 | 0.000 | 0.000 | 0.00E+00 | NO | 0.022 | 0.136 | YES | YES |
|  |  |  |  | 11.71162736.71163203 | 0.530 | 0.031 | 1.44E-56 | 2.49E-53 | rs7928249 | 0.530 | 0.031 | 1.25E-56 | 0.000 | 0.000 | 0.00E+00 | NO | 0.027 | 0.238 | YES | YES |
| 12q24.12 | **rs10774625** | 12:111910219 | ***AC003029.1*** | 12.112277776.112278281 | 0.117 | 0.036 | 1.21E-03 | 2.30E-02 | rs1544396 | 0.218 | 0.035 | 1.19E-09 | 0.182 | 0.035 | 1.49E-07 | YES | 0.001 | 0.003 | YES | NO |
|  |  |  | ***HECTD4*** | 12.112601913.112602079 | 0.113 | 0.036 | 1.81E-03 | 3.25E-02 | rs11066301 | 0.123 | 0.036 | 6.53E-04 | 0.015 | 0.022 | 5.01E-01 | NO | 0.000 | 0.002 | YES | YES |
|  |  |  | ***NAA25*** | 12.112481429.112481672 | 0.122 | 0.036 | 7.56E-04 | 1.53E-02 | rs4357753 | 0.192 | 0.036 | 8.92E-08 | 0.149 | 0.036 | 3.50E-05 | YES | 0.001 | 0.002 | YES | NO |
|  |  |  | ***SH2B3*** | 12.111884746.111884837 | 0.135 | 0.036 | 1.82E-04 | 4.37E-03 | rs3847953 | 0.160 | 0.036 | 8.50E-06 | 0.199 | 0.033 | 2.27E-09 | YES | 0.002 | 0.003 | YES | NO |
|  |  |  |  | 12.111885787.111889427 | 0.145 | 0.036 | 5.85E-05 | 1.61E-03 | rs7312913 | 0.185 | 0.036 | 2.56E-07 | 0.209 | 0.034 | 1.14E-09 | YES | 0.002 | 0.004 | YES | NO |
| 12q24.32 | **rs1059312** | 12:129278864 | ***SLC15A4*** | 12.129277739.129278901 | 0.300 | 0.035 | 2.45E-17 | 2.88E-15 | rs2291349 | 0.304 | 0.034 | 8.31E-18 | 0.000 | 0.000 | 0.00E+00 | NO | 0.064 | 0.047 | NO | NO |
|  |  |  |  | 12.129283804.129283962 | 0.266 | 0.035 | 7.95E-14 | 7.49E-12 | rs4760593 | 0.269 | 0.035 | 3.75E-14 | 0.000 | 0.000 | 0.00E+00 | NO | 0.064 | 0.042 | NO | NO |
|  |  |  |  | 12.129285352.129286246 | 0.256 | 0.035 | 6.90E-13 | 5.85E-11 | rs4760593 | 0.260 | 0.035 | 2.73E-13 | 0.000 | 0.000 | 0.00E+00 | NO | 0.064 | 0.044 | NO | NO |
|  |  |  |  | 12.129293333.129293501 | 0.122 | 0.036 | 7.31E-04 | 1.50E-02 | rs9738836 | 0.142 | 0.036 | 7.68E-05 | 0.200 | 0.030 | 3.30E-11 | YES | 0.063 | 0.035 | NO | NO |
|  |  |  |  | 12.129293941.129294903 | 0.272 | 0.035 | 2.08E-14 | 2.00E-12 | rs12370194 | 0.274 | 0.035 | 1.32E-14 | 0.000 | 0.000 | 0.00E+00 | NO | 0.064 | 0.042 | NO | NO |
|  |  |  |  | 12.129299320.129300473 | 0.182 | 0.036 | 4.18E-07 | 1.74E-05 | rs3825181 | 0.291 | 0.035 | 1.94E-16 | -0.028 | 0.025 | 2.68E-01 | NO | 0.064 | 0.042 | NO | NO |
|  |  |  |  | 12.129304073.129304412 | 0.174 | 0.036 | 1.34E-06 | 5.11E-05 | rs7308691 | 0.220 | 0.035 | 7.22E-10 | 0.291 | 0.031 | 2.57E-21 | YES | 0.064 | 0.036 | NO | NO |
|  |  |  |  | 12.129307943.129308528 | 0.114 | 0.036 | 1.59E-03 | 2.91E-02 | rs9738836 | 0.152 | 0.036 | 2.51E-05 | 0.198 | 0.030 | 6.27E-11 | YES | 0.063 | 0.035 | NO | NO |
| 15q24.2 | **rs2289583** | 15:75311036 | ***C15orf39*** | 15.75494223.75494400 | 0.109 | 0.036 | 2.48E-03 | 4.21E-02 | rs16966775 | 0.196 | 0.036 | 4.70E-08 | 0.125 | 0.036 | 5.13E-04 | YES | 0.013 | 0.020 | YES | NO |
|  |  |  |  | 15.75498340.75501165 | 0.199 | 0.035 | 3.02E-08 | 1.41E-06 | rs12905302 | 0.289 | 0.035 | 3.36E-16 | 0.227 | 0.036 | 3.62E-10 | YES | 0.013 | 0.022 | YES | NO |
|  |  |  | ***CSK*** | 15.75090574.75090653 | 0.153 | 0.036 | 2.27E-05 | 6.82E-04 | rs1378941 | 0.233 | 0.035 | 7.44E-11 | 0.032 | 0.031 | 2.95E-01 | NO | 0.013 | 0.032 | YES | YES |
|  |  |  |  | 15.75091613.75091832 | 0.140 | 0.036 | 9.68E-05 | 2.48E-03 | rs1378942 | 0.236 | 0.035 | 4.20E-11 | 0.020 | 0.031 | 5.13E-01 | NO | 0.013 | 0.031 | YES | YES |
|  |  |  |  | 15.75092753.75092846 | 0.143 | 0.036 | 7.38E-05 | 1.97E-03 | rs6495122 | 0.252 | 0.035 | 1.57E-12 | 0.030 | 0.032 | 3.54E-01 | NO | 0.014 | 0.032 | YES | YES |
|  |  |  |  | 15.75093164.75093263 | 0.117 | 0.036 | 1.15E-03 | 2.20E-02 | rs1378940 | 0.272 | 0.035 | 2.01E-14 | -0.022 | 0.031 | 4.79E-01 | NO | 0.014 | 0.036 | YES | YES |
|  |  |  |  | 15.75093863.75093936 | 0.140 | 0.036 | 1.08E-04 | 2.76E-03 | rs1378940 | 0.179 | 0.036 | 6.23E-07 | 0.047 | 0.031 | 1.32E-01 | NO | 0.013 | 0.026 | YES | YES |
|  |  |  |  | 15.75094036.75094231 | 0.167 | 0.036 | 3.20E-06 | 1.14E-04 | rs1378940 | 0.252 | 0.035 | 1.68E-12 | 0.038 | 0.031 | 2.22E-01 | NO | 0.014 | 0.035 | YES | YES |
|  |  |  |  | 15.75094338.75094424 | 0.129 | 0.036 | 3.65E-04 | 8.10E-03 | rs1378941 | 0.201 | 0.035 | 1.92E-08 | 0.024 | 0.031 | 4.33E-01 | NO | 0.013 | 0.028 | YES | YES |
|  |  |  |  | 15.75094672.75095539 | 0.208 | 0.035 | 5.84E-09 | 3.08E-07 | rs1378940 | 0.280 | 0.035 | 3.33E-15 | 0.065 | 0.031 | 3.71E-02 | YES | 0.014 | 0.040 | YES | NO |
|  |  |  | ***FAM219B*** | 15.75192329.75195127 | 0.148 | 0.036 | 4.08E-05 | 1.15E-03 | rs8042694 | 0.195 | 0.036 | 5.13E-08 | 0.013 | 0.026 | 6.24E-01 | NO | 0.014 | 0.023 | YES | YES |
|  |  |  | ***MPI*** | 15.75182867.75182995 | 0.150 | 0.036 | 3.29E-05 | 9.41E-04 | rs3765066 | 0.284 | 0.035 | 1.25E-15 | -0.008 | 0.030 | 7.82E-01 | NO | 0.015 | 0.032 | YES | YES |
|  |  |  |  | 15.75183720.75183920 | 0.264 | 0.035 | 1.16E-13 | 1.05E-11 | rs11072512 | 0.409 | 0.033 | 2.89E-32 | 0.081 | 0.032 | 1.22E-02 | YES | 0.018 | 0.054 | YES | NO |
|  |  |  |  | 15.75185002.75185143 | 0.250 | 0.035 | 2.32E-12 | 1.87E-10 | rs4886636 | 0.420 | 0.033 | 5.17E-34 | 0.063 | 0.032 | 5.11E-02 | NO | 0.018 | 0.051 | YES | YES |
|  |  |  |  | 15.75185479.75185661 | 0.191 | 0.036 | 1.08E-07 | 4.75E-06 | rs4886636 | 0.378 | 0.034 | 2.54E-27 | 0.020 | 0.032 | 5.38E-01 | NO | 0.016 | 0.042 | YES | YES |
|  |  |  |  | 15.75188493.75188666 | 0.224 | 0.035 | 3.79E-10 | 2.43E-08 | rs4886636 | 0.350 | 0.034 | 1.98E-23 | 0.064 | 0.032 | 4.74E-02 | YES | 0.016 | 0.037 | YES | NO |
|  |  |  |  | 15.75189352.75189560 | 0.212 | 0.035 | 3.48E-09 | 1.92E-07 | rs11072511 | 0.364 | 0.034 | 1.89E-25 | 0.051 | 0.032 | 1.17E-01 | NO | 0.016 | 0.036 | YES | YES |
|  |  |  |  | 15.75189853.75191798 | 0.262 | 0.035 | 1.81E-13 | 1.56E-11 | rs4886636 | 0.449 | 0.032 | 2.75E-39 | 0.065 | 0.032 | 4.53E-02 | YES | 0.019 | 0.052 | YES | NO |
|  |  |  | ***PPCDC*** | 15.75340894.75341062 | 0.158 | 0.036 | 1.19E-05 | 3.77E-04 | rs8042558 | 0.295 | 0.035 | 8.42E-17 | 0.228 | 0.035 | 1.03E-10 | YES | 0.015 | 0.021 | YES | NO |
|  |  |  | ***ULK3*** | 15.75128459.75129585 | 0.286 | 0.035 | 7.98E-16 | 8.61E-14 | rs9210 | 0.464 | 0.032 | 4.27E-42 | 0.009 | 0.029 | 7.59E-01 | NO | 0.020 | 0.105 | YES | YES |
|  |  |  |  | 15.75130607.75130685 | 0.130 | 0.036 | 3.14E-04 | 7.11E-03 | rs936230 | 0.200 | 0.035 | 2.47E-08 | 0.013 | 0.029 | 6.57E-01 | NO | 0.013 | 0.027 | YES | YES |
|  |  |  |  | 15.75130984.75131086 | 0.142 | 0.036 | 7.90E-05 | 2.09E-03 | rs2301249 | 0.256 | 0.035 | 6.17E-13 | 0.002 | 0.030 | 9.36E-01 | NO | 0.014 | 0.032 | YES | YES |
|  |  |  |  | 15.75131351.75131391 | 0.145 | 0.036 | 5.99E-05 | 1.64E-03 | rs8031937 | 0.194 | 0.036 | 6.87E-08 | 0.013 | 0.027 | 6.25E-01 | NO | 0.014 | 0.026 | YES | YES |
|  |  |  |  | 15.75131609.75131714 | 0.182 | 0.036 | 4.02E-07 | 1.69E-05 | rs7176022 | 0.278 | 0.035 | 4.39E-15 | 0.026 | 0.030 | 3.86E-01 | NO | 0.014 | 0.033 | YES | YES |
|  |  |  |  | 15.75131899.75132054 | 0.150 | 0.036 | 3.16E-05 | 9.08E-04 | rs9210 | 0.236 | 0.035 | 3.89E-11 | 0.009 | 0.029 | 7.52E-01 | NO | 0.014 | 0.028 | YES | YES |
|  |  |  |  | 15.75132575.75132657 | 0.120 | 0.036 | 8.45E-04 | 1.70E-02 | rs9210 | 0.207 | 0.035 | 8.15E-09 | -0.003 | 0.029 | 9.25E-01 | NO | 0.014 | 0.027 | YES | YES |
|  |  |  |  | 15.75132839.75132982 | 0.133 | 0.036 | 2.32E-04 | 5.48E-03 | rs9210 | 0.238 | 0.035 | 2.64E-11 | -0.009 | 0.029 | 7.53E-01 | NO | 0.014 | 0.026 | YES | YES |
|  |  |  |  | 15.75133746.75133850 | 0.116 | 0.036 | 1.35E-03 | 2.54E-02 | rs9210 | 0.215 | 0.035 | 1.81E-09 | -0.013 | 0.029 | 6.63E-01 | NO | 0.014 | 0.026 | YES | YES |
|  |  |  |  | 15.75134416.75134536 | 0.129 | 0.036 | 3.46E-04 | 7.79E-03 | rs936229 | 0.304 | 0.034 | 7.22E-18 | -0.028 | 0.031 | 3.74E-01 | NO | 0.014 | 0.034 | YES | YES |
|  |  |  |  | 15.75134621.75134761 | 0.130 | 0.036 | 3.00E-04 | 6.88E-03 | rs936229 | 0.286 | 0.035 | 7.35E-16 | -0.017 | 0.031 | 5.89E-01 | NO | 0.014 | 0.030 | YES | YES |
| 16p13.13 | **rs9652601** | 16:11174365 | ***CLEC16A*** | 16.11096931.11097162 | 0.109 | 0.036 | 2.44E-03 | 4.16E-02 | rs12935657 | 0.134 | 0.036 | 1.95E-04 | 0.037 | 0.030 | 2.26E-01 | NO | 0.049 | 0.037 | NO | NO |
|  |  |  |  | 16.11214472.11214623 | 0.114 | 0.036 | 1.63E-03 | 2.95E-02 | rs17684919 | 0.165 | 0.036 | 4.52E-06 | 0.129 | 0.036 | 3.61E-04 | YES | 0.050 | 0.036 | NO | NO |
|  |  |  |  | 16.11217599.11217803 | 0.117 | 0.036 | 1.16E-03 | 2.21E-02 | rs7203459 | 0.166 | 0.036 | 3.80E-06 | 0.029 | 0.031 | 3.51E-01 | NO | 0.050 | 0.039 | NO | NO |
|  |  |  |  | 16.11219836.11220201 | 0.195 | 0.036 | 5.83E-08 | 2.58E-06 | rs7203459 | 0.253 | 0.035 | 1.28E-12 | 0.060 | 0.031 | 5.22E-02 | NO | 0.051 | 0.045 | NO | NO |
|  |  |  | ***DEXI*** | 16.11035426.11036317 | 0.148 | 0.036 | 3.92E-05 | 1.11E-03 | rs17804470 | 0.261 | 0.035 | 2.33E-13 | -0.027 | 0.027 | 3.03E-01 | NO | 0.051 | 0.046 | NO | NO |
| 16p11.2 | **rs9936831** | 16:31287148 | ***BCKDK*** | 16.31120832.31120900 | 0.131 | 0.036 | 2.70E-04 | 6.27E-03 | rs889555 | 0.158 | 0.036 | 1.15E-05 | 0.137 | 0.036 | 1.45E-04 | YES | 0.013 | 0.043 | YES | NO |
| 16q24.1 | **rs11644034** | 16:85972612 | ***GSE1*** | 16.85694752.85695371 | 0.111 | 0.036 | 2.02E-03 | 3.53E-02 | rs13330932 | 0.133 | 0.036 | 2.17E-04 | 0.013 | 0.024 | 5.94E-01 | NO | 0.055 | 0.040 | NO | NO |
|  |  |  | ***IRF8*** | 16.85936621.85936795 | 0.196 | 0.036 | 4.62E-08 | 2.08E-06 | rs1401884 | 0.240 | 0.035 | 1.60E-11 | 0.138 | 0.035 | 8.75E-05 | YES | 0.064 | 0.055 | NO | NO |
|  |  |  |  | 16.85942596.85942779 | 0.225 | 0.035 | 2.95E-10 | 1.94E-08 | rs1401884 | 0.298 | 0.035 | 3.70E-17 | 0.153 | 0.035 | 1.32E-05 | YES | 0.064 | 0.063 | NO | NO |
|  |  |  |  | 16.85945176.85945264 | 0.210 | 0.035 | 4.17E-09 | 2.25E-07 | rs12444221 | 0.223 | 0.035 | 4.80E-10 | 0.004 | 0.014 | 7.63E-01 | NO | 0.064 | 0.060 | NO | NO |
|  |  |  |  | 16.85946737.85946842 | 0.202 | 0.035 | 1.78E-08 | 8.71E-07 | rs1401884 | 0.221 | 0.035 | 6.24E-10 | 0.148 | 0.035 | 2.42E-05 | YES | 0.064 | 0.056 | NO | NO |
|  |  |  |  | 16.85952023.85952409 | 0.204 | 0.035 | 1.35E-08 | 6.83E-07 | rs1401884 | 0.254 | 0.035 | 9.41E-13 | 0.142 | 0.035 | 5.30E-05 | YES | 0.064 | 0.055 | NO | NO |
|  |  |  |  | 16.85953715.85953830 | 0.171 | 0.036 | 2.03E-06 | 7.45E-05 | rs1401884 | 0.234 | 0.035 | 5.85E-11 | 0.114 | 0.035 | 1.15E-03 | YES | 0.064 | 0.048 | NO | NO |
|  |  |  |  | 16.85954712.85956197 | 0.219 | 0.035 | 1.00E-09 | 5.96E-08 | rs1401884 | 0.324 | 0.034 | 3.31E-20 | 0.140 | 0.035 | 6.74E-05 | YES | 0.064 | 0.058 | NO | NO |
| 17p13.2 | **rs2286672** | 17:4712617 | ***PSMB6*** | 17.4700733.4700864 | 0.151 | 0.036 | 2.67E-05 | 7.87E-04 | rs2302327 | 0.152 | 0.036 | 2.57E-05 | 0.031 | 0.022 | 1.60E-01 | NO | 0.005 | 0.005 | NO | NO |
|  |  |  |  | 17.4700977.4701106 | 0.141 | 0.036 | 9.06E-05 | 2.33E-03 | rs2302327 | 0.145 | 0.036 | 5.55E-05 | 0.026 | 0.022 | 2.43E-01 | NO | 0.005 | 0.005 | NO | NO |
|  |  |  | ***SLC25A11*** | 17.4843111.4843463 | 0.136 | 0.036 | 1.58E-04 | 3.86E-03 | rs377623 | 0.146 | 0.036 | 5.12E-05 | 0.146 | 0.036 | 5.05E-05 | YES | 0.005 | 0.005 | NO | NO |
| 22q11.21 | **rs7444** | 22:21976934 | ***PPIL2*** | 22.22049226.22052565 | 0.120 | 0.036 | 9.05E-04 | 1.80E-02 | rs12484060 | 0.560 | 0.030 | 2.75E-64 | 0.078 | 0.036 | 2.95E-02 | YES | 0.055 | 0.042 | NO | NO |
|  |  |  | ***UBE2L3*** | 22.21947150.21947245 | 0.169 | 0.036 | 2.55E-06 | 9.09E-05 | rs5754467 | 0.184 | 0.036 | 3.21E-07 | 0.063 | 0.030 | 3.23E-02 | YES | 0.034 | 0.051 | YES | NO |
|  |  |  |  | 22.21965146.21965332 | 0.216 | 0.035 | 1.65E-09 | 9.46E-08 | rs2266959 | 0.221 | 0.035 | 6.83E-10 | 0.000 | 0.000 | 0.00E+00 | NO | 0.034 | 0.062 | YES | YES |
|  |  |  |  | 22.21975804.21978323 | 0.422 | 0.033 | 2.19E-34 | 4.20E-32 | rs4820091 | 0.424 | 0.033 | 9.23E-35 | 0.000 | 0.000 | 0.00E+00 | NO | 0.034 | 0.265 | YES | YES |

**Supplementary Material, Figure S1:** A: Number of eQTL discoveries per quantification type, B: Number of eGene discoveries per quantification type, C: Shared candidate-causal eQTLs per quantification type, D: Shared candidate causal eGenes per quantification type, E: Ratio of eGenes to candidate-causal eQTLs


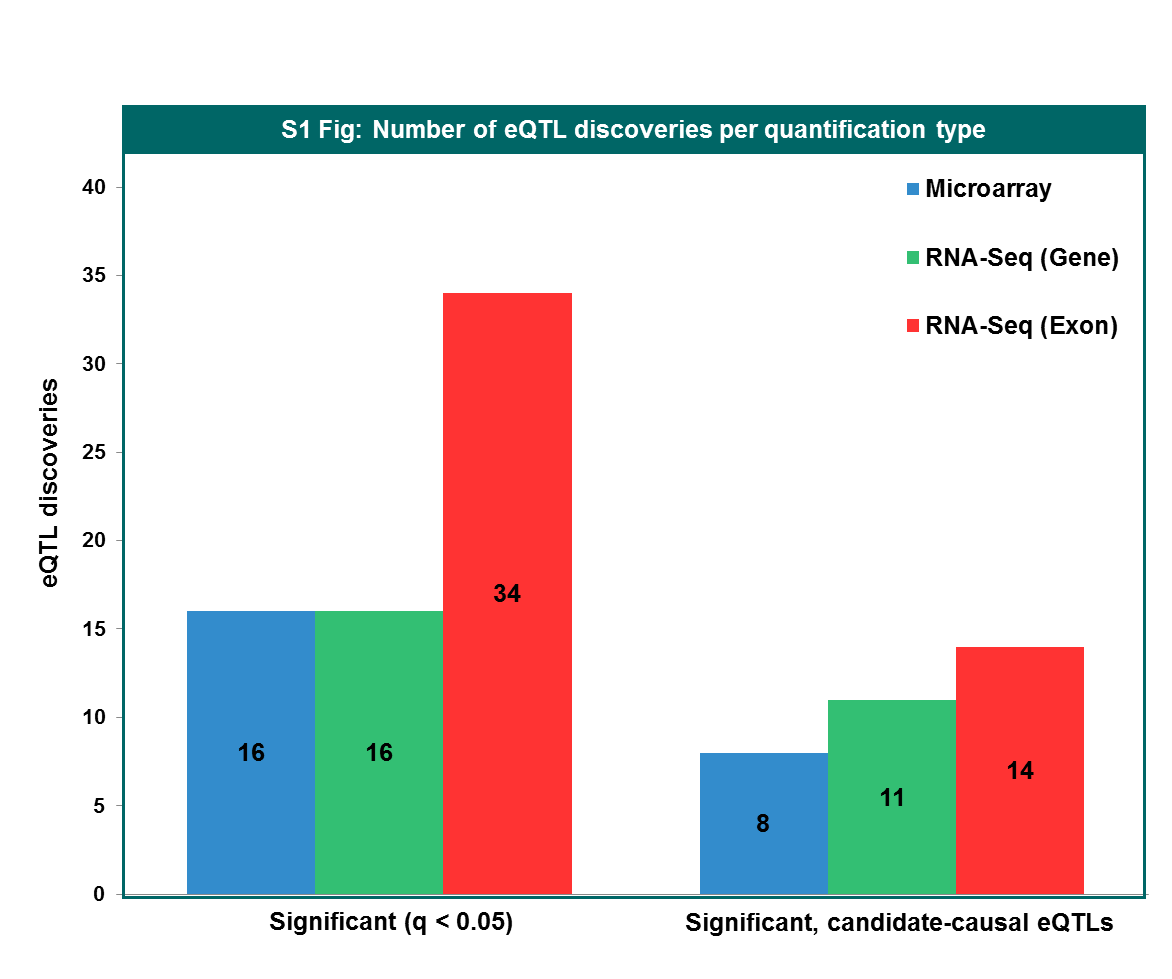

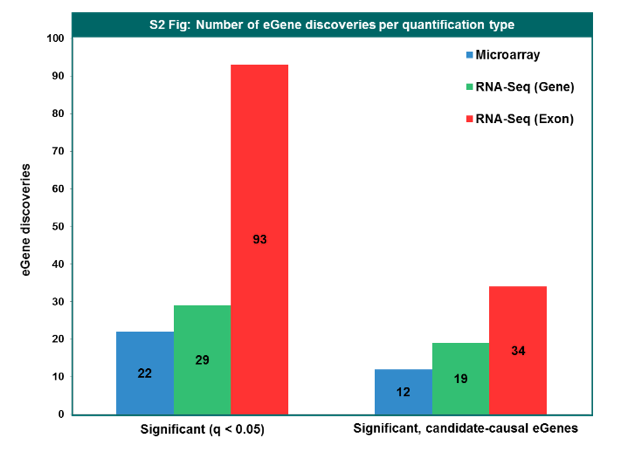

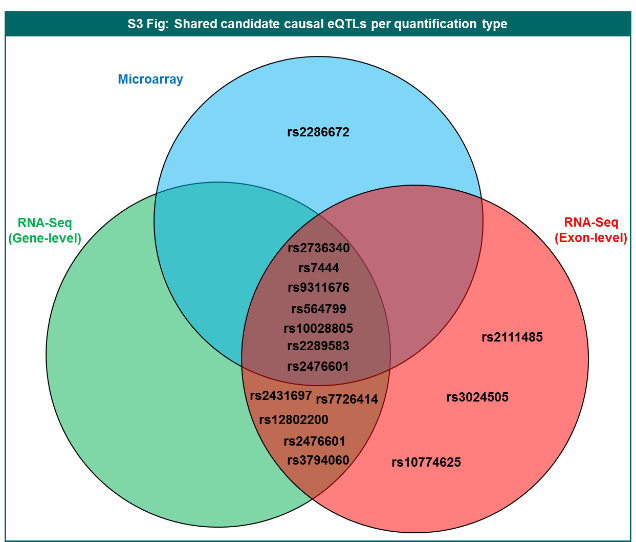

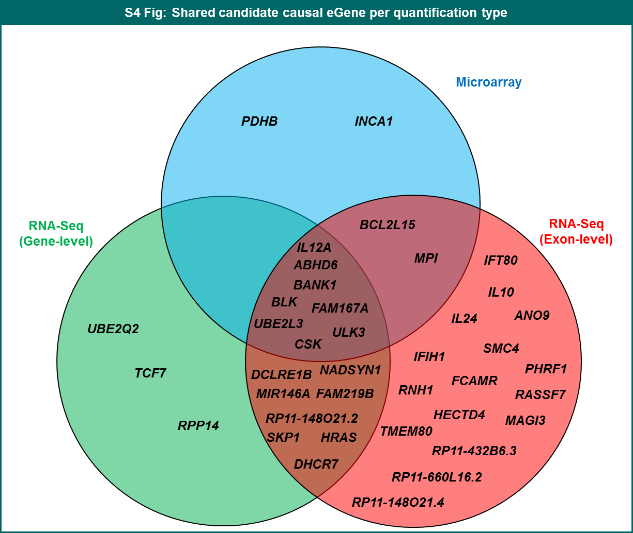

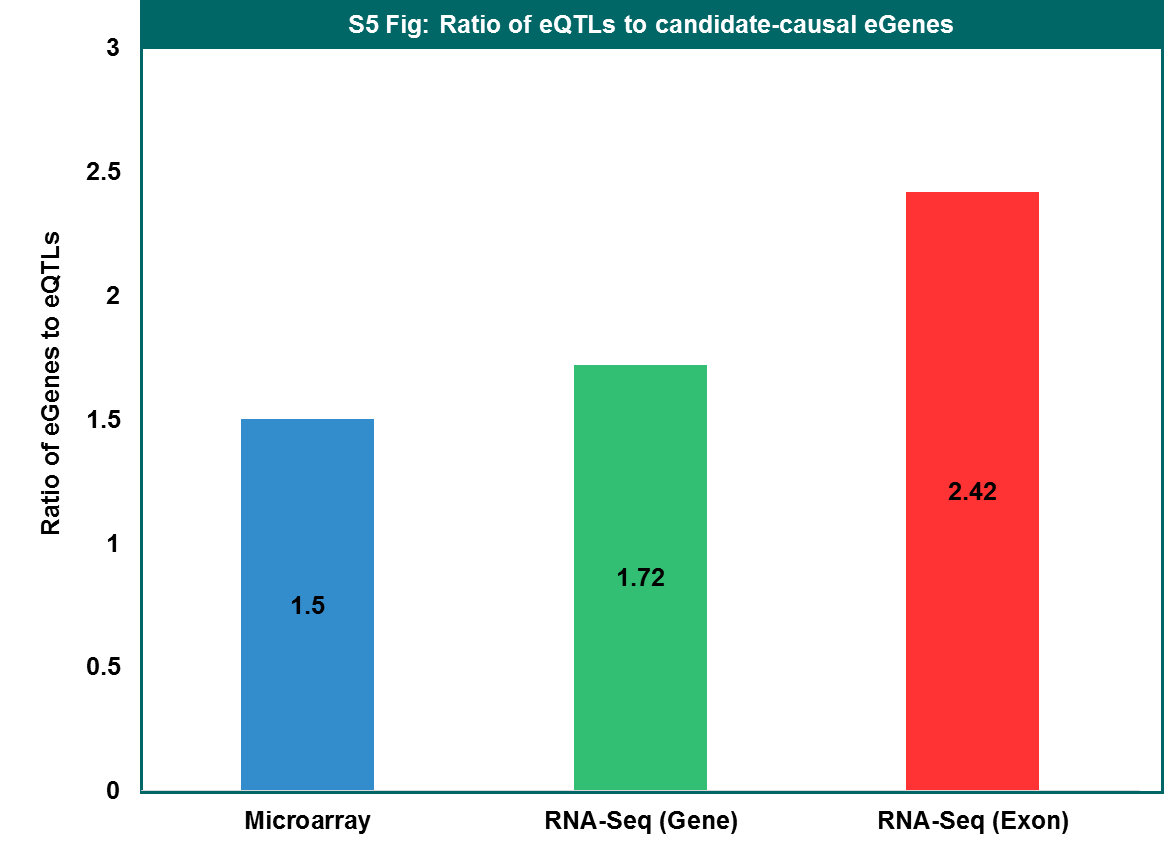


| **Supplementary Material, Table S5**  Exon-level candidate-causal eQTLs and corresponding eGenes mapped back to specific transcript isoforms using unique portion of meta-exon | | | | | | |
| --- | --- | --- | --- | --- | --- | --- |
| **eQTL** | **eGene** | **Number of meta-exons in total for gene** | **Number of meta-exons with eQTL** | **Number of Isoforms in total for gene** | **Number of Isoforms with meta-exon eQTL** | **Single transcript** |
| rs2476601 | *BCL2L15* | 2 | 2 | 6 | 6 |  |
| rs2476601 | *DCLRE1B* | 4 | 1 | 2 | 1 | ENST00000369563 |
| rs2476601 | *MAGI3* | 1 | 1 | 6 | 2 |  |
| rs3024505 | *FCAMR* | 2 | 1 | 7 | 5 |  |
| rs3024505 | *IL10* | 4 | 1 | 3 | 1 | ENST00000423557 |
| rs3024505 | *IL24* | 1 | 1 | 6 | 5 |  |
| rs2111485 | *IFIH1* | 16 | 6 | 3 | 2 |  |
| rs564799 | *IFT80* | 18 | 1 | 27 | 5 |  |
| rs564799 | *IL12A* | 4 | 3 | 6 | 6 |  |
| rs564799 | *RP11-432B6.3* | 15 | 1 | 1 | 1 | ENST00000483754 |
| rs564799 | *SMC4* | 23 | 7 | 28 | 18 |  |
| rs11714389 | *ABHD6* | 8 | 8 | 7 | 5 |  |
| rs10028805 | *BANK1* | 10 | 2 | 8 | 5 |  |
| rs17167273 | *SKP1* | 8 | 1 | 13 | 10 |  |
| rs2431697 | *MIR146A* | 2 | 2 | 2 | 1 | ENST00000517927 |
| rs2736340 | *BLK* | 14 | 8 | 6 | 6 |  |
| rs2736340 | *FAM167A* | 2 | 2 | 7 | 5 |  |
| rs2736340 | *RP11-148O21.2* | 3 | 1 | 1 | 1 | ENST00000533322 |
| rs2736340 | *RP11-148O21.4* | 2 | 1 | 1 | 1 | ENST00000528629 |
| rs2396545 | *ANO9* | 15 | 12 | 8 | 8 |  |
| rs2396545 | *HRAS* | 6 | 3 | 11 | 8 |  |
| rs2396545 | *PHRF1* | 17 | 4 | 6 | 5 |  |
| rs2396545 | *RASSF7* | 3 | 1 | 9 | 7 |  |
| rs2396545 | *RNH1* | 10 | 3 | 24 | 17 |  |
| rs2396545 | *TMEM80* | 3 | 1 | 7 | 6 |  |
| rs2396545 | *DHCR7* | 9 | 7 | 12 | 12 |  |
| rs2396545 | *NADSYN1* | 25 | 11 | 23 | 16 |  |
| rs2396545 | *RP11-660L16.2* | 2 | 1 | 1 | 1 | ENST00000529369 |
| rs10774625 | *HECTD4* | 66 | 1 | 15 | 4 |  |
| rs2289583 | *CSK* | 13 | 7 | 15 | 11 |  |
| rs2289583 | *FAM219B* | 6 | 1 | 20 | 20 |  |
| rs2289583 | *MPI* | 7 | 4 | 23 | 21 |  |
| rs2289583 | *ULK3* | 16 | 11 | 22 | 22 |  |
| rs7444 | *UBE2L3* | 3 | 2 | 4 | 4 |  |

**Supplementary Material, Figure S2:** Effect-size correlation of eQTL associations with matched cis-exons between LCL and whole blood (5,265 meta-exons ~ SNP association pairs)


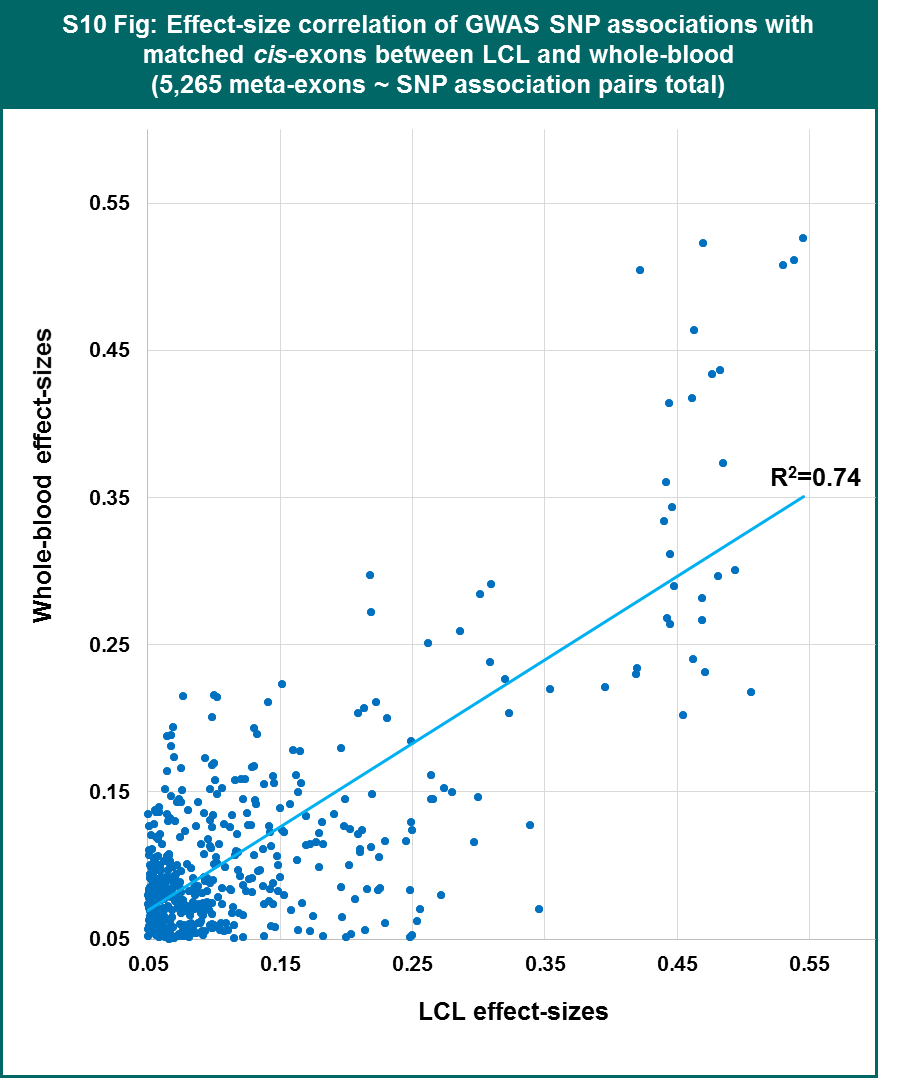


| **Supplementary Material, Table S6**  All significant eQTLs (q < 0.05) and associated eGenes detected at RNA-Seq (exon-level) l with conditional and colocalisation results in whole-blood | | | | | | | | | | | | | | | | | | | | |
| --- | --- | --- | --- | --- | --- | --- | --- | --- | --- | --- | --- | --- | --- | --- | --- | --- | --- | --- | --- | --- |
| **Risk**  **Locus** | **GWAS SNP** | **Coordinate (hg19)** | **eGene** | **Exon ID (chr. start. end)** | **β** | **Std Err** | **P-Value** | **FDR q** | **Best**  **cis eQTL** | **β** | **Std Err** | **P-Value** | **β _cond_** | **Std Err _cond_** | **P-Value _cond_** | **Independent to best** | **PP3** | **PP4** | **Shared Causal Variant** | **Candidate Causal eQTL/eGene** |
| 1q23.3 | **rs1801274** | 1.161479745 | ***FCGR2A*** | 1.161489379.161489503 | 0.186 | 0.051 | 3.34E-04 | 2.34E-02 | rs11582349 | 0.214 | 0.051 | 3.63E-05 | 0.153 | 0.052 | 3.31E-03 | YES | 0.126 | 0.052 | NO | NO |
|  |  |  |  | 1.161489592.161489781 | 0.183 | 0.051 | 4.08E-04 | 2.71E-02 | rs11582349 | 0.265 | 0.050 | 2.45E-07 | 0.142 | 0.052 | 6.30E-03 | YES | 0.127 | 0.051 | NO | NO |
| 1q32.1 | **rs3024505** | 1.206939904 | ***FAIM3*** | 1.207085075.207085297 | 0.189 | 0.051 | 2.67E-04 | 1.97E-02 | rs1856746 | 0.196 | 0.051 | 1.59E-04 | 0.156 | 0.051 | 2.34E-03 | YES | 0.003 | 0.004 | YES | NO |
|  |  |  |  | 1.207086274.207086387 | 0.174 | 0.051 | 8.30E-04 | 4.95E-02 | rs188334 | 0.185 | 0.051 | 3.62E-04 | 0.131 | 0.051 | 9.61E-03 | YES | 0.003 | 0.004 | YES | NO |
|  |  |  |  | 1.207086977.207087439 | 0.218 | 0.051 | 2.39E-05 | 2.44E-03 | rs483850 | 0.283 | 0.050 | 3.17E-08 | 0.163 | 0.051 | 1.28E-03 | YES | 0.003 | 0.004 | YES | NO |
| 3p14.3 | **rs11714389** | 3.58447299 | ***PXK*** | 3.58383332.58383449 | 0.272 | 0.050 | 1.15E-07 | 2.03E-05 | rs4681679 | 0.272 | 0.050 | 1.11E-07 | 0.000 | 0.000 | 0.00E+00 | NO | 0.037 | 0.076 | YES | YES |
|  |  |  |  | 3.58410479.58411748 | 0.292 | 0.050 | 1.21E-08 | 2.58E-06 | rs11130633 | 0.404 | 0.048 | 7.32E-16 | 0.527 | 0.043 | 5.66E-35 | YES | 0.040 | 0.083 | YES | NO |
|  |  |  | ***RPP14*** | 3.58303167.58305816 | 0.207 | 0.051 | 6.18E-05 | 5.45E-03 | rs6445969 | 0.216 | 0.051 | 2.88E-05 | 0.037 | 0.032 | 2.58E-01 | NO | 0.035 | 0.062 | YES | YES |
|  |  |  |  | 3.58303167.58305920 | 0.211 | 0.051 | 4.52E-05 | 4.07E-03 | rs6445969 | 0.216 | 0.051 | 2.86E-05 | 0.040 | 0.032 | 2.14E-01 | NO | 0.035 | 0.062 | YES | YES |
| 4q24 | **rs10028805** | 4.10273725 | ***BANK1*** | 4.102750965.102751363 | 0.204 | 0.051 | 8.41E-05 | 7.11E-03 | rs4411998 | 0.208 | 0.051 | 5.99E-05 | 0.000 | 0.000 | 0.00E+00 | NO | 0.040 | 0.058 | YES | YES |
| 5q31.1 | **rs17167273** | 5.133459347 | ***TXNDC15*** | 5.134223385.134223872 | 0.187 | 0.051 | 3.19E-04 | 2.27E-02 | rs6899047 | 0.213 | 0.051 | 3.86E-05 | 0.107 | 0.048 | 2.51E-02 | YES | 0.023 | 0.061 | YES | NO |
| 7q32.1 | **rs4728142** | 7.128573967 | ***IRF5*** | 7.128577991.128578103 | 0.234 | 0.051 | 5.53E-06 | 7.01E-04 | rs7807018 | 0.291 | 0.050 | 1.22E-08 | 0.420 | 0.041 | 6.51E-25 | YES | 0.042 | 0.051 | YES | NO |
|  |  |  |  | 7.128582125.128582330 | 0.361 | 0.049 | 9.28E-13 | 3.14E-10 | rs10239340 | 0.478 | 0.046 | 1.87E-22 | 0.662 | 0.041 | 4.28E-58 | YES | 0.048 | 0.051 | YES | NO |
|  |  |  |  | 7.128585899.128586088 | 0.418 | 0.047 | 5.42E-17 | 2.44E-14 | rs7807018 | 0.485 | 0.046 | 4.60E-23 | 0.726 | 0.042 | 9.08E-67 | YES | 0.047 | 0.051 | YES | NO |
|  |  |  |  | 7.128586555.128586996 | 0.312 | 0.050 | 9.56E-10 | 2.59E-07 | rs4731541 | 0.342 | 0.049 | 1.55E-11 | 0.534 | 0.041 | 2.14E-39 | YES | 0.043 | 0.052 | YES | NO |
|  |  |  |  | 7.128587077.128587589 | 0.414 | 0.048 | 1.06E-16 | 4.30E-14 | rs3807306 | 0.445 | 0.047 | 2.47E-19 | 0.035 | 0.028 | 2.04E-01 | NO | 0.044 | 0.066 | YES | YES |
|  |  |  |  | 7.128587783.128588175 | 0.464 | 0.046 | 4.50E-21 | 3.04E-18 | rs13242262 | 0.542 | 0.044 | 1.81E-29 | 0.783 | 0.044 | 2.29E-69 | YES | 0.050 | 0.061 | YES | NO |
|  |  |  |  | 7.128588264.128588382 | 0.434 | 0.047 | 2.36E-18 | 1.20E-15 | rs4731541 | 0.509 | 0.045 | 1.10E-25 | 0.765 | 0.042 | 7.59E-76 | YES | 0.048 | 0.059 | YES | NO |
| 7q32.1 | **rs10488631** | 7.128594183 | ***IRF5*** | 7.128587077.128587589 | 0.227 | 0.051 | 1.09E-05 | 1.26E-03 | rs3807306 | 0.445 | 0.047 | 2.47E-19 | 0.118 | 0.050 | 1.79E-02 | YES | 0.044 | 0.066 | YES | NO |
|  |  |  |  | 7.128587783.128588175 | 0.200 | 0.051 | 1.10E-04 | 8.73E-03 | rs13242262 | 0.542 | 0.044 | 1.81E-29 | 0.299 | 0.051 | 3.82E-09 | YES | 0.050 | 0.061 | YES | NO |
|  |  |  |  | 7.128588264.128588382 | 0.185 | 0.051 | 3.65E-04 | 2.46E-02 | rs4731541 | 0.509 | 0.045 | 1.10E-25 | 0.283 | 0.051 | 2.46E-08 | YES | 0.048 | 0.059 | YES | NO |
| 8p23.1 | **rs2736340** | 8.11343973 | ***BLK*** | 8.11351510.11352100 | 0.240 | 0.051 | 3.15E-06 | 4.26E-04 | rs2618451 | 0.291 | 0.050 | 1.37E-08 | 0.100 | 0.046 | 2.81E-02 | YES | 0.068 | 0.063 | NO | NO |
|  |  |  |  | 8.11400733.11400856 | 0.297 | 0.050 | 6.20E-09 | 1.40E-06 | rs2736340 | 0.297 | 0.050 | 6.20E-09 | 0.000 | 0.000 | 0.00E+00 | NO | 0.066 | 0.076 | YES | YES |
|  |  |  |  | 8.11403240.11403612 | 0.268 | 0.050 | 1.74E-07 | 2.94E-05 | rs2736340 | 0.268 | 0.050 | 1.74E-07 | 0.000 | 0.000 | 0.00E+00 | NO | 0.065 | 0.067 | YES | YES |
|  |  |  |  | 8.11405541.11405634 | 0.267 | 0.050 | 2.01E-07 | 3.26E-05 | rs2618476 | 0.272 | 0.050 | 1.18E-07 | 0.000 | 0.000 | 0.00E+00 | NO | 0.066 | 0.073 | YES | YES |
|  |  |  |  | 8.11406533.11406631 | 0.232 | 0.051 | 7.17E-06 | 8.81E-04 | rs11990277 | 0.257 | 0.051 | 5.99E-07 | 0.062 | 0.039 | 1.15E-01 | NO | 0.067 | 0.058 | NO | NO |
|  |  |  |  | 8.11407503.11407771 | 0.218 | 0.051 | 2.41E-05 | 2.44E-03 | rs2736340 | 0.218 | 0.051 | 2.41E-05 | 0.000 | 0.000 | 0.00E+00 | NO | 0.065 | 0.060 | NO | NO |
|  |  |  |  | 8.11412252.11412398 | 0.282 | 0.050 | 3.72E-08 | 6.86E-06 | rs12680762 | 0.292 | 0.050 | 1.12E-08 | 0.049 | 0.031 | 1.18E-01 | NO | 0.065 | 0.076 | YES | YES |
|  |  |  |  | 8.11412841.11412993 | 0.301 | 0.050 | 3.80E-09 | 9.63E-07 | rs1478901 | 0.307 | 0.050 | 1.77E-09 | 0.000 | 0.000 | 0.00E+00 | NO | 0.065 | 0.080 | YES | YES |
|  |  |  |  | 8.11414166.11414346 | 0.290 | 0.050 | 1.50E-08 | 3.04E-06 | rs2618476 | 0.297 | 0.050 | 5.95E-09 | 0.000 | 0.000 | 0.00E+00 | NO | 0.067 | 0.075 | YES | YES |
|  |  |  |  | 8.11415471.11415547 | 0.264 | 0.050 | 2.75E-07 | 4.29E-05 | rs998683 | 0.275 | 0.050 | 8.65E-08 | 0.000 | 0.000 | 0.00E+00 | NO | 0.065 | 0.070 | YES | YES |
|  |  |  |  | 8.11417842.11418961 | 0.373 | 0.048 | 1.26E-13 | 4.65E-11 | rs2618476 | 0.392 | 0.048 | 6.06E-15 | 0.000 | 0.000 | 0.00E+00 | NO | 0.070 | 0.110 | YES | YES |
|  |  |  |  | 8.11420488.11420619 | 0.334 | 0.049 | 4.58E-11 | 1.33E-08 | rs998683 | 0.354 | 0.049 | 2.83E-12 | 0.000 | 0.000 | 0.00E+00 | NO | 0.065 | 0.094 | YES | YES |
|  |  |  |  | 8.11421412.11422113 | 0.344 | 0.049 | 1.16E-11 | 3.62E-09 | rs998683 | 0.354 | 0.049 | 2.53E-12 | 0.000 | 0.000 | 0.00E+00 | NO | 0.068 | 0.094 | YES | YES |
|  |  |  | ***FAM167A*** | 8.11278972.11282145 | 0.523 | 0.045 | 2.96E-27 | 6.00E-24 | rs1478901 | 0.524 | 0.045 | 2.57E-27 | 0.000 | 0.000 | 0.00E+00 | NO | 0.056 | 0.308 | YES | YES |
|  |  |  | ***FDFT1*** | 8.11665926.11666400 | 0.176 | 0.051 | 6.76E-04 | 4.15E-02 | rs1616534 | 0.368 | 0.049 | 2.91E-13 | 0.296 | 0.049 | 1.97E-09 | YES | 0.063 | 0.052 | NO | NO |
|  |  |  | ***RP11-148O21.2*** | 8.11415975.11416256 | 0.203 | 0.051 | 9.13E-05 | 7.56E-03 | rs11250157 | 0.234 | 0.051 | 5.55E-06 | 0.106 | 0.047 | 5.55E-02 | NO | 0.058 | 0.062 | YES | YES |
|  |  |  |  | 8.11417293.11417529 | 0.222 | 0.051 | 1.79E-05 | 1.96E-03 | rs922483 | 0.227 | 0.051 | 1.14E-05 | 0.026 | 0.026 | 3.25E-01 | NO | 0.059 | 0.061 | YES | YES |
|  |  |  | ***RP11-148O21.4*** | 8.11413760.11414170 | 0.220 | 0.051 | 2.09E-05 | 2.23E-03 | rs1478901 | 0.220 | 0.051 | 1.99E-05 | 0.000 | 0.000 | 0.00E+00 | NO | 0.059 | 0.059 | YES | YES |
| 10q11.23 | **rs2663052** | 10.50069395 | ***WDFY4*** | 10.50015899.50016263 | 0.174 | 0.051 | 7.97E-04 | 4.82E-02 | rs7078312 | 0.204 | 0.051 | 7.87E-05 | 0.000 | 0.000 | 0.00E+00 | NO | 0.067 | 0.056 | NO | NO |
|  |  |  |  | 10.50018575.50018780 | 0.194 | 0.051 | 1.76E-04 | 1.37E-02 | rs7072606 | 0.234 | 0.051 | 5.59E-06 | 0.177 | 0.053 | 7.62E-04 | YES | 0.067 | 0.055 | NO | NO |
| 11p13 | **rs2732549** | 11.35088399 | ***APIP*** | 11.34909840.34909975 | 0.181 | 0.051 | 4.75E-04 | 3.11E-02 | rs2915228 | 0.204 | 0.051 | 8.26E-05 | 0.214 | 0.052 | 3.66E-05 | YES | 0.084 | 0.053 | NO | NO |
| 11p15.5 | **rs2396545** | 11.601785 | ***ANO9*** | 11.419582.420860 | 0.180 | 0.051 | 5.20E-04 | 3.35E-02 | rs4963176 | 0.287 | 0.050 | 2.07E-08 | 0.053 | 0.047 | 2.55E-01 | NO | 0.024 | 0.056 | YES | YES |
|  |  |  | ***HRAS*** | 11.532242.532755 | 0.179 | 0.051 | 5.75E-04 | 3.64E-02 | rs7930593 | 0.200 | 0.051 | 1.13E-04 | 0.092 | 0.047 | 5.01E-02 | NO | 0.026 | 0.056 | YES | YES |
|  |  |  | ***IRF7*** | 11.612553.612800 | 0.201 | 0.051 | 1.03E-04 | 8.31E-03 | rs3758650 | 0.213 | 0.051 | 3.90E-05 | 0.220 | 0.052 | 2.18E-05 | YES | 0.027 | 0.058 | YES | NO |
|  |  |  |  | 11.612999.613595 | 0.214 | 0.051 | 3.41E-05 | 3.22E-03 | rs2894649 | 0.274 | 0.050 | 9.77E-08 | 0.244 | 0.052 | 2.44E-06 | YES | 0.027 | 0.062 | YES | NO |
|  |  |  |  | 11.613785.614534 | 0.263 | 0.050 | 3.02E-07 | 4.54E-05 | rs702966 | 0.303 | 0.050 | 3.06E-09 | -0.016 | 0.020 | 4.08E-01 | NO | 0.027 | 0.074 | YES | YES |
|  |  |  |  | 11.614783.615728 | 0.216 | 0.051 | 2.89E-05 | 2.86E-03 | rs702966 | 0.245 | 0.051 | 1.93E-06 | -0.010 | 0.020 | 6.03E-01 | NO | 0.027 | 0.063 | YES | YES |
|  |  |  | ***RNH1*** | 11.500847.503570 | 0.284 | 0.050 | 2.84E-08 | 5.49E-06 | rs12792111 | 0.480 | 0.046 | 1.26E-22 | 0.121 | 0.049 | 1.38E-02 | YES | 0.027 | 0.072 | YES | NO |
|  |  |  |  | 11.504824.505881 | 0.238 | 0.051 | 3.84E-06 | 5.02E-04 | rs11821341 | 0.473 | 0.046 | 6.28E-22 | 0.009 | 0.045 | 8.35E-01 | NO | 0.028 | 0.067 | YES | YES |
|  |  |  | ***TALDO1*** | 11.758950.759057 | 0.186 | 0.051 | 3.44E-04 | 2.36E-02 | rs7117022 | 0.197 | 0.051 | 1.37E-04 | 0.014 | 0.026 | 5.82E-01 | NO | 0.027 | 0.055 | YES | YES |
|  |  |  | ***TMEM80*** | 11.702945.704129 | 0.297 | 0.050 | 6.06E-09 | 1.40E-06 | rs11246262 | 0.466 | 0.046 | 3.44E-21 | 0.434 | 0.049 | 1.60E-18 | YES | 0.029 | 0.089 | YES | NO |
| 11q13.4 | **rs3794060** | 11.71187679 | ***NADSYN1*** | 11.71169474.71170102 | 0.215 | 0.051 | 3.09E-05 | 2.98E-03 | rs2852853 | 0.219 | 0.051 | 2.31E-05 | 0.015 | 0.021 | 4.64E-01 | NO | 0.019 | 0.022 | YES | YES |
|  |  |  |  | 11.71185441.71186668 | 0.526 | 0.044 | 1.28E-27 | 5.19E-24 | rs7928249 | 0.527 | 0.044 | 1.03E-27 | 0.000 | 0.000 | 0.00E+00 | NO | 0.023 | 0.092 | YES | YES |
|  |  |  |  | 11.71187079.71188484 | 0.512 | 0.045 | 6.30E-26 | 8.52E-23 | rs7928249 | 0.512 | 0.045 | 5.86E-26 | 0.000 | 0.000 | 0.00E+00 | NO | 0.022 | 0.083 | YES | YES |
|  |  |  |  | 11.71190340.71191320 | 0.230 | 0.051 | 8.16E-06 | 9.73E-04 | rs7122671 | 0.233 | 0.051 | 6.13E-06 | 0.193 | 0.052 | 1.90E-04 | YES | 0.019 | 0.022 | YES | NO |
|  |  |  | ***RP11-660L16.2*** | 11.71159720.71159931 | 0.437 | 0.047 | 1.48E-18 | 8.58E-16 | rs12419279 | 0.437 | 0.047 | 1.23E-18 | 0.031 | 0.020 | 1.19E-01 | NO | 0.022 | 0.078 | YES | YES |
|  |  |  |  | 11.71162736.71163203 | 0.508 | 0.045 | 1.54E-25 | 1.56E-22 | rs2282621 | 0.508 | 0.045 | 1.50E-25 | 0.000 | 0.000 | 0.00E+00 | NO | 0.022 | 0.078 | YES | YES |
| 15q24.2 | **rs2289583** | 15.75311036 | ***CSK*** | 15.75094672.75095539 | 0.204 | 0.051 | 8.17E-05 | 7.05E-03 | rs2472304 | 0.344 | 0.049 | 1.23E-11 | 0.042 | 0.046 | 3.56E-01 | NO | 0.018 | 0.047 | YES | YES |
|  |  |  | ***MPI*** | 15.75189853.75191798 | 0.252 | 0.051 | 1.02E-06 | 1.43E-04 | rs7495739 | 0.364 | 0.049 | 6.08E-13 | 0.090 | 0.047 | 5.45E-02 | NO | 0.018 | 0.037 | YES | YES |
|  |  |  | ***SCAMP2*** | 15.75142855.75143014 | 0.188 | 0.051 | 2.83E-04 | 2.05E-02 | rs7495739 | 0.279 | 0.050 | 5.00E-08 | 0.061 | 0.046 | 1.88E-01 | NO | 0.017 | 0.031 | YES | YES |
|  |  |  | ***ULK3*** | 15.75128459.75129585 | 0.259 | 0.050 | 4.62E-07 | 6.69E-05 | rs9210 | 0.436 | 0.047 | 1.53E-18 | -0.001 | 0.042 | 9.80E-01 | NO | 0.019 | 0.048 | YES | YES |
|  |  |  |  | 15.75132839.75132982 | 0.189 | 0.051 | 2.58E-04 | 1.94E-02 | rs8042694 | 0.297 | 0.050 | 6.15E-09 | -0.015 | 0.038 | 7.02E-01 | NO | 0.017 | 0.034 | YES | YES |
|  |  |  |  | 15.75134621.75134761 | 0.194 | 0.051 | 1.85E-04 | 1.42E-02 | rs2301249 | 0.311 | 0.050 | 1.03E-09 | 0.024 | 0.044 | 5.86E-01 | NO | 0.017 | 0.035 | YES | YES |
| 17p13.2 | **rs2286672** | 17.4712617 | ***PSMB6*** | 17.4700733.4700864 | 0.223 | 0.051 | 1.54E-05 | 1.74E-03 | rs2302327 | 0.224 | 0.051 | 1.39E-05 | 0.045 | 0.031 | 1.53E-01 | NO | 0.007 | 0.006 | NO | NO |
|  |  |  |  | 17.4700977.4701106 | 0.211 | 0.051 | 4.33E-05 | 3.99E-03 | rs2302327 | 0.229 | 0.051 | 8.75E-06 | 0.029 | 0.031 | 3.59E-01 | NO | 0.007 | 0.006 | NO | NO |
| 22q11.21 | **rs7444** | 22.21976934 | ***UBE2L3*** | 22.21975804.21978323 | 0.505 | 0.045 | 3.68E-25 | 2.99E-22 | rs4821124 | 0.506 | 0.045 | 2.39E-25 | 0.000 | 0.000 | 0.00E+00 | NO | 0.040 | 0.278 | YES | YES |

**
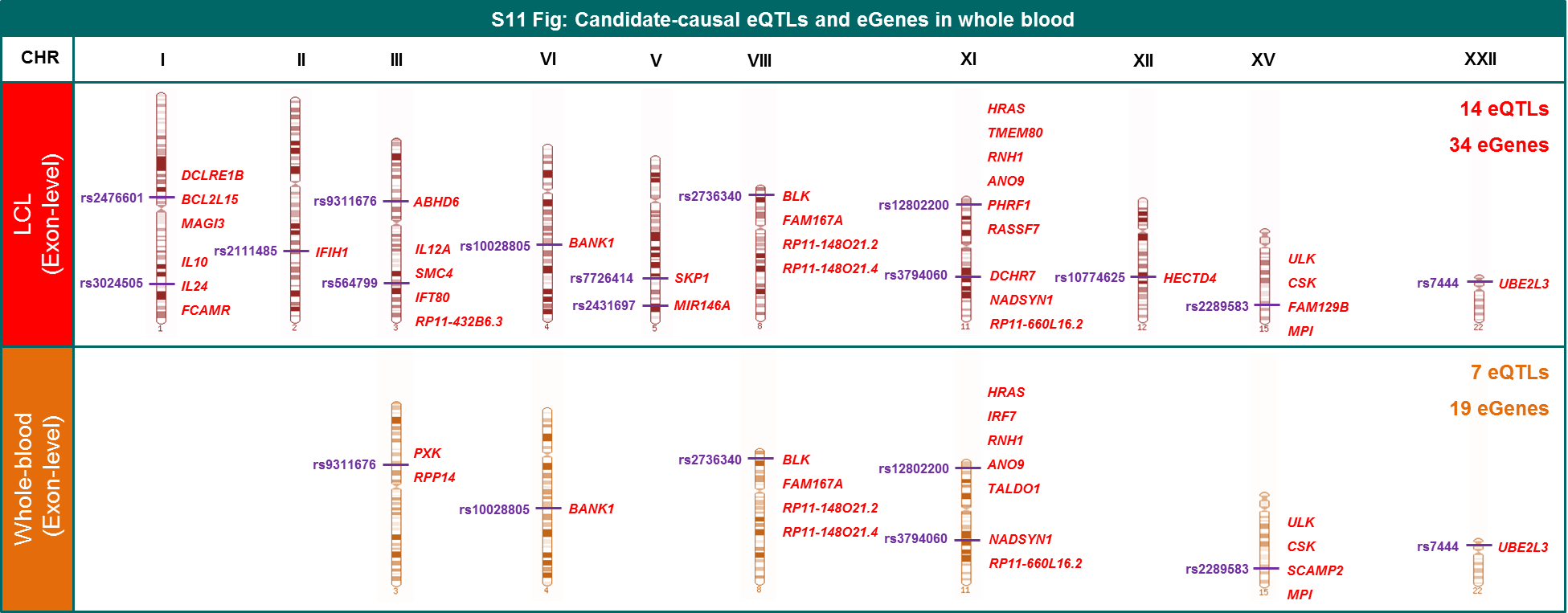
 Supplementary Material, Figure S3:** Candidate-causal eQTLs and eGenes in Whole-blood

**
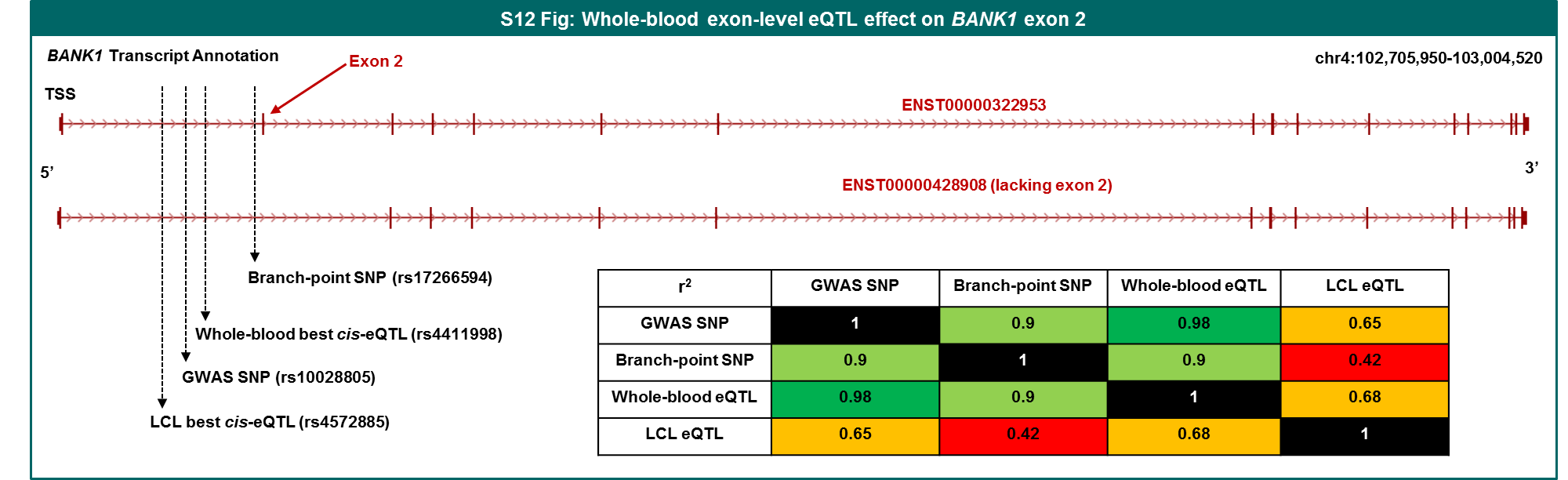
** **Supplementary Material, Figure S4:** Whole-blood exon-level eQTL effect on *BANK1* exon 2

| **Supplementary Material, Table S7**  **Validation of candidate-causal eQTLs from TwinsUK exon-level analysis in the GTEx whole-blood cohort** | | | |
| --- | --- | --- | --- |
| **GWAS SNP eQTL** | **eGene** | **P-Value (TwinsUK) Whole-Blood** | **P-Value (GTEx) Whole-Blood** |
| rs9311676 | *PXK* | 1.15E-07 | 4.00E-05 |
| rs9311676 | *RPP14* | 4.52E-05 | 9.50E-06 |
| rs10028805 | *BANK1* | 8.41E-05 | NA |
| rs2736340 | *BLK* | 1.16E-11 | 3.30E-10 |
| rs2736340 | *FAM167A* | 2.96E-27 | 3.40E-24 |
| rs2736340 | *RP11-148O21.2* | 1.79E-05 | NA |
| rs2736340 | *RP11-148O21.4* | 2.09E-05 | NA |
| rs12802200 | *ANO9* | 5.20E-04 | NA |
| rs12802200 | *HRAS* | 5.75E-04 | NA |
| rs12802200 | *IRF7* | 3.02E-07 | NA |
| rs12802200 | *RNH1* | 3.84E-06 | NA |
| rs12802200 | *TALDO1* | 3.44E-04 | NA |
| rs3794060 | *NADSYN1* | 1.28E-27 | 2.80E-23 |
| rs3794060 | *RP11-660L16.2* | 1.54E-25 | 2.40E-30 |
| rs2289583 | *CSK* | 8.17E-05 | NA |
| rs2289583 | *MPI* | 1.02E-06 | 2.70E-08 |
| rs2289583 | *SCAMP2* | 2.83E-04 | NA |
| rs2289583 | *ULK* | 4.62E-07 | NA |
| rs7444 | *UBE2L3* | 3.68E-25 | 1.70E-07 |

**
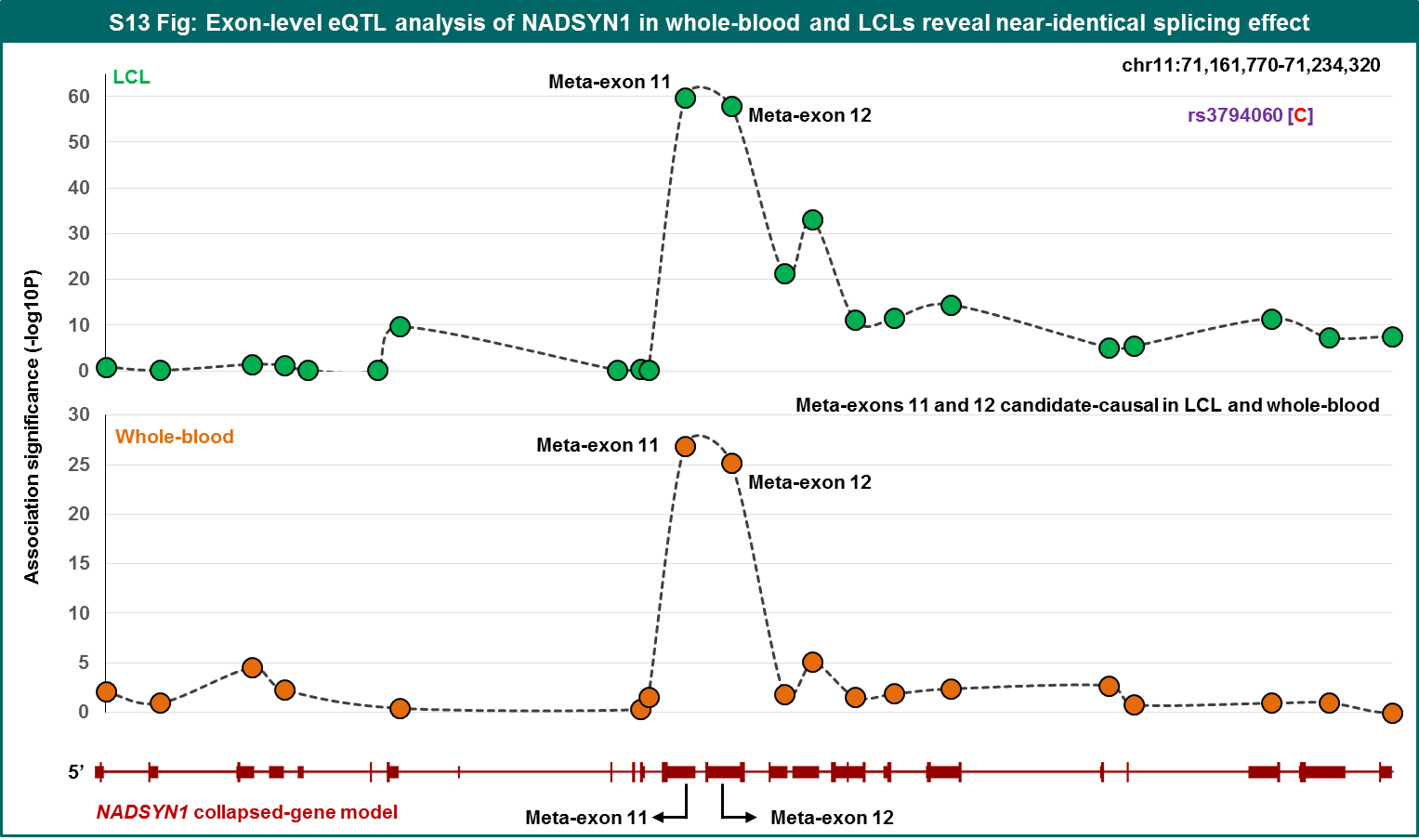
 Supplementary Material, Figure S5:** Exon-level eQTL analysis of NADSYN1 in whole-blood and LCLs reveals near-identical splicing effect

| **Supplementary Material, Table S8**  All significant asQTLs (q < 0.05) and associated eGenes detected at RNA-Seq (splice-junction level) | | | | | | | | | | | | | | | |
| --- | --- | --- | --- | --- | --- | --- | --- | --- | --- | --- | --- | --- | --- | --- | --- |
| **GWAS SNP** | **hg19 position chr: bp** | **eGene** | **Spice Junction (chr: donor-acceptor exon)** | **β** | **Std Err** | **P-Value** | **FDR (q)** | **best HotSpot**  **eQTL** | **hg19 position chr: bp** | **β** | **Std Err** | **P-Value** | **RTC Score** | **Corr.** | **Rank** |
| rs2476601 | 1:114377568 | *PHTF1* | 1:114240884.114241009-114239827.114240347 | 8.47E-02 | 1.46E-02 | 1.36E-08 | 1.64E-05 | rs10858008 | 1:114119620 | 9.37E-02 | 7.92E-03 | 1.36E-27 | 0.59 | 0.23 | 442.5 |
|  |  |  | 1:114240884.114241009-114242326.114242421 | -8.75E-02 | 1.49E-02 | 1.01E-08 | 1.36E-05 | rs1935838 | 1:114317791 | -9.12E-02 | 8.31E-03 | 2.07E-24 | 0.59 | 0.27 | 442.5 |
| rs6736175 | 2:191946322 | *STAT1* | 2:191829084.191829420-191827556.191827884 | 2.33E-02 | 4.91E-03 | 2.89E-06 | 2.14E-03 | rs3088307 | 2:191829412 | 6.93E-02 | 3.78E-03 | 7.00E-54 | 0.45 | 0.12 | 340.5 |
|  |  |  | 2:191829084.191829420-191839556.191839661 | 4.42E-02 | 7.90E-03 | 4.32E-08 | 4.76E-05 | rs3088307 | 2:191829412 | 1.26E-01 | 5.39E-03 | 1.15E-74 | 0.46 | 0.12 | 336.5 |
|  |  |  | 2:191829084.191829420-191840368.191840613 | 2.52E-02 | 4.55E-03 | 6.03E-08 | 6.43E-05 | rs3088307 | 2:191829412 | 7.42E-02 | 3.01E-03 | 6.21E-80 | 0.46 | 0.12 | 335.5 |
|  |  |  | 2:191829084.191829420-191841566.191841751 | 2.91E-02 | 5.34E-03 | 8.65E-08 | 8.82E-05 | rs3088307 | 2:191829412 | 8.68E-02 | 3.52E-03 | 9.51E-80 | 0.46 | 0.12 | 334.5 |
|  |  |  | 2:191835224.191835443-191827556.191827896 | 2.45E-02 | 5.66E-03 | 1.88E-05 | 1.06E-02 | rs3088307 | 2:191829412 | 7.31E-02 | 4.63E-03 | 3.01E-43 | 0.45 | 0.12 | 341.5 |
|  |  |  | 2:191835224.191835443-191862582.191862733 | 7.89E-02 | 1.54E-02 | 5.01E-07 | 4.47E-04 | rs3088307 | 2:191829412 | 2.09E-01 | 1.24E-02 | 1.16E-47 | 0.47 | 0.12 | 331.5 |
| rs3768792 | 2:213871709 | *IKZF2* | 2:213886368.213886444-213872084.213872808 | -4.07E-02 | 6.73E-03 | 3.64E-09 | 5.51E-06 | rs9808132 | 2:213877864 | -4.24E-02 | 6.73E-03 | 8.71E-10 | 0.98 | 0.98 | 5 |
|  |  |  | 2:213886368.213886444-213878515.213880002 | -3.28E-02 | 6.66E-03 | 1.30E-06 | 1.07E-03 | rs9808132 | 2:213877864 | -3.44E-02 | 6.66E-03 | 4.02E-07 | 0.98 | 0.98 | 5 |
|  |  |  | 2:213886368.213886444-213881647.213881768 | -3.54E-02 | 6.61E-03 | 1.48E-07 | 1.41E-04 | rs9808132 | 2:213877864 | -3.69E-02 | 6.62E-03 | 4.93E-08 | 0.98 | 0.98 | 5 |
|  |  |  | 2:213886368.213886444-213886717.213886854 | 6.59E-02 | 1.58E-02 | 3.80E-05 | 1.93E-02 | rs9808132 | 2:213877864 | 6.90E-02 | 1.58E-02 | 1.70E-05 | 0.98 | 0.98 | 5 |
|  |  |  | 2:213886368.213886444-213914437.213914604 | -3.52E-02 | 7.57E-03 | 4.55E-06 | 3.15E-03 | rs13000467 | 2:213862922 | -3.52E-02 | 7.23E-03 | 1.63E-06 | 0.98 | 0.85 | 6 |
| rs7726414 | 5:133431834 | *TCF7* | 5:133474642.133474729-133478412.133478791 | 1.44E-02 | 2.97E-03 | 1.90E-06 | 1.49E-03 | rs28578492 | 5:133424804 | 1.37E-02 | 2.63E-03 | 3.29E-07 | 0.98 | 0.80 | 1.5 |
|  |  | *SKP1* | 5:133561451.133561762-133541305.133541822 | 2.26E-02 | 4.61E-03 | 1.42E-06 | 1.16E-03 | rs73790123 | 5:133425768 | 2.34E-02 | 4.64E-03 | 7.42E-07 | 0.92 | 0.98 | 5.5 |
|  |  |  | 5:133561451.133561762-133541645.133541822 | -3.25E-02 | 6.60E-03 | 1.28E-06 | 1.07E-03 | rs4388254 | 5:133428601 | -3.25E-02 | 6.60E-03 | 1.28E-06 | 0.99 | 1.00 | 0.5 |
| rs3757387 | 7:128576086 | *IRF5* | 7:128577666.128577888-128585899.128586088 | 2.40E-02 | 4.20E-03 | 2.18E-08 | 2.49E-05 | rs2004640 | 7:128578301 | 2.41E-02 | 4.18E-03 | 1.60E-08 | 0.99 | 0.66 | 6 |
|  |  |  | 7:128577666.128577888-128586555.128586616 | 4.90E-02 | 9.83E-04 | 9.51E-07 | 8.13E-04 | rs3757387 | 7:128576086 | 4.90E-03 | 9.83E-04 | 9.51E-07 | 1.00 | 1.00 | 0 |
| rs2736340 | 8:11343973 | *BLK* | 8:11403240.11403612-11407668.11407771 | 1.73E-02 | 2.79E-03 | 1.74E-09 | 3.14E-06 | rs2736345 | 8:11352485 | 1.73E-02 | 2.63E-03 | 1.41E-10 | 0.98 | 0.78 | 12.5 |
|  |  |  | 8:11403240.11403612-11412252.11412398 | 1.69E-02 | 2.78E-03 | 2.77E-09 | 4.61E-06 | rs2736345 | 8:11352485 | 1.71E-02 | 2.61E-03 | 1.76E-10 | 0.98 | 0.78 | 12.5 |
|  |  |  | 8:11403561.11403612-11351510.11352100 | 9.97E-02 | 2.40E-02 | 4.16E-05 | 2.08E-02 | rs7828916 | 8:11402063 | 1.07E-01 | 2.19E-02 | 1.55E-06 | 0.93 | 0.47 | 46.5 |
|  |  |  | 8:11403561.11403612-11400733.11400856 | -4.18E-02 | 1.03E-02 | 6.24E-05 | 2.96E-02 | rs7828916 | 8:11402063 | -4.61E-02 | 9.40E-03 | 1.43E-06 | 0.94 | 0.47 | 43.5 |
|  |  |  | 8:11403561.11403612-11405541.11405634 | -5.14E-02 | 1.21E-02 | 2.67E-05 | 1.46E-02 | rs55860742 | 8:11358156 | -4.91E-02 | 1.00E-02 | 1.51E-06 | 0.79 | 0.36 | 138.5 |
|  |  |  | 8:11407503.11407771-11414167.11414297 | 1.16E-02 | 1.99E-03 | 1.36E-08 | 1.64E-05 | rs62489069 | 8:11338383 | 1.12E-02 | 1.90E-03 | 9.19E-09 | 0.99 | 0.84 | 8.5 |
|  |  |  | 8:11407503.11407771-11415471.11415547 | 1.12E-02 | 2.34E-03 | 2.52E-06 | 1.90E-03 | rs62489069 | 8:11338383 | 1.07E-02 | 2.23E-03 | 2.19E-06 | 0.99 | 0.84 | 8.5 |
|  |  |  | 8:11414166.11414346-11415471.11415547 | -4.10E-02 | 8.67E-03 | 3.24E-06 | 2.32E-03 | rs2618476 | 8:11352541 | -4.27E-02 | 8.57E-03 | 9.61E-07 | 0.99 | 0.96 | 7.5 |
|  |  |  | 8:11414166.11414346-11420488.11420619 | 2.32E-02 | 3.00E-03 | 1.09E-13 | 7.06E-10 | rs2736337 | 8:11341880 | 2.34E-02 | 3.00E-03 | 7.31E-14 | 0.99 | 0.99 | 5.5 |
|  |  |  | 8:11417842.11418961-11421412.11422037 | 5.82E-02 | 1.07E-02 | 8.68E-08 | 8.82E-05 | rs6980884 | 8:11362275 | 5.34E-02 | 8.78E-03 | 2.92E-09 | 0.82 | 0.37 | 119.5 |
|  |  |  | 8:11418811.11418961-11351510.11352100 | 8.75E-02 | 1.00E-02 | 9.56E-17 | 1.55E-12 | rs6980884 | 8:11362275 | 7.50E-02 | 8.28E-03 | 7.98E-18 | 0.84 | 0.37 | 106.5 |
|  |  |  | 8:11418811.11418961-11415471.11415547 | -3.17E-02 | 4.60E-03 | 2.42E-11 | 5.71E-08 | rs6980884 | 8:11362275 | -2.66E-02 | 3.82E-03 | 1.58E-11 | 0.84 | 0.37 | 104.5 |
|  |  |  | 8:11418811.11418961-11420488.11420619 | -5.39E-02 | 5.86E-03 | 2.85E-18 | 6.18E-14 | rs6980884 | 8:11362275 | -4.67E-02 | 4.82E-03 | 7.18E-20 | 0.84 | 0.37 | 110.5 |
| rs2663052 | 10:50069395 | *WDFY4* | 10:50030425.50030582-50031227.50032214 | -1.70E-02 | 1.79E-03 | 3.31E-19 | 2.15E-14 | rs2940727 | 10:50072102 | -1.95E-02 | 1.74E-03 | 4.32E-25 | 0.95 | 0.87 | 17 |
|  |  |  | 10:50030425.50030582-50034716.50034954 | 1.82E-02 | 3.84E-03 | 3.01E-06 | 2.17E-03 | rs2620904 | 10:50045021 | 1.96E-02 | 3.90E-03 | 7.55E-07 | 0.94 | 0.85 | 19 |
|  |  |  | 10:50031227.50032214-50028279.50028482 | 1.46E-02 | 2.51E-03 | 1.40E-08 | 1.66E-05 | rs10857653 | 10:50033848 | 2.06E-02 | 2.68E-03 | 1.30E-13 | 0.78 | 0.20 | 76 |
|  |  |  | 10:50031227.50032214-50029107.50029221 | 1.61E-02 | 3.60E-03 | 9.60E-06 | 6.12E-03 | rs2377655 | 10:50020326 | 3.55E-02 | 5.32E-03 | 8.91E-11 | 0.57 | 0.03 | 148 |
|  |  |  | 10:50031227.50032214-50030425.50030582 | -6.91E-02 | 1.27E-02 | 9.32E-08 | 9.33E-05 | rs10857653 | 10:50033848 | -1.10E-01 | 1.33E-02 | 1.96E-15 | 0.72 | 0.20 | 95 |
|  |  |  | 10:50031227.50032214-50036823.50036941 | 1.70E-02 | 2.88E-03 | 7.49E-09 | 1.06E-05 | rs10857653 | 10:50033848 | 2.37E-02 | 3.07E-03 | 1.07E-13 | 0.75 | 0.20 | 85 |
|  |  |  | 10:50031227.50032214-50075310.50075387 | 1.47E-02 | 2.30E-03 | 4.83E-10 | 9.52E-07 | rs10857653 | 10:50033848 | 2.11E-02 | 2.42E-03 | 9.92E-17 | 0.77 | 0.20 | 78 |
|  |  |  | 10:50108266.50108351-50109846.50109908 | -1.20E-02 | 2.65E-03 | 7.58E-06 | 4.98E-03 | rs3897964 | 10:50109028 | -3.37E-02 | 3.31E-03 | 1.21E-21 | 0.68 | 0.20 | 109 |
|  |  |  | 10:50149851.50149893-50109846.50109908 | -1.35E-02 | 1.87E-03 | 2.88E-12 | 9.36E-09 | rs2889698 | 10:50088284 | -2.69E-02 | 2.30E-03 | 4.50E-27 | 0.69 | 0.20 | 107 |
| rs3794060 | 11:71187679 | *NADSYN1* | 11:71171768.71171993-71169474.71169590 | 7.07E-02 | 1.69E-02 | 3.69E-05 | 1.89E-02 | rs148838851 | 11:71224042 | 9.26E-02 | 1.74E-02 | 1.81E-07 | 0.79 | 0.66 | 70 |
|  |  |  | 11:71171768.71171993-71171087.71171259 | -6.45E-02 | 1.52E-02 | 2.95E-05 | 1.55E-02 | rs4944949 | 11:71160395 | -6.53E-02 | 1.47E-02 | 1.26E-05 | 0.95 | 0.86 | 16 |
|  |  |  | 11:71185441.71185572-71189441.71189515 | 1.41E-02 | 3.41E-03 | 4.27E-05 | 2.10E-02 | rs12289358 | 11:71193681 | 2.66E-02 | 5.95E-03 | 1.02E-05 | 0.60 | 0.13 | 132 |
|  |  |  | 11:71185441.71186668-71187247.71188271 | -1.03E-02 | 1.28E-03 | 1.60E-14 | 1.49E-10 | rs11606033 | 11:71154204 | -1.05E-02 | 1.26E-03 | 2.30E-15 | 0.89 | 0.90 | 38 |
|  |  |  | 11:71185441.71186668-71187685.71188484 | 1.08E-02 | 2.35E-03 | 6.42E-06 | 4.30E-03 | rs1790330 | 11:71147825 | 1.29E-02 | 2.41E-03 | 1.65E-07 | 0.78 | 0.70 | 74 |
|  |  |  | 11:71187079.71188484-71184615.71184732 | 6.24E-02 | 1.59E-02 | 1.02E-04 | 4.51E-02 | rs11606612 | 11:71164047 | 6.37E-02 | 1.59E-02 | 7.10E-05 | 0.95 | 0.99 | 17 |
|  |  |  | 11:71187079.71188484-71185441.71186668 | -6.24E-02 | 1.59E-02 | 1.02E-04 | 4.51E-02 | rs11606612 | 11:71164047 | -6.37E-02 | 1.59E-02 | 7.10E-05 | 0.95 | 0.99 | 17 |
|  |  |  | 11:71187247.71188271-71184324.71184412 | 1.65E-01 | 2.34E-02 | 8.00E-12 | 2.08E-08 | rs4944949 | 11:71160395 | 1.69E-01 | 2.25E-02 | 4.33E-13 | 0.92 | 0.86 | 27 |
|  |  |  | 11:71187247.71188271-71185441.71186668 | -1.90E-01 | 2.38E-02 | 2.13E-14 | 1.73E-10 | rs4944949 | 11:71160395 | -1.96E-01 | 2.28E-02 | 2.77E-16 | 0.92 | 0.86 | 27 |
|  |  |  | 11:71189441.71189515-71185441.71185572 | 2.32E-02 | 5.21E-03 | 1.06E-05 | 6.71E-03 | rs11604642 | 11:71222355 | 2.43E-02 | 5.12E-03 | 2.95E-06 | 0.92 | 0.97 | 27 |
| rs2289583 | 15:75311036 | *ULK3* | 15:75129710.75129776-75130092.75130139 | 7.90E-02 | 1.05E-02 | 4.43E-13 | 1.80E-09 | rs34965545 | 15:75281132 | 1.01E-01 | 9.89E-03 | 7.16E-22 | 0.83 | 0.34 | 62.5 |
|  |  |  | 15:75130092.75130139-75129710.75129776 | 6.32E-02 | 1.12E-02 | 3.41E-08 | 3.83E-05 | rs34965545 | 15:75281132 | 8.96E-02 | 1.06E-02 | 8.27E-16 | 0.83 | 0.34 | 62.5 |
|  |  |  | 15:75130493.75130533-75130092.75130139 | 4.76E-02 | 9.29E-03 | 4.85E-07 | 4.38E-04 | rs34965545 | 15:75281132 | 6.12E-02 | 9.01E-03 | 4.64E-11 | 0.83 | 0.34 | 62.5 |

**Supplementary Material, Figure S6:** qPCR validation of asQTL rs3769792 against splice-site exon 6A-6B of *IKZF2*

**Supplementary Material, Table S9:** Power calculations between quantification types

| **N\R2** | **0.01** | **0.02** | **0.03** | **0.04** | **0.05** | **0.06** | **0.07** | **0.08** | **0.09** | **0.10** |
| --- | --- | --- | --- | --- | --- | --- | --- | --- | --- | --- |
| **683** | 0.19 | 0.59 | 0.86 | 0.97 | 0.99 | 1.00 | 1.00 | 1.00 | 1.00 | 1.00 |
| **765** | 0.24 | 0.68 | 0.91 | 0.98 | 1.00 | 1.00 | 1.00 | 1.00 | 1.00 | 1.00 |
| **777** | 0.25 | 0.69 | 0.92 | 0.99 | 1.00 | 1.00 | 1.00 | 1.00 | 1.00 | 1.00 |

For the TwinsUK comparison, the samples sizes are: 777 for microarray, 683 for gene-level RNA-Seq, and 765 for exon-level RNA-Seq. These individuals are a subset of the original pool of 856 individuals, so there is considerable overlap between samples within different analyses as we assume the samples are taken randomly. This is a well powered study for both RN-Seq and Microarray data, with negligible differences over the range of effects sizes observed. We have performed power calculations to estimate the differences in power between groups: From our microarray data we see a range of effect sizes (R2) over 0.05-0.8 for common SNPs (MAF > 0.05). R2 (R-squared) here refers to the proportion of variance in gene expression explained by genotype. For RNA-Seq data we see a similar range for common SNPs. In both cases the majority of R2 values were over the range 0.05-0.5 with a medium of 0.06. In the RNA-Seq data we find that over the known SLE associated loci a p-value threshold of 0.0005 ensures a FDR of 0.05. Below we show below power calculations over the lower range of effect sizes discussed above assuming a statistical size of 0.0005. This is a very high powered study for both RNA-Seq and Microarray data when the effect size (R2) is 0.05 or above. The difference in power for weak effects is not great. Power calculations are shown for eQTL analysis (linear regression) with samples sizes of 683 (gene-level RNA-Seq), 765 (Exon-level RNA-Seq) and 777 (Microarray) over a range of effect sizes (0.01 – 0.1).
